# Terminal Group Effects on Molecular Organization and Charge Extraction in Self-Assembled Monolayers/Perovskite Interfaces

**DOI:** 10.1021/acsmaterialsau.6c00070

**Published:** 2026-07-16

**Authors:** Arthur P. Machado, Felipe C. Braga, Carlos H. Stadtlober, Paulo E. Marchezi, Douglas S. Ribeiro, Murillo H. M. Rodrigues, Zeno C. Brandão, Diogo M. G. Neto, Caio C. Oliveira, Ana F. Nogueira

**Affiliations:** † Institute of Chemistry, University of Campinas (UNICAMP), Campinas, SP 13083-970, Brazil; ‡ Institute of Physics Gleb Wataghin, University of Campinas (UNICAMP), Campinas, SP 13083-859, Brazil

**Keywords:** self-assembled monolayers, perovskite solar cells, interfacial energetics, hole-transporting materials, molecular organization, charge extraction

## Abstract

The structural nature of self-assembled monolayers (SAMs) critically determines interfacial quality, film formation, and charge transport in inverted perovskite solar cells (iPSCs). While variations in SAM functional groups have been explored, these studies have focused on their impact on device performance metrics. In contrast, a fundamental understanding of how subtle molecular structural variations govern the molecular organization of SAMs, and how it modulates surface energy, interfacial properties, and the growth of perovskite films deposited atop these monolayers, remains limited. In addition, as perovskite technology advances toward industrial deployment, gaining fundamental insight into how SAMs govern film quality is essential for guiding the development of efficient and stable devices. Here, we systematically investigate the effects of SAM terminal groups (electron-withdrawing, neutral, and electron-donating) on the morphology and optoelectronic properties of perovskite films. Our results demonstrate that the electronic nature of the terminal group plays a decisive role in determining SAM molecular organization, which in turn directly affects interfacial hole extraction efficiency.

## Graphical abstract

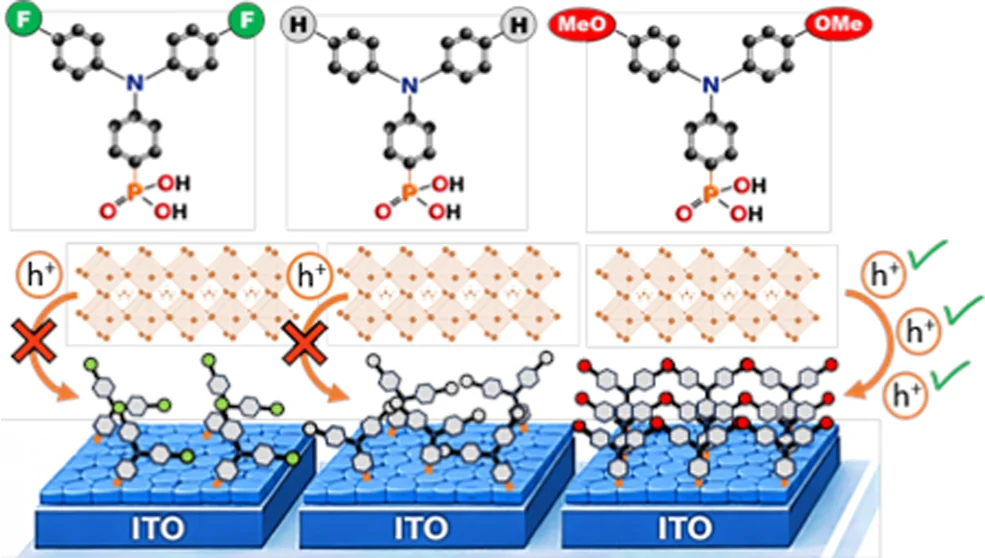

## Introduction

The modification of transparent conductive oxide (TCO) surfaces with organic molecules has been extensively investigated over the past two decades across various semiconductor-based technologies, including field effect transistors, light emitting diodes, and solar cells.^1–6^ The tunable functionality of these molecules allows precise modulation of interfacial and electronic properties, directly impacting device performances. In 2018, this molecular functionalization strategy was introduced to inverted perovskite solar cell technology (iPSCs),^7^ leading to the development of self-assembled monolayers (SAMs) as efficient hole-transporting materials (HTMs). Since then, numerous molecular structures have been synthesized and implemented in pursuit of enhanced iPSCs power conversion efficiencies (PCE %).^8–14^ However, with single junction perovskite solar cells now surpassing PCE % of 27%,^15–18^ research is increasingly focused on improving stability, reproducibility, and scalable fabrication under ambient, real-world conditions.^19–22^ In this context, understanding how SAMs affect perovskite film formation and interfacial quality under such conditions is becoming progressively important.

The fabrication of high performance iPSCs involves a complex interplay of variables that collectively determine device efficiency and stability. Factors such as perovskite composition, stoichiometry, interfacial compatibility, energy level alignment with adjacent charge transport layers, and the deposition methodology all play decisive roles in film quality and interfacial integrity.^23–29^ Although variations in SAM terminal groups have been explored, these efforts have predominantly focused on correlating molecular design with device performance metrics, often overlooking a fundamental understanding of how such modifications affect SAM organization and nanoscale interfacial characteristics. As a consequence, the structural origins of SAM-perovskite interactions remain insufficiently understood. In this study, we investigate how the electronic nature of SAM terminal groups influences the morphological and optoelectronic properties of perovskite films. Here, we introduce two novel TPA-based SAMs, F-TPAP and MeO-TPAP. Our findings reveal the impact of electron-donating groups on molecular organization, interfacial energetics, and charge extraction.

Based on the fundamental chemical properties and structural features of commonly used commercial SAMs, we hypothesized that an organic scaffold that combines synthetic simplicity and easy scalability could be designed around a triphenylamine core. Analogous to the commercially available SAM MeO-2PACz, triphenylamine (TPA) derivatives contain electron-rich arylamine units that are readily oxidized to form stable radical cations, thereby facilitating efficient hole transport.^30,31^ Furthermore, charge mobility and energy-level alignment with the perovskite layer could be finely tuned through structural modifications of the aromatic framework via the introduction of electron-donating or electron-withdrawing substituents. From a synthetic-standpoint, TPA scaffolds offer notable advantages over carbazole-based systems, which include broader functionalization, versatility and more straightforward coupling strategies.^32^ Thus, the combination of favorable electronic characteristics, accessible nitrogen oxidation, and synthetic flexibility motivated the selection of this core as a molecular platform for the development of new SAM-based HTMs for perovskite solar cells.

## Results and Discussion

Three SAM molecules were designed and synthesized based on a TPA core, a well-established structure in hole transporting material (HTM) engineering due to its favorable energy alignment with both the perovskite and ITO electrode.^8,18,33–37^ Each molecule was functionalized with phosphonic acid anchoring groups directly bonded to one of the phenyl rings of the TPA unit. This anchoring group was selected for its strong affinity toward oxide surfaces given its multiple hydroxyl sites that promote stable surface attachment and coverage.^1,9,38^ The molecular series varies in terminal substituents, allowing investigation of how the electronic character influences interfacial chemistry and perovskite film formation. Among the synthesized molecules, the terminal groups were varied between fluorine (R = F, F-TPAP), as an electron-withdrawing group, hydrogen (R = H, TPAP), representing a neutral substituent, and methoxy (R = OMe, MeO-TPAP), an electron-donating group. The F-TPAP and MeO-TPAP derivatives are novel compounds reported herein, with their synthetic routes illustrated in Figure 1a. Detailed synthetic procedures for each reaction step, along with full molecular characterization of all intermediates and final compounds, are provided in the Supporting Information (Figures S1–S33).

**Figure 1:**
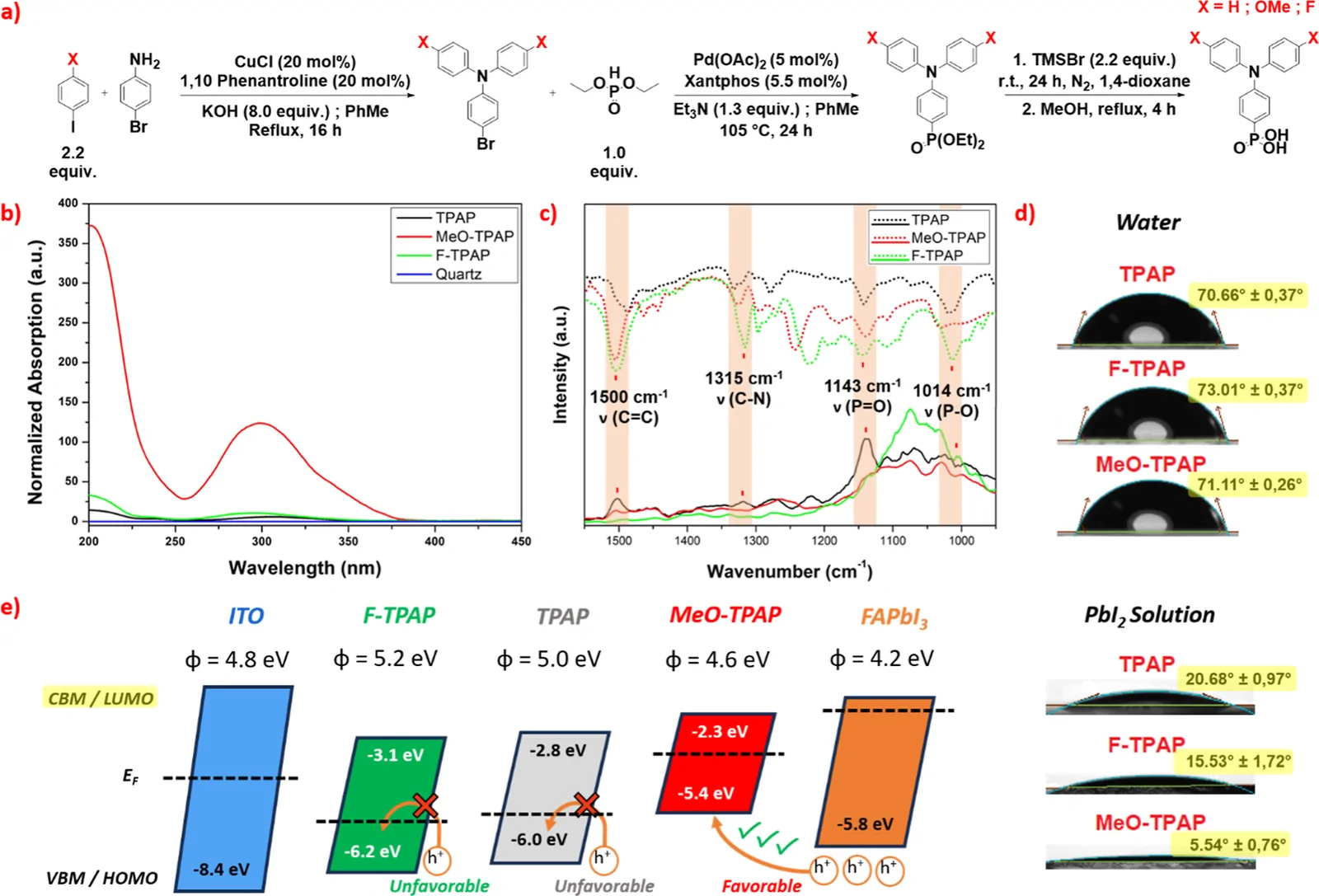
(a) Synthetic route for the TPA based self-assembled monolayers (SAMs). (b) UV–Vis absorption spectra of the SAM thin films. (c) FTIR spectra of TPA-based SAMs (dashed lines) and SAM-modified substrates films (solid lines). (d) Contact-angle measurements of the corresponding SAM-modified substrates with water and PbI_2_ solution. (e) Energy level diagram of ITO, FAPbI_3_ and SAM-modified substrates obtained from UPS.

The optical properties of the synthesized molecules in thin-film form were investigated by UV–Vis absorption spectroscopy. As shown in Figure 1b, all samples exhibit their main absorption features in the ultraviolet region, between 350 and 250 nm, which can be assigned to π–π* electronic transitions of the conjugated molecular frameworks.^39^ The optical energy gaps (*E*_g_), estimated from the absorption onset, show no significant differences among the samples, yielding values of 3.2, 3.1, and 3.2 eV for TPAP, F-TPAP, and MeO-TPAP, respectively (Figure S34). A comparison of the normalized spectra reveals that MeO-TPAP displays a relatively higher absorption intensity in the UV region compared to TPAP and F-TPAP. Although the absorption edge remains essentially unchanged, this variation in intensity may be associated with differences in molecular organization and intermolecular interactions, which are further investigated using complementary characterization techniques.

Figure 1c shows Fourier-transform infrared spectra (FT-IR) of the SAMs acquired from thin films (solid lines) and the corresponding powders (dashed lines). The bands at ∼1014 and ∼1145 cm^–1^ are assigned to ν­(P–O) and ν­(P=O) stretching modes of the phosphonic acid anchoring group, while absorptions at ∼1315 and ∼1500 cm^–1^ correspond to ν­(C–N) and ν­(C=C) vibrations of the triphenylamine core, respectively.^1,40,41^ The close agreement in band positions between powder and film spectra confirms preservation of the molecular structure upon film formation.

To further investigate the surface properties of the molecular films, water contact angle measurements were performed on SAM-modified ITO substrates (Figure 1d). Interestingly, all TPA derivatives exhibited negligible variation in hydrophobicity (∼70–72°), suggesting that the electronic nature of the terminal group has minimal impact on the macroscopic surface energy in this system. This trend stands in distinct contrast to the flexible 2PACz series reported by Merino et al.,^42^ where similar end group modifications within the 2PACz series produced substantial changes in the water contact angle. Therefore, it is plausible that in the PACz series, where the flexible alkyl linker enables conformational adaptability, the terminal functional groups exert a stronger influence on surface hydrophobicity. In contrast, in the more rigid and densely packed TPA-based systems, molecular organization dominates the interfacial behavior, thereby minimizing the impact of functional group electronic nature on the resulting water contact angle.

To assess the interaction between the SAM-modified surfaces and perovskite precursors, contact angle measurements were performed using a DMF/DMSO (9:1) solvent mixture and a PbI_2_ solution in the same solvent system. While the pure solvent mixture exhibited complete wetting on all substrates (contact angle <5°, Figure S35), the introduction of PbI_2_ revealed distinct chemical affinities. As shown in Figure 1d, MeO-TPAP exhibited the strongest affinity (5.54 ± 0.76°), superior to both the fluorinated (15.53 ± 1.52°) and neutral (20.68 ± 0.97°) analogs. We attribute this enhanced wettability to the Lewis basicity of the methoxy oxygen, which likely coordinates with Pb^2+^ species in solution. This result confirms that the terminal group does not merely dictate molecular organization but also modulates precursor affinity. The enhanced wettability of MeO-TPAP suggests a more favorable interaction with Pb-containing species, which may contribute to the improved film morphology observed for the resulting perovskite layers.

Ultraviolet photoelectron spectroscopy (UPS) was employed to quantify the impact of terminal groups on interfacial energetics (Figure 1e). The bare ITO reference exhibits a work function of 4.9 eV and a VBM of −8.4 eV.^1,43,44^ Upon modification, the electron-withdrawing F-TPAP and neutral TPAP deepen the HOMO levels to −6.2 eV and −6.0 eV, respectively. As noted in Figure 1e, these levels sit below the valence band maximum of FAPbI_3_ (−5.8 eV), creating a potential barrier for hole extraction. In contrast, the electron-donating MeO-TPAP shifts the work function to 4.6 eV and raises the HOMO to −5.4 eV. This favorable energetic offset with the perovskite suggests that electron-donating groups can effectively modulate the interfacial energetics, thereby facilitating charge transfer. The individual UPS spectra for each sample are provided in Figure S36 and all the energy level parameters are available at Table S1.

The molecular organization of the synthesized TPA-based monolayers was evaluated using atomic force microscopy coupled with infrared spectroscopy (AFM-IR), enabling simultaneous analysis of surface topography and chemical distribution. All SAM-modified substrates exhibit RMS roughness values very similar to that of bare ITO (Figure S37), with variations within ±0.23 nm (Figure 2a–c). Given the subnanometer height sensitivity of the measurement, these differences are detectable but remain small, indicating that none of the SAMs significantly modifies the native ITO topography.

**Figure 2:**
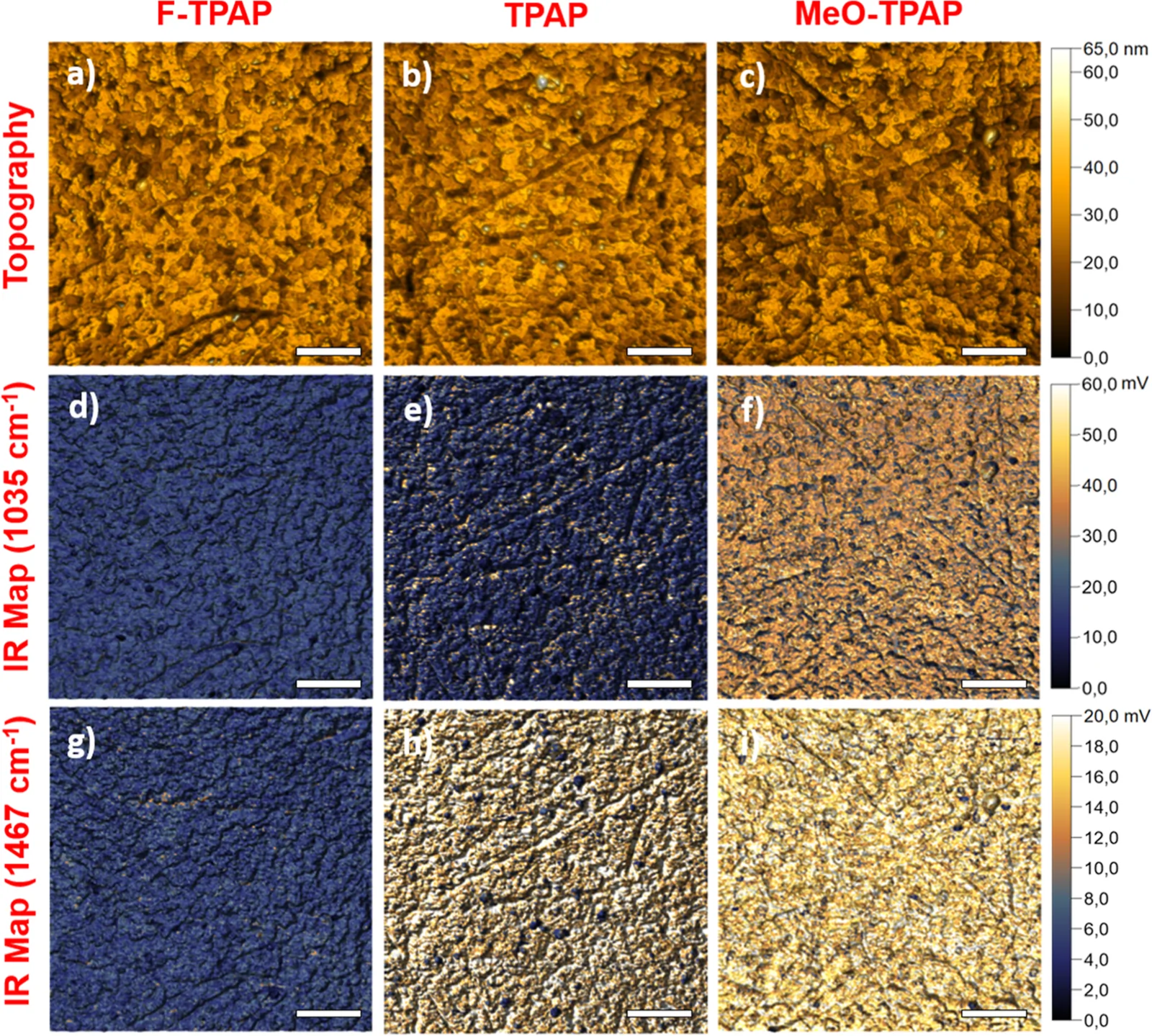
AFM topography **(a–c)** and IR absorption maps at 1035 cm^–1^ (P–O stretching, **(d–f)** and 1436 cm^–1^ (C=C stretching) **(g–i)** for the synthesized SAMs. Scale bars = 1 μm.

Figure 2d–f display the infrared mapping of the 1035 cm^–1^ band, assigned to the P–O stretching mode,^1,40,41^ enabling the visualization of the phosphonic acid anchoring distribution on the ITO surface. Among the series, MeO-TPAP exhibits the highest P–O signal intensity, indicating a more homogeneous spatial distribution of phosphonate groups. In contrast, the neutral TPAP derivative displays reduced signal intensity and increased spatial heterogeneity. Finally, the fluorinated derivative F-TPAP presents the weakest P–O response, suggesting a reduced effective infrared signal arising from differences in molecular organization and intermolecular interactions induced by the electron-withdrawing nature of the terminal group.

Finally, Figure 2g–i display the chemical maps corresponding to the 1467 cm^–1^ band, assigned to the C=C stretching of the phenyl rings,^41^ which serves as a probe for the spatial arrangement of the conjugated backbones. The MeO-TPAP sample exhibits the highest and most homogeneous signal intensity, fully consistent with the P–O maps, confirming its superior spatial uniformity. In contrast, TPAP displays larger signal fluctuations, indicative of increased nanoscale heterogeneity, whereas F-TPAP exhibits a weaker but comparatively more uniform response.

The differences in AFM-IR intensity should be interpreted as variations in the effective infrared response rather than as direct measurements of absolute molecular coverage. In AFM-IR, the detected signal originates from local infrared absorption followed by photothermal expansion of the sample, which is transferred to the AFM cantilever through the contact resonance mechanism.^45,46^ Consequently, the measured intensity depends not only on the number of absorbing groups, but also on thermal and mechanical properties, molecular orientation, and the efficiency with which the absorbed energy is converted into out-of-plane expansion.^45,46^ In this context, the high intensity and low spatial variability observed for MeO-TPAP suggest a highly organized molecular arrangement, consistent with enhanced intermolecular interactions promoted by the electron-donating methoxy substituent.

In contrast, the neutral TPAP derivative exhibits intermediate average intensities accompanied by the largest spatial fluctuations, suggesting that, in the absence of strong electronic perturbations, the molecular organization is governed primarily by the intrinsic characteristics of the TPA scaffold, resulting in increased nanoscale heterogeneity. Conversely, F-TPAP exhibits a uniformly weaker response despite its comparatively low spatial variability, indicating that the fluorinated layer is not necessarily more heterogeneous but rather possesses a lower effective infrared response. This behavior is consistent with weaker intermolecular interactions and a distinct molecular organization imposed by the electron-withdrawing terminal group.^45,46^ Therefore, the AFM-IR maps reveal differences in molecular organization and effective vibrational response imposed by the terminal-group electronics.

To further quantify the AFM-IR observations, statistical analysis of the pixel intensity distributions was performed for the characteristic phosphonate vibration at 1035 cm^–1^ and the aromatic triphenylamine vibration at 1467 cm^–1^ (Figure S38 and Table S2). For the 1035 cm^–1^ band, MeO-TPAP exhibited average intensities approximately two times higher than those of TPAP and F-TPAP. Similarly, for the 1467 cm^–1^ vibration, MeO-TPAP and TPAP displayed mean intensities approximately four times greater than F-TPAP. Analysis of the coefficient of variation (CV) revealed distinct spatial behaviors among the SAMs. MeO-TPAP combined high average intensity with comparatively low CV values, indicating a strong and spatially uniform response. In contrast, TPAP exhibited the largest fluctuations, suggesting increased nanoscale heterogeneity, whereas F-TPAP displayed a comparatively low average intensity accompanied by low CV values, indicative of a uniformly weak response.

X-ray photoelectron spectroscopy (XPS) measurements further confirmed the successful deposition of the TPA-based SAMs on ITO. Characteristic N 1s and P 2p signals were observed for all modified substrates, whereas F 1s was exclusively detected for F-TPAP (Figure S39). The similar P 2p line shapes indicate comparable phosphonate chemical environments at the ITO surface. Apparent thicknesses estimated from attenuation of the In 3d substrate signal using the approach proposed by Cumpson^47^ and effective attenuation lengths (EALs) yielded values between approximately 1.7 and 2.2 nm (Table S3), consistent with molecularly thin interfacial layers typically reported for TPA-based SAMs.^7,15,34^ Despite slight differences in apparent thickness, all three systems formed molecularly thin phosphonic acid layers. Combined with the AFM-IR results, these observations indicate that the distinct interfacial properties induced by the SAMs arise primarily from differences in molecular organization and effective vibrational response imposed by the terminal-group electronics. These differences establish the structural and chemical foundations that govern the interfacial energetics and charge-transfer properties discussed in the following sections.

The influence of the synthesized SAMs on the bulk crystal structure of two-step processed FAPbI_3_ perovskite films was investigated by X-ray diffraction (XRD), as shown in Figure 3a. Complementary measurements were also performed on the intermediate PbI_2_ layers deposited directly on the SAM-modified substrates (Figure S40). In both cases, no significant differences in peak intensity, position, or full-width at half-maximum (fwhm) were observed among the samples. These results indicate that while the terminal groups influence interfacial characteristics, they do not alter the bulk phase evolution or introduce impurity phases during the crystallization process.

**Figure 3:**
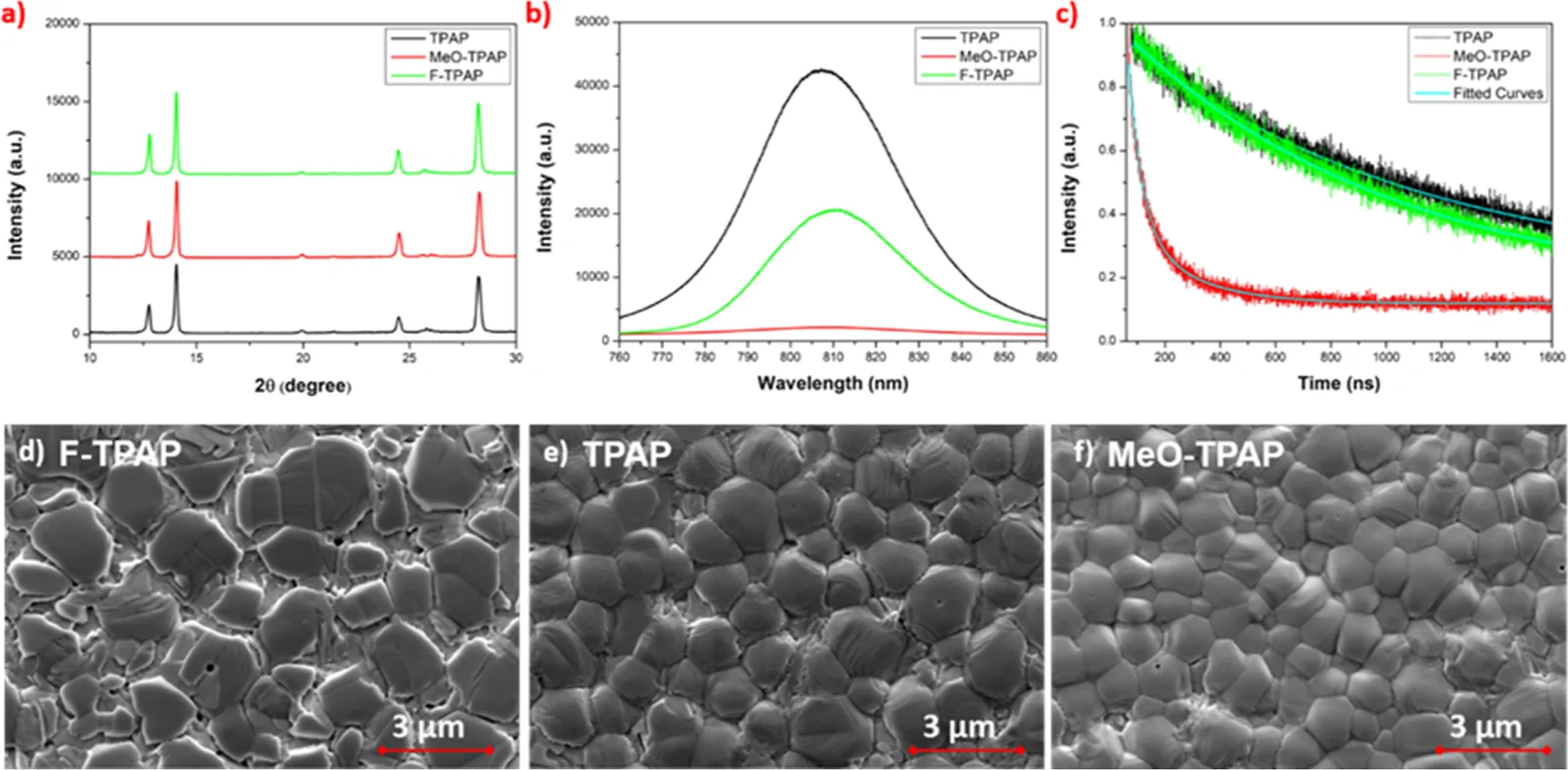
(**a**) X-ray diffraction (XRD) patterns of FAPbI_3_ films deposited on SAMs. (**b**) Steady-state photoluminescence (PL) spectra and (**c**) Time-resolved photoluminescence (TRPL) decay curves of FAPbI_3_ films deposited on SAMs. (**d–f**) Top-view SEM images of FAPbI_3_ films deposited on F-TPAP, TPAP, and MeO-TPAP, respectively.

To complement, the surface morphology of FAPbI_3_ films deposited on the different SAM-modified ITO substrates was evaluated by scanning electron microscopy (SEM) (Figure 3d–f). Although all samples exhibit crystalline features, consistent with the similar bulk crystallinity observed by XRD, pronounced differences in film microstructural quality are evident depending on the underlying SAM. In particular, the perovskite film grown on the F-TPAP exhibits significant intergranular voids and discontinuities, indicative of poor nucleation and agglomeration behavior.

In contrast, the perovskite layers deposited on TPAP and MeO-TPAP are compact, pinhole-free, and display well-connected grains. These morphological trends correlate with the nanoscale chemical organization observed by AFM-IR mapping, where MeO-TPAP exhibited a strong and spatially uniform infrared response, TPAP displayed increased nanoscale heterogeneity, and F-TPAP exhibited a uniformly weaker response. These observations suggest that the distinct molecular organizations revealed by AFM-IR correlate with different perovskite growth behaviors during the deposition process, leading to improved film quality. Quantitative grain size analysis (Figure S41 and Table S4) further supports these observations. Although F-TPAP exhibits the largest apparent average grain size (1.6 ± 0.5 μm), it displays the broadest grain size distribution and the lowest number of measurable grains (*N* = 213), indicating a morphology characterized by extended intergrain voids and poorly defined grain boundaries. In contrast, TPAP (1.4 ± 0.4 μm, *N* = 805) and particularly MeO-TPAP (1.1 ± 0.3 μm, *N* = 991) exhibit substantially larger populations of well-defined grains and narrower size distributions, consistent with a more homogeneous crystallization process. These results are in good agreement with the AFM-IR and XPS analyses, which revealed distinct molecular organizations despite comparable molecularly thin overlayers. The improved uniformity of the perovskite films formed on these SAMs is expected to reduce local interfacial mismatches and promote more efficient charge transfer across the SAM/perovskite interface.

Steady state photoluminescence (PL) measurements were employed to evaluate the radiative recombination behavior of FAPbI_3_ perovskite deposited over different synthesized SAMs. As shown in e Figure 3b, the perovskite films on TPAP and F-TPAP exhibit significantly higher PL intensities compared to the MeO-TPAP sample. It is important to note that the PL intensity is directly related to the radiative recombination rate, which scales with the product of the photogenerated electron and hole densities. Consequently, efficient interfacial charge extraction can reduce the PL intensity even when excited states are populated.^48^ The enhanced emission observed for TPAP and F-TPAP indicates a larger population of radiatively recombining charge carriers, suggesting comparatively weaker interfacial hole extraction given the reduced interfacial quenching under continuous illumination. This behavior aligns with the UPS results (Figure 1e), which indicated that the deep HOMO levels of these derivatives create an energetic barrier for hole injection. In contrast, the lower PL intensity for MeO-TPAP is attributed to more efficient interfacial hole extraction, which competes with radiative recombination. Considering that all samples were measured under identical excitation conditions, the improved energetic alignment at the MeO-TPAP/FAPbI_3_ interface promotes charge separation, thereby reducing the probability of radiative recombination.

In addition to the PL intensity variations, differences in the emission line width were also observed. As shown in Figure S42, the full width at half-maximum (fwhm) of the PL spectra decreased from 0.082 eV for F-TPAP to 0.077 eV for TPAP and 0.071 eV for MeO-TPAP. The narrower emission observed for MeO-TPAP indicates a more homogeneous electronic environment and reduced energetic disorder within the perovskite film. This trend is consistent with the AFM-IR results, which revealed a strong and spatially homogeneous infrared response for MeO-TPAP, indicating a more organized buried interface. Importantly, the reduced line width observed for MeO-TPAP suggests that the pronounced PL quenching is not associated with increased defect-assisted recombination, but rather with more efficient interfacial charge transfer enabled by the favorable energetic alignment and molecular organization at the SAM/FAPbI_3_ interface.

Time-resolved photoluminescence (TRPL) measurements were performed to investigate the carrier recombination dynamics at the SAM/FAPbI_3_ interface (Figure 3c). To account for the possible influence of interfacial and morphological heterogeneity, the decay profiles were analyzed using a stretched exponential model. The TPAP and F-TPAP samples were satisfactorily described by this approach, yielding β values of 0.782 and 0.856, respectively, which indicate moderate energetic disorder and the presence of a distribution of recombination environments. The corresponding characteristic lifetimes were approximately 1515 and 1179 ns, respectively (Table S5). These long-lived decays suggest relatively weak interfacial quenching, indicating that photogenerated carriers remain largely confined within the perovskite bulk and are unable to efficiently overcome the energetic barrier at the interface. This behavior is consistent with the unfavorable energetic alignment identified by UPS and confirms the limited charge extraction capability of these SAMs.

In contrast, the MeO-TPAP sample could not be adequately described by the stretched exponential model (*R* ^2^ = 0.79), indicating that the carrier dynamics cannot be represented by a single distributed recombination process. The superior agreement obtained with the biexponential fit (Figure S43, *R* ^2^ = 0.98) reveals the coexistence of two kinetically distinct carrier populations. The fast decay component (τ_1_ ≈ 87 ns) is consistent with efficient interfacial hole extraction and rapid depopulation of photogenerated carriers at the SAM/perovskite interface, whereas the slower component (τ_2_ ≈ 2658 ns) is associated with a minor population of carriers undergoing delayed recombination within the perovskite bulk.^42^ The markedly different decay behavior observed for MeO-TPAP highlights the more efficient charge transfer enabled by its distinct organization and favorable energy level alignment, ultimately suppressing long-lived radiative recombination. This conclusion is further supported by the reduced PL line width observed for MeO-TPAP (Figure S41), which indicates a narrower energetic distribution of emissive states and reduced energetic disorder. Together with the pronounced PL quenching, these results suggest that interfacial charge extraction, rather than defect-assisted nonradiative recombination, is the dominant origin of the observed PL intensity reduction and accelerated decay dynamics.

As summarized in the energy diagram shown in Figure 1e, the VBM of FAPbI_3_ is located at −5.8 eV, while the HOMO levels of TPAP and F-TPAP are positioned deeper, at −6.0 eV and −6.2 eV, respectively. This downward energetic offset may impose an additional barrier for hole transfer from the perovskite to the SAM layer, potentially limiting hole extraction at the interface. In contrast, MeO-TPAP exhibits a significantly shallower HOMO level at −5.4 eV, resulting in a more favorable energetic alignment with the perovskite valence band. This reduced energetic mismatch is expected to facilitate hole extraction from FAPbI_3_ into the SAM, consistent with the faster PL decay observed in the TRPL measurements.

To evaluate the practical implications of the distinct interfacial properties induced by the SAMs and to verify whether the trends observed for FAPbI_3_ films were preserved under optimized device conditions, inverted perovskite solar cells were fabricated using the synthesized monolayers as hole-selective contacts and a perovskite absorber prepared by a one-step process in a nitrogen-filled glovebox. Devices were prepared with an architecture of ITO/SAM/FA_0_._95_Cs_0_._05_PbI_3_/PCBM/BCP/Ag. Representative forward and reverse J–V curves of the champion devices are shown in Figure 4a, where solid and dashed lines correspond to reverse and forward scans, respectively. Statistical distributions of the photovoltaic parameters obtained from reverse scans are presented in Figure 4b–e, while the corresponding forward-scan distributions are shown in Figure S44. Complete average and champion photovoltaic parameters extracted from reverse and forward measurements are summarized in Tables S6 and S7, respectively.

**Figure 4:**
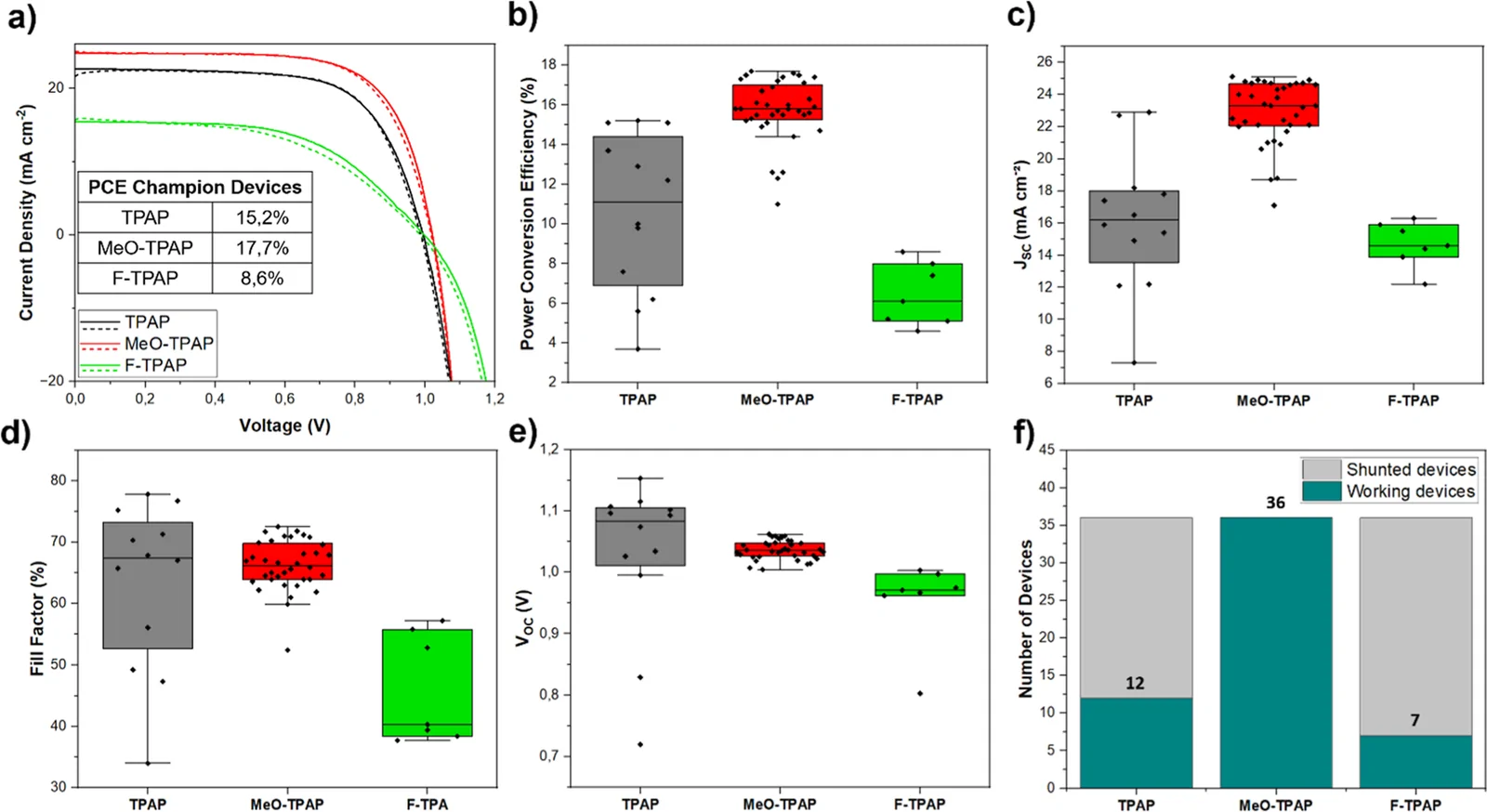
Photovoltaic performance and device statistics of inverted perovskite solar cells employing the synthesized SAMs as hole-selective contacts. (a) Representative current density–voltage (J–V) characteristics of the champion devices. Solid and dashed lines correspond to reverse and forward scans, respectively. Box plots showing the distributions of (b) power conversion efficiency (PCE), (c) short-circuit current density (*J*_sc_), (d) fill factor (FF), and (e) open-circuit voltage (*V*_oc_) obtained from reverse scans of functional devices. (f) Device yield represented by the number of functional and shunted cells among the 36 fabricated devices for each SAM.

Devices based on MeO-TPAP exhibited the highest average power conversion efficiency (15.7 ± 1.6%), with a champion efficiency of 17.7%, together with an average open-circuit voltage of 1.04 ± 0.01 V, a short-circuit current density of 22.9 ± 2.0 mA cm^–2^, and a fill factor of 66.2 ± 4.1%. In comparison, TPAP-based devices displayed a lower average PCE of 10.6 ± 4.1%, whereas F-TPAP yielded only 6.4 ± 1.6%. Interestingly, while MeO-TPAP and TPAP exhibit similar average *V*_OC_ and FF values, MeO-TPAP shows a considerably larger *J*_SC_, indicating that the performance enhancement is primarily associated with more efficient charge collection rather than reduced resistive losses. This observation is consistent with the stronger PL quenching and faster carrier extraction revealed by TRPL, suggesting that the favorable energetic alignment and improved interfacial organization provided by MeO-TPAP facilitate more efficient extraction of photogenerated holes and suppress carrier accumulation at the buried interface. Moreover, the compact and continuous perovskite morphology observed by SEM likely contributes to improved carrier transport and reduced recombination losses, leading to the significantly enhanced photocurrent.

The superior performance of MeO-TPAP is further consistent with the strong and spatially uniform AFM-IR response, favorable energetic alignment revealed by UPS, and accelerated carrier extraction evidenced by TRPL. In contrast, TPAP exhibited substantially larger parameter dispersion, particularly in *V*_OC_, *J*_SC_, and FF, reflecting the increased nanoscale heterogeneity observed by AFM-IR. Although F-TPAP displayed relatively narrow parameter distributions among functional devices, its performance was severely compromised and only 7 out of the 36 fabricated cells produced operational devices, whereas TPAP yielded 12 functional devices and MeO-TPAP exhibited a 100% device yield (Figure 4f). These trends are consistent with the SEM observations, which revealed compact and continuous perovskite films for MeO-TPAP and increasingly defective morphologies for TPAP and F-TPAP.

The hysteresis behavior was further evaluated by calculating the hysteresis index (HI) for all functional devices. The corresponding statistical distributions are presented in Figure S45, while the median HI values and associated interquartile ranges are summarized in Table S8. Overall, all devices exhibited relatively small hysteresis, with MeO-TPAP showing the lowest scan-direction dependence.

## Conclusions

Overall, the present results demonstrate that the electronic nature of the terminal groups in TPA-based SAM hole-selective interfaces governs both molecular organization and interfacial energetics, thereby influencing perovskite film formation and charge extraction. Recent studies have reported that the introduction of a small fraction of methoxy-functionalized SAMs into TPAP-based layers primarily enhances perovskite wettability.^18^ While improved surface wetting undoubtedly contributes to better morphological control, our results demonstrate that terminal-group engineering also plays a critical role in defining the electronic properties and molecular organization of the interface. In particular, the shallower HOMO level of MeO-TPAP results in a smaller energetic offset with respect to the perovskite valence band, suggesting a reduced energetic barrier for hole extraction. This interpretation is further supported by the PL, TRPL, and photovoltaic measurements. These findings indicate that the electronic nature of the terminal groups governs not only the molecular organization and wetting properties of the SAM, but also modulates interfacial energetics and charge-transfer processes. Overall, the synergy between molecular organization and interfacial energetics dictates perovskite film quality, charge extraction efficiency, and ultimately device reproducibility and fabrication yield. These results highlight molecular organization as a key design parameter for the development of efficient and reliable SAM-based interfaces for perovskite solar cells.

## Experimental Section

### Materials and Self-Assembled Monolayers Synthesis

All reagents and solvents were purchased from commercial suppliers and used as received unless otherwise stated. The synthesis of the triphenylamine-based phosphonic acid derivatives (F-TPAP, TPAP, and MeO-TPAP) was carried out following the procedures described in the Supporting Information. Structural characterization was performed by ^1^H, ^13^C, ^31^P, and ^19^F NMR spectroscopy, HRMS, and FTIR spectroscopy.

### Thin Film Preparation

ITO substrates were sequentially cleaned by ultrasonication in acetone and isopropanol, followed by oxygen plasma treatment. The SAMs were deposited by spin coating 1 mM ethanolic solutions, followed by thermal annealing. Detailed deposition conditions are provided in the Supporting Information. FAPbI_3_ perovskite films employed for interfacial characterization were prepared by a two-step deposition process under ambient conditions. PbI_2_ was first spin-coated onto the SAM-modified substrates, followed by conversion using an organic-halide solution. Complete preparation conditions are described in the Supporting Information.

### Characterization

The SAMs and interfaces were characterized by FTIR, XPS, AFM-IR, UPS, contact-angle measurements, SEM, steady-state photoluminescence (PL), and time-resolved photoluminescence (TRPL). Detailed instrumental parameters and data analysis procedures are provided in the Supporting Information.

### Device Fabrication and Characterization

To evaluate the practical implications of the interfacial properties, inverted perovskite solar cells were fabricated employing the synthesized SAMs as hole-selective contacts. Devices were prepared using an optimized one-step FA_0.95_Cs_0.05_PbI_3_ absorber under nitrogen atmosphere. Current density–voltage (J–V) measurements were performed under simulated AM 1.5G illumination (100 mW cm^–2^). Complete fabrication procedures and measurement conditions are described in the Supporting Information.

## Supplementary Material



## Notes

Materials and methods, including detailed synthetic procedures for all compounds and intermediates; nuclear magnetic resonance (^1^H, ^13^C, ^31^P, and ^19^F NMR) spectra and high-resolution mass spectrometry (HRMS) data for all final compounds and intermediates; procedures for self-assembled monolayer (SAM) preparation and perovskite film fabrication; and detailed descriptions of all characterization methods. Supplementary spectroscopic and structural data, including UV–vis absorption spectra and Tauc plot analysis, AFM-IR measurements, scanning electron microscopy (SEM) images, X-ray and ultraviolet photoelectron spectroscopy (XPS/UPS) spectra, X-ray diffraction (XRD) patterns, and contact angle measurements. Supplementary tables containing time-resolved photoluminescence (TRPL) parameters, optical bandgap values extracted from Tauc plots, and additional fitting and statistical data supporting the main text (PDF)

## Abbreviations

AFM-IR: atomic force microscopy–infrared spectroscopy
DMF: *N*,*N*-dimethylformamide
DMSO: dimethyl sulfoxide
*E*_g_: optical bandgap
FT-IR: Fourier-transform infrared spectroscopy
fwhm: full width at half-maximum
HOMO: highest occupied molecular orbital
HTM: hole-transporting material
iPSC: inverted perovskite solar cell
ITO: indium tin oxide
PCE: power conversion efficiency
PL: photoluminescence
RMS: root-mean-square
SAM: self-assembled monolayer
SEM: scanning electron microscopy
TPA: triphenylamine
TRPL: time-resolved photoluminescence
TCO: transparent conductive oxide
UPS: ultraviolet photoelectron spectroscopy
VBM: valence band maximum
UV–Vis: ultraviolet–visible spectroscopy
XPS: X-ray photoelectron spectroscopy
XRD: X-ray diffraction
