# Supplementary material for: Terminal Group Effects on Molecular Organization and Charge Extraction in Self-Assembled Monolayers/Perovskite Interfaces
Source: ACS Mater Au. 2026 Jul 16;6(5):1121–30. doi: 10.1021/acsmaterialsau.6c00070 (PMC13564142; doi:10.1021/acsmaterialsau.6c00070)
Supplement: Supplementary file 1 [file mg6c00070_si_001.pdf]

Supporting Information

TERMINAL GROUP EFFECTS ON MOLECULAR ORGANIZATION AND CHARGE EXTRACTION IN SAM/PEROVSKITE INTERFACES

Arthur P. Machado[1], Felipe C. Braga[1], Carlos H. Stadtlober[1], Paulo E. Marchezi[1], Douglas S. Ribeiro[2], Murillo H. M. Rodrigues[1], Zeno C. Brandão[2], Diogo M. G. Neto[1], Caio C. Oliveira[1*], Ana F. Nogueira[1]*

[1]Department of Chemistry, Institute of Chemistry, University of Campinas (UNICAMP), Campinas, SP, 13083-970, Brazil

[2]Institute of Physics Gleb Wataghin, University of Campinas (UNICAMP), Campinas, SP 13083-859, Brazil

Corresponding author:

*E-mail: anafla@unicamp.br

MATERIALS

SYNTHETIC PROCEDURES

SOLUTIONS, THIN FILM AND DEVICE PREPARATION

CHARACTERIZATION METHODS

SUPPLEMENTARY FIGURES

SUPPLEMENTARY TABLES

SUPPLEMENTARY REFERENCES

MATERIALS:

All reactions were carried out using anhydrous solvents under a nitrogen atmosphere. Dry toluene was obtained from an MBraun SPS 5/7 solvent purification system. Hexane was purchased from Synth and purified by fractional distillation. Methanol, dichloromethane, toluene, and 1,4-dioxane (anhydrous, in sealed bottles) were purchased from Synth and Neon and used without further purification. 4-iodoanisole, iodobenzene, 4-bromoaniline, 1,10-phenanthroline, potassium hydroxide, diethyl phosphite, palladium acetate, xantphos, and bromotrimethylsilane were purchased from Merck–Sigma-Aldrich and used as received. Copper(I) chloride was purified by dissolution in 12 M HCl followed by dilution with distilled water, yielding a white powder that was decanted and, after removal of the supernatant, dried under high vacuum and stored in a glovebox. Triethylamine was distilled and stored over potassium hydroxide pellets under refrigeration.

Indium tin oxide (ITO, 7 Ω sq^{-1}) glass substrates were purchased from Kintec Company. Anhydrous solvents, including dimethylformamide (DMF), dimethyl sulfoxide (DMSO), isopropyl alcohol (IPA), chloroform and ethanol (EtOH), were purchased from Merck–Sigma-Aldrich. Formamidinium iodide (FAI) was obtained from Great Cell Solar Ltd. Lead iodide (PbI_2, >99%), PCBM and bathocuproine (BCP) were purchased from Tokyo Chemical Industry (TCI) America. Methylammonium Chloride (MACl) was synthesized and purified following the procedure reported in.[S1] All materials were used as received without further purification.

SYNTHETIC PROCEDURES:

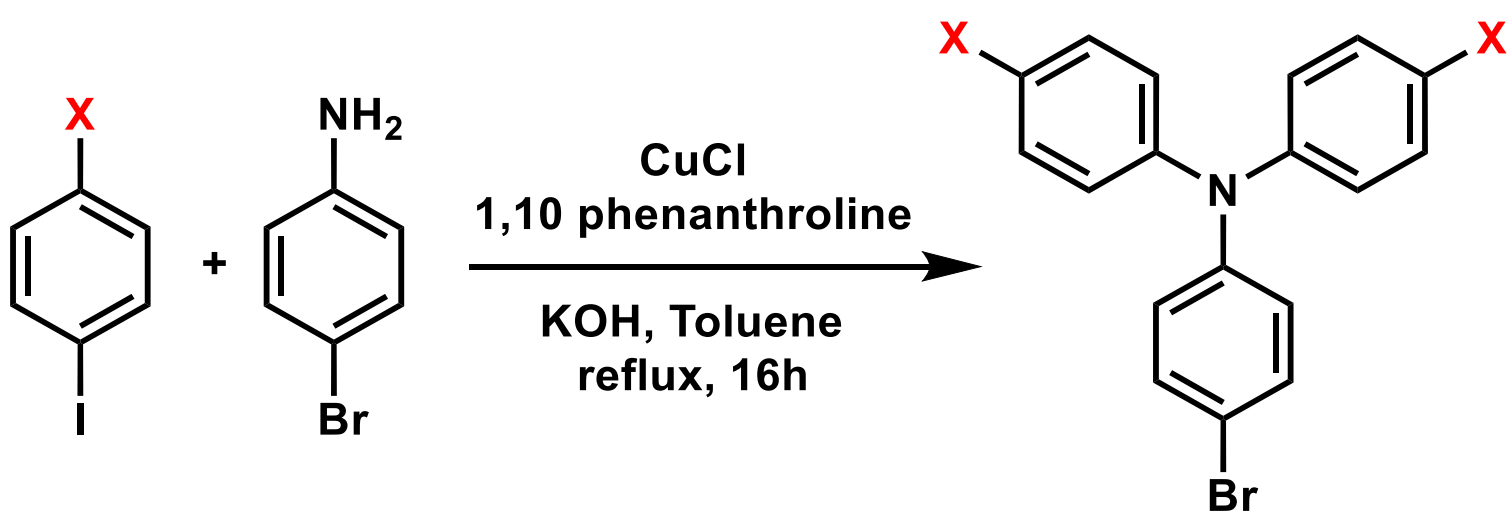

X = -H, -OMe and -F

(1-3)

Figure S1. General procedure for the synthesis of triphenylamine bromides **(1-3)**.

General procedure for the synthesis of 1-3: To a solution of toluene (100 mL) in a round-bottom flask equipped with a Dean-Stark trap and a reflux condenser, were added 4-bromoaniline (4.0 g, 23.25 mmol), the corresponding aryl halide (51.16 mmol, 2.2 equiv.), CuCl (460 mg, 4.65 mmol, 20 mol%), 1,10-phenanthroline (838 mg, 4.65 mmol, 20 mol%), and KOH (10.4 g, 186 mmol, 8.0 equivalents). The resulting mixture was stirred at reflux for 16 h. After completion of the reaction, the solvent was removed under reduced pressure, and the residue was diluted with dichloromethane (300 mL) and washed with distilled water (3 × 100 mL). The organic layer was dried over anhydrous sodium sulfate, filtered, and concentrated under reduced pressure. The crude product was purified by column chromatography on silica gel using a mixture of dichloromethane and hexane (3:7) as eluent to afford the pure products.

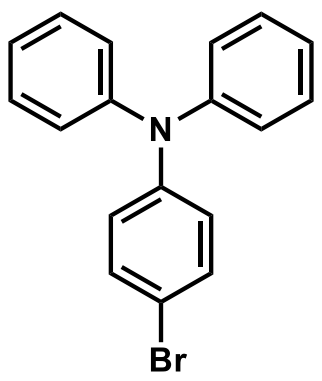

4-Bromo-*N,N*-diphenylaniline (1): White solid (4.39 g, 58 % yield). TLC (R_f = 0.64 in Silica gel: DCM:hexanes 3:7, stains with vanillin resulting a purple spot, UV active). **^{1}H NMR** (300 MHz, $CDCl_3$) δ 7.30 (d, *J* = 8.8 Hz, 2H), 7.27 – 7.19 (m, 4H), 7.09 – 6.98 (m, 6H), 6.93 (d, *J* = 8.8 Hz, 2H). **^{13}C NMR** (75 MHz, $CDCl_3$) δ 147.4, 147.1, 132.2, 129.4, 125.2, 124.5, 123.3, 114.8. **(Figure S4-5)**

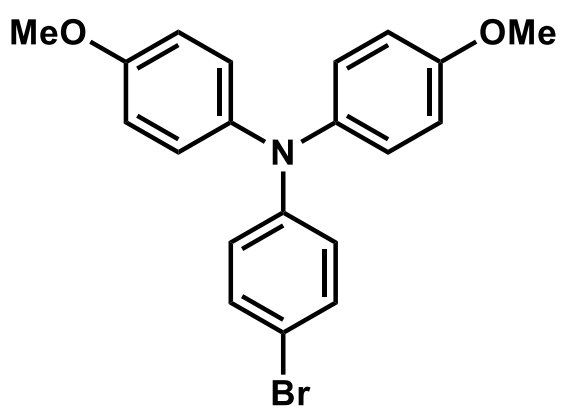

4-bromo-*N,N*-bis(4-methoxyphenyl)aniline (2): Orange solid (1.37 g, 61% yield, from 5.81 mmol of starting material). TLC (R_f = 0.36 in Silica gel: DCM:hexanes 3:7, stains with vanillin resulting a blue spot, UV active). **^{1}H NMR** (500 MHz, $CDCl_3$) δ 7.26 (d, *J* = 8.9 Hz, 2H), 7.05 (d, *J* = 8.9 Hz, 4H), 6.85 (d, *J* = 8.9 Hz, 4H), 6.82 (d, *J* = 8.9 Hz, 2H), 3.82 (s, 6H). **^{13}C NMR** (126 MHz, $CDCl_3$) δ 156.1, 147.9, 140.6, 131.8, 127.7, 126.6, 122.0, 114.8, 114.2, 112.4, 55.5. **(Figure S6-7)**

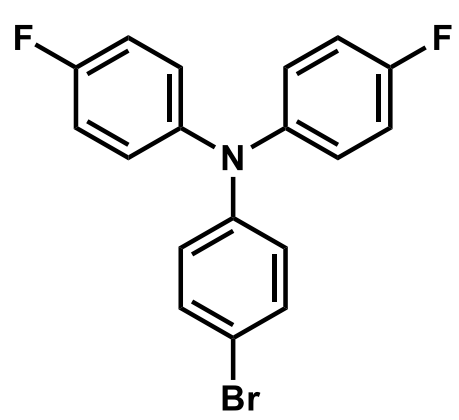

4-bromo-*N,N*-bis(4-fluorophenyl)aniline (3): White solid (1.44 g, 50% yield, from 8.19 mmol of starting material). TLC (R_f = 0.60 in Silica gel: DCM:hexanes 2:8, UV active). **^{1}H NMR** (600 MHz, $CDCl_3$) δ 7.31 – 7.27 (m, 2H), 7.04 – 6.98 (m, 4H), 6.97 – 6.92 (m, 4H), 6.86 – 6.82 (m, 2H). **^{13}C NMR** (151 MHz, $CDCl_3$) δ 159.9, 158.3, 147.2, 143.4, 143.4, 132.2, 126.2, 126.2, 123.9, 116.4, 116.3, 114.4. **^{19}F NMR** (470 MHz, $CDCl_3$) δ -118.94. **(Figure S8-10)**

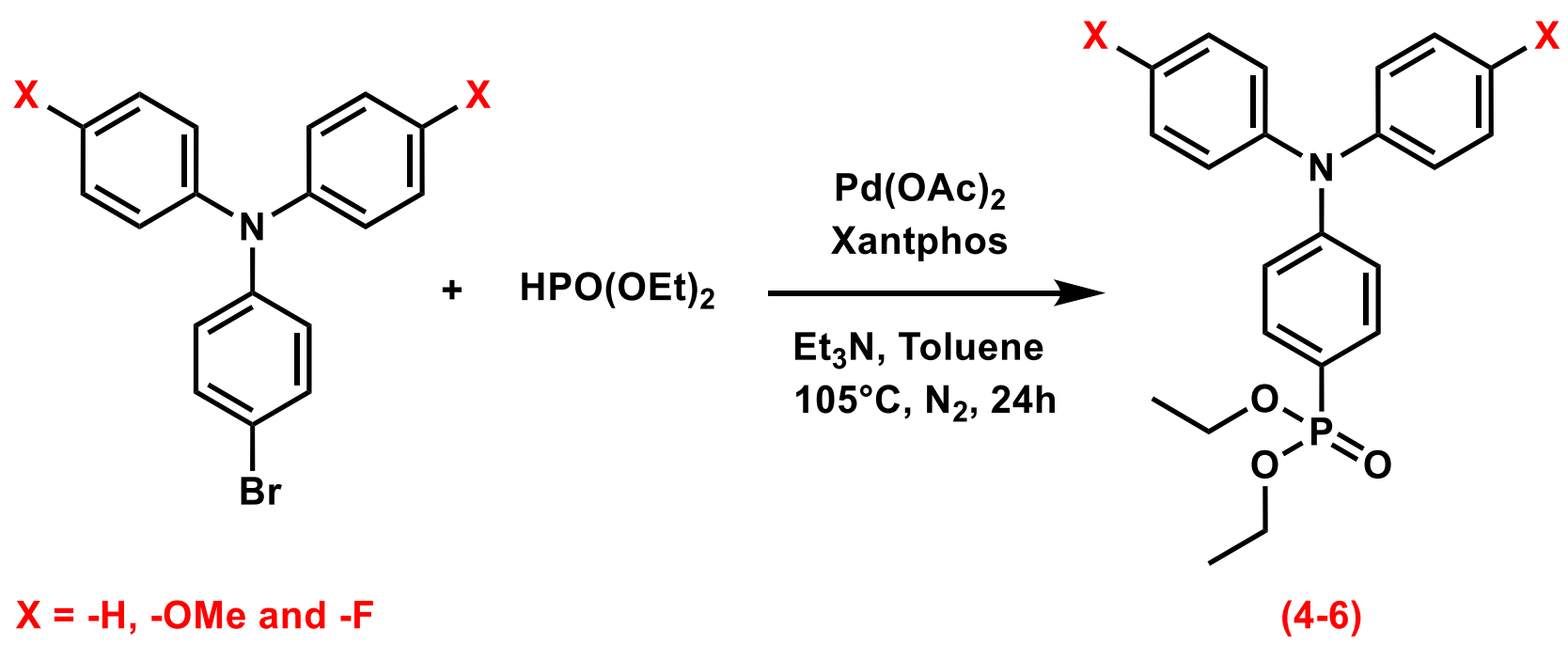

Figure S2. General procedure for the synthesis of phosphonates **(4-6).**

General procedure for the synthesis of 4-6: A flame-dried 250 mL round-bottom flask was charged with palladium acetate (28 mg, 0.125 mmol, 5 mol%), Xantphos (80 mg, 0.135 mmol, 5.5 mol%), and the corresponding triphenylamine bromide (2.5 mmol, 1.0 equivalent). Dried and degassed toluene (10 mL) was added, and the resulting mixture was stirred. Diethyl phosphite (322 µL, 2.5 mmol, 1.0 equivalent) was added via syringe, followed by dropwise addition of triethylamine (453µL, 3.25 mmol, 1.3 equivalent). The reaction mixture was stirred at 105 °C for 24 h under an inert atmosphere. After completion of the reaction, the solvent was removed under reduced pressure, and the residue was purified by column chromatography on silica gel using dichloromethane:methanol (9.8:0.2) as eluent to afford the pure product as an oil.

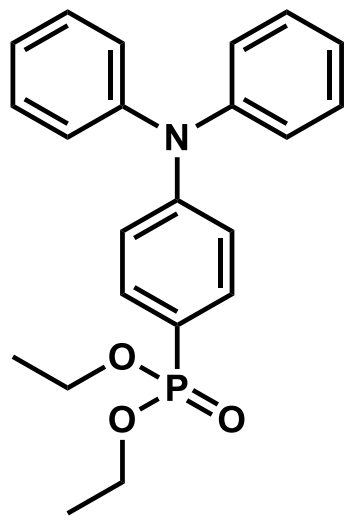

diethyl (4-(diphenylamino)phenyl)phosphonate (4): Redish liquid (543 mg, 57 % yield). TLC (R_f = 0.52 in Silica gel: DCM:methanol 9.8:0.2, stains with vanillin resulting in a bluish spot, UV active). δ ^{1}**H NMR** (500 MHz, $CDCl_3$) δ 7.59 (dd, *J* = 12.7, 8.7 Hz, 2H), 7.30 (dd, *J* = 8.6, 7.3 Hz, 4H), 7.16 – 7.08 (m, 6H), 7.02 (dd, *J* = 8.7, 3.5 Hz, 2H), 4.20 – 4.00 (m, 4H), 1.33 (t, *J* = 7.0 Hz, 6H). ^{13}**C NMR** (126 MHz, $CDCl_3$) δ 151.5, 151.5, 146.6, 133.0, 132.9, 129.6, 125.8, 124.4, 120.3, 120.2, 119.7, 118.2, 61.9, 61.9, 16.4, 16.3. ^{31}**P NMR** (202 MHz, $CDCl_3$) δ 20.05. **(Figure S11-13)**

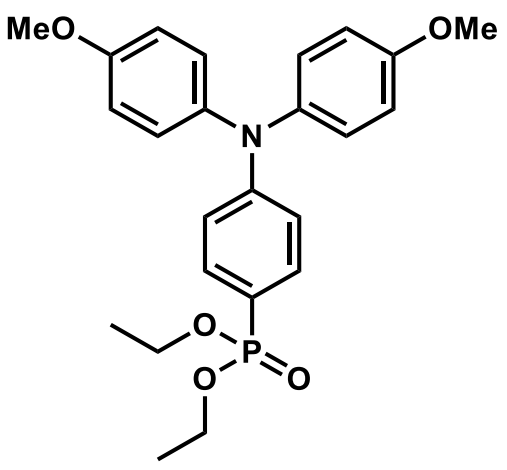

diethyl (4-(bis(4-methoxyphenyl)amino)phenyl)phosphonate (5): Dark yellow liquid (395 mg, 56 % yield, from 1.61 mmol of starting material). TLC (R_f = 0.3 in Silica gel: DCM:methanol 9.8:0.2, stains with vanillin resulting in a bluish spot, UV active). ^{1}**H NMR** (500 MHz, $CDCl_3$) δ 7.53 (dd, *J* = 12.7, 8.8 Hz, 2H), 7.10 (d, *J* = 8.9 Hz, 4H), 6.90 – 6.82 (m, 6H), 4.19 – 3.98 (m, 4H), 3.81 (s, 6H), 1.31 (t, *J* = 7.0 Hz, 6H). ^{13}**C NMR** (126 MHz, $CDCl_3$) δ 156.9, 152.3, 152.2, 139.4, 133.0, 132.9, 127.8, 117.4, 117.2, 114.9, 61.8, 61.7, 55.5, 16.4, 16.3. ^{31}**P NMR** (202 MHz, $CDCl_3$) δ 20.71. **(Figure S14-16)**

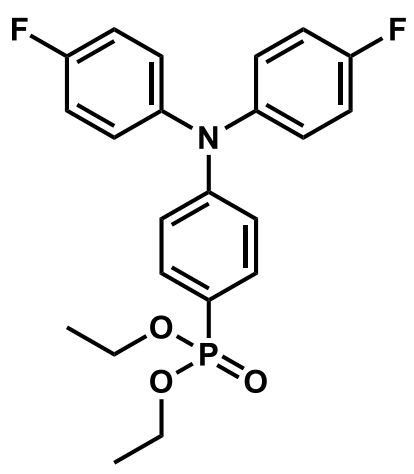

diethyl (4-(bis(4-fluorophenyl)amino)phenyl)phosphonate (6): Dark red liquid (651 mg, 51 % yield, from 3.05 mmol of starting material). TLC (R_f = 0.33 in Silica gel: DCM:methanol 9.8:0.2, stains with vanillin, UV active). ^{1}**H NMR** (500 MHz, $CDCl_3$) δ 7.61 (dd, *J* = 12.6, 8.7 Hz, 2H), 7.17 – 7.09 (m, 4H), 7.08 – 7.01 (m, 4H), 6.94 (dd, *J* = 8.7, 3.5 Hz, 2H), 4.23 – 4.03 (m, 4H), 1.35 (t, *J* = 7.1 Hz, 6H). ^{13}**C NMR** (126 MHz, $CDCl_3$) δ 160.76, 158.81, 151.53, 151.50, 142.49, 142.47, 133.13, 133.04, 127.61, 127.55, 119.75, 119.16, 119.04, 118.19, 116.69, 116.51, 61.94, 61.90, 16.41, 16.36. ^{31}**P** NMR (202 MHz, $CDCl_3$) δ 19.82. ^{19}**F NMR** (470 MHz, $CDCl_3$) δ -117.24. **(Figure S17-20)**

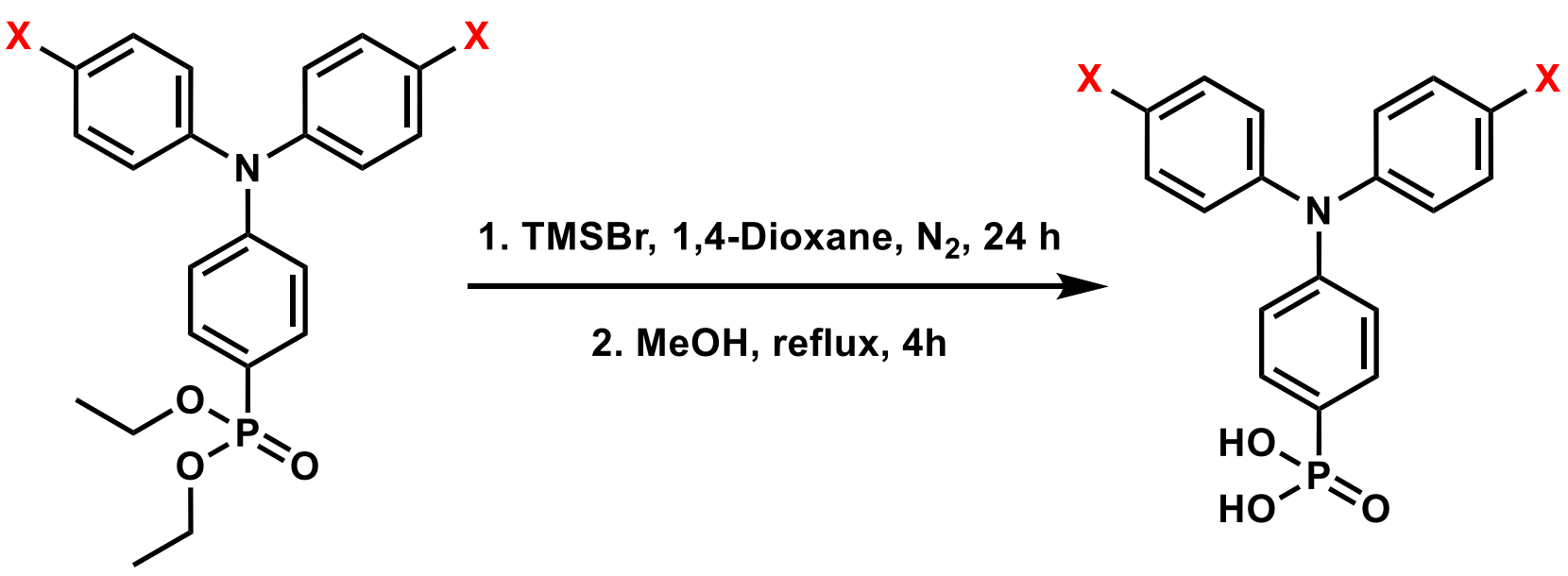

X = -H, -OMe and -F

(7-9)

Figure S3. General procedure for the synthesis of phosphonic acid final products (7-9).

General procedure for the synthesis of 7-9: To a 250 mL round-bottom flask was transferred the corresponding phosphonate (377 mg, 0.99 mmol) and the oil was dried under high vacuum for 1 h. The flask was then filled with nitrogen and sealed with

a septum. The residue was dissolved in dry 1,4-dioxane (5 mL), and bromotrimethylsilane (287 µL, 2.18 mmol, 2.2 equivalent) was added dropwise to the well-stirred mixture at room temperature. After stirring for 24 h, the mixture was concentrated under reduced pressure. Methanol (100 mL) was then added, and the reaction mixture was stirred at 65 °C (reflux) for 4 h. The solvent was partially removed under reduced pressure, and the resulting solution was cooled to precipitate impurities. After filtration, the solvent was removed under reduced pressure, and the crude product was precipitated from a mixture of DCM and hexane (3:7). The pure product was isolated by filtration and dried under vacuum.

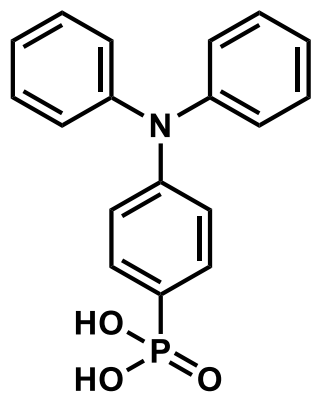

(4-(diphenylamino)phenyl)phosphonic acid (7): White powder (210 mg, 65 % yield). **^{1}H NMR** (500 MHz, DMSO-d_6) δ 7.54 (dd, J = 12.6, 8.6 Hz, 2H), 7.35 (dd, J = 8.4, 7.4 Hz, 4H), 7.12 (t, J = 7.4 Hz, 2H), 7.07 (d, J = 7.2 Hz, 4H), 6.95 (dd, J = 8.6, 2.9 Hz, 2H). **^{13}C NMR** (126 MHz, DMSO-d_6) δ 150.1, 150.1, 147.0, 132.5, 132.4, 130.2, 127.4, 125.9, 125.5, 124.5, 121.1, 121.0. **^{31}P NMR** (121 MHz, DMSO-d_6) δ 13.39. **HRMS** for expected ion [$C_{18}H_{16}NO_3P$ + H]: 326.0941; found: 326.0935, error of 1.8 ppm. **(Figure S21-24)**

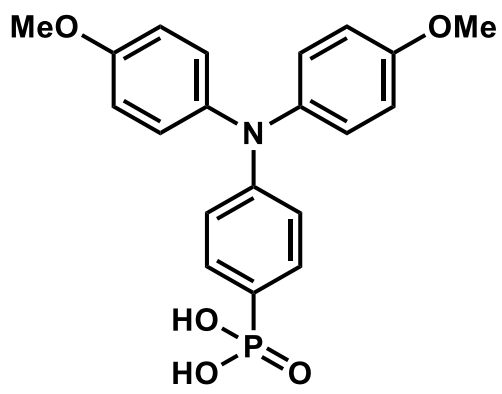

(4-(bis(4-methoxyphenyl)amino)phenyl)phosphonic acid (8): White powder (160 mg, 47 % yield, from 0.88 mmol of starting material). **^{1}H NMR** (500 MHz, DMSO-d_6) δ 7.44 (dd, J = 12.5, 8.7 Hz, 2H), 7.08 (d, J = 8.9 Hz, 4H), 6.94 (d, J = 9.0 Hz, 4H), 6.72 (dd, J = 8.8, 2.9 Hz, 2H). **^{13}C NMR** (126 MHz, DMSO-d_6) δ 156.8, 151.1, 151.1, 139.7, 132.3, 132.2, 128.0, 124.5, 123.0, 117.3, 117.2, 115.6, 55.7. **^{31}P NMR** (121 MHz, DMSO-d_6) δ 14.17. **HRMS** for expected ion [$C_{20}H_{20}NO_5P$ + H]: 386.1152; found: 386.1139, error of 3.4 ppm and for [$C_{20}H_{20}NO_5P$]: 385.1079; found: 385.1072, error of 1.8 ppm. **(Figure S25-28)**

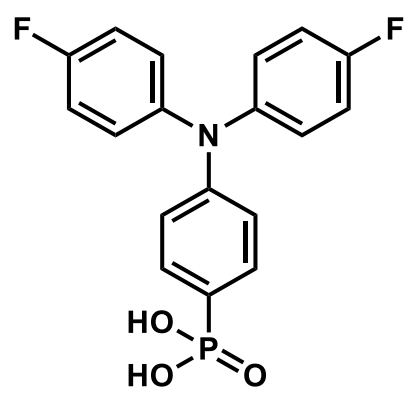

(4-(bis(4-fluorophenyl)amino)phenyl)phosphonic acid (9): White powder (461 mg, 82 % yield, from 1.56 mmol of starting material). **^{1}H NMR** (500 MHz, DMSO-d_6) δ 7.52 (dd, J = 12.5, 8.6 Hz, 2H), 7.24 – 7.18 (m, 4H), 7.16 – 7.10 (m, 4H), 6.86 (dd, J = 8.7, 2.9 Hz, 2H). **^{13}C NMR** (126 MHz, DMSO-d_6) δ 160.3, 158.4, 150.3, 150.2, 143.2, 143.2, 132.5, 132.5, 131.3, 131.0, 130.9, 128.6, 128.5, 127.9, 127.8, 126.8, 125.4, 119.7, 119.5, 117.2, 117.0, 55.4. **^{31}P NMR** (202 MHz, DMSO-d_6) δ 13.46. **^{19}F NMR** (470 MHz, DMSO-d_6) δ -117.96. **HRMS** for expected ion [$C_{18}H_{15}F_2NO_3P$ + H]: 362.0758; found: 362.0747, error of 3.4 ppm and for [$C_{18}H_{15}F_2NO_3P$+Na]: 384.0577; found: 384.0568, error of 2.3 ppm. **(Figure S29-33)**

SOLUTIONS, THIN FILM AND DEVICE PREPARATION:

Self-assembled monolayer (SAM) solutions were prepared by dissolving the corresponding molecules in anhydrous ethanol at a concentration of 1 mM. The PbI_2 precursor solution was prepared by dissolving lead iodide (PbI_2) in a mixed solvent of anhydrous N,N-dimethylformamide (DMF) and dimethyl sulfoxide (DMSO) with a 9:1 volume ratio, yielding a final concentration of 1.5 M. The solution was stirred for 2 h and subsequently filtered through a 0.22 μm PTFE syringe filter prior to deposition. The formamidinium iodide (FAI) solution was prepared by dissolving 90 mg of FAI and 15 mg of methylammonium chloride (MACl) in anhydrous isopropanol (IPA). This solution was also stirred for 2 h and filtered using a 0.22 μm PTFE filter before use.

Indium tin oxide (ITO) coated glass substrates (1 × 1 cm^2) were sequentially cleaned by ultrasonication in acetone for 20 min, followed by isopropanol (IPA) for 20 min. After drying, the substrates were treated with oxygen plasma for 10 min to improve surface cleanliness and wettability. SAM layers were deposited by spin coating at 3000 rpm with an acceleration of 500 rpm s^{-1} for 30 s, followed by thermal annealing at 100 °C for 15 min. The PbI_2 solution was spin-coated onto the SAM-modified substrates at 1500 rpm for 30 s with an acceleration of 500 rpm s^{-1}, followed by annealing at 70 °C for 1 min. After cooling to room temperature, the FAI solution was deposited by dynamic spin coating onto the PbI_2 film at 1800 rpm for 30 s, immediately after dispensing the solution. The resulting films were then annealed at 150 °C for 15 min to complete the perovskite formation. All depositions were carried out with controlled humidity of 25~30%.

Inverted perovskite solar cells were fabricated on 1.5 × 2.5 cm^2 ITO-coated glass substrates modified with the synthesized self-assembled monolayers (SAMs). Prior to deposition, the substrates were cleaned following the procedure described above. The hole-selective layers were prepared by spin-coating 1 mM solutions of the corresponding SAMs in absolute ethanol, followed by annealing at 100 °C for 10 min. The perovskite absorber consisted of $FA_{0.95}Cs_{0.05}PbI_3$ containing 30 mol% MACl additive. A 1.2 M precursor solution was deposited by a one-step spin-coating process at 4000 rpm inside a nitrogen-filled glovebox. Chlorobenzene was dynamically dropped 10 s before the end of the spin-coating process as an antisolvent. The films were subsequently annealed at 100 °C for 30 min. The electron transport layer was formed by spin-coating a 15 mg mL^{-1} PCBM solution in chloroform at 2000 rpm, followed by deposition of a 0.5 mg mL^{-1} BCP solution in isopropanol at 5000 rpm. Finally, a 100 nm thick silver electrode was thermally evaporated, yielding devices with an architecture of: ITO / SAM / $FA_{0.95}Cs_{0.05}PbI_3$ / PCBM / BCP / Ag. The active area, defined by a shadow mask, was 0.16 cm^2. Current density–voltage (J–V) characteristics were measured under simulated AM 1.5G illumination (100 mW cm^{-2}) at a scan rate of 100 mV s^{-1}. Reverse and forward scans were performed from 1.2 to −0.1 V and from −0.1 to 1.2 V, respectively. Statistical analyses were carried out considering only functional devices.

CHARACTERIZATION METHODS:

Thin-layer chromatography (TLC) was performed on silica gel 60 F254 plates (Merck, 0.25 mm thickness) and analyzed under UV light (254 nm) and with appropriate staining reagents as indicated. Flash chromatography was carried out using glass columns packed with silica gel 60 (Aldrich, 40–63 µm).

^1H, ^{13}C, ^{31}P, and ^{19}F Nuclear magnetic resonance (NMR) spectra were recorded at 25 °C on Bruker AVANCE II (300 MHz) and AVANCE III (500 MHz) spectrometers in $CDCl_3$ and DMSO-d_6, using residual $CHCl_3$ (^1H = 7.26 ppm; ^{13}C = 77.16 ppm) or CD_2HSOCD_3 (^1H = 2.50 ppm; ^{13}C = 39.52 ppm) and TMS (^1H = 0 ppm) as internal standards. Chemical shifts are reported in parts per million (ppm). Data are presented as follows: chemical shift, multiplicity (s = singlet, bs = broad singlet, d = doublet, dd = doublet of doublets, ddd = doublet of doublets of doublets, t = triplet, td = triplet of doublets, q = quartet, sept = septet, m = multiplet), coupling constant (when applicable), and integration.

High-resolution mass spectrometry (HRMS) analyses, samples were diluted to a concentration of 1 ppm in MeOH:H_2O and analyzed using an ultra-high-performance liquid chromatograph (UHPLC) for mass spectral acquisition. An isocratic mobile phase composed of water (solvent A, with 0.1% formic acid) and methanol (solvent C, with 0.1% formic acid) in a 1:1 v/v ratio was employed. The mobile phase flow rate was 0.200 mL min^{-1}, and the injection volume was 20 µL. Quantitative analyses were performed on a Thermo Q Exactive Orbitrap mass spectrometer equipped with an electrospray ionization (ESI) interface operating in positive mode.

UV-visible absorption (UV-Vis) spectra of the SAM thin films deposited over quartz were acquired using an Agilent spectrophotometer, model Cary 60.

Fourier-Transform Infrared Spectroscopy (FT-IR) were recorded in transmission mode using an Agilent Cary 630 spectrometer over the 4000–400 cm^{-1} range, with a spectral resolution of 4 cm^{-1} and 64 co-added scans. A pure KBr pellet was used as background. Baseline correction was applied prior to analysis. Powder samples of SAM molecules were finely ground and mixed with KBr (≈1–2 wt%) using an agate mortar and pestle. The mixture was pressed into a transparent pellet.

Contact angle measurements were realized using a Reme-Hart 100-00-115 goniometer by depositing 2 µL droplets of the respective solvents onto the sample surface at room temperature.

Ultraviolet and X-ray Photoemission Spectroscopy (XPS and UPS) measurements were carried out with a SPECS Phoibos 150 Hemispherical analyzer. A He I lamp (21.2 eV) radiation source was used for UPS and a monochromatized Al Kα radiation source (1486.71 eV) was used for XPS measurements.

Atomic force microscopy coupled with infrared spectroscopy (AFM-IR) measurements were performed in a Bruker® nanoIR2-s atomic force microscope. Topography images were acquired in contact mode using a BudgetSensors® ContGB-G probe, with a nominal spring constant of 0.2 N/m and a tip end radius of <25.0 nm. Infrared spectra were obtained using a Daylight Solutions® MIRcat-QT-2400 quantum cascade laser.

X-ray diffraction (XRD) patterns were recorded using a Shimadzu diffractometer with Cu Kα radiation ($\lambda = 1.5406$ Å), operating at 40 kV and 30 mA, in Bragg–Brentano geometry with a θ–2θ scan at room temperature.

Steady-state photoluminescence (PL) and time-resolved photoluminescence (TRPL) measurements were performed using a Horiba Fluorolog-3 spectrometer. The PL spectra were acquired under excitation at 485 nm. TRPL measurements were carried out using the same excitation wavelength (485 nm), with the emission monitored at 804 nm, corresponding to the main radiative recombination peak of the samples. The TRPL decay curves were analyzed using mono- or bi-exponential fitting, depending of the sample, and the effective lifetime for bi-exponential decays was obtained from the amplitude-weighted average of the individual decay components. $\tau_{avg} = (A_1\tau_1 + A_2\tau_2)/(A_1 + A_2)$, where A_i and τ_i are the amplitudes and lifetimes of each decay component.

Scanning electron microscopy (SEM) images were obtained using a FEI Quanta FEG 250 microscope operating in high-vacuum mode with a field-emission gun, using secondary electron detection at an accelerating voltage of 5 kV.

SUPPLEMENTARY FIGURES:

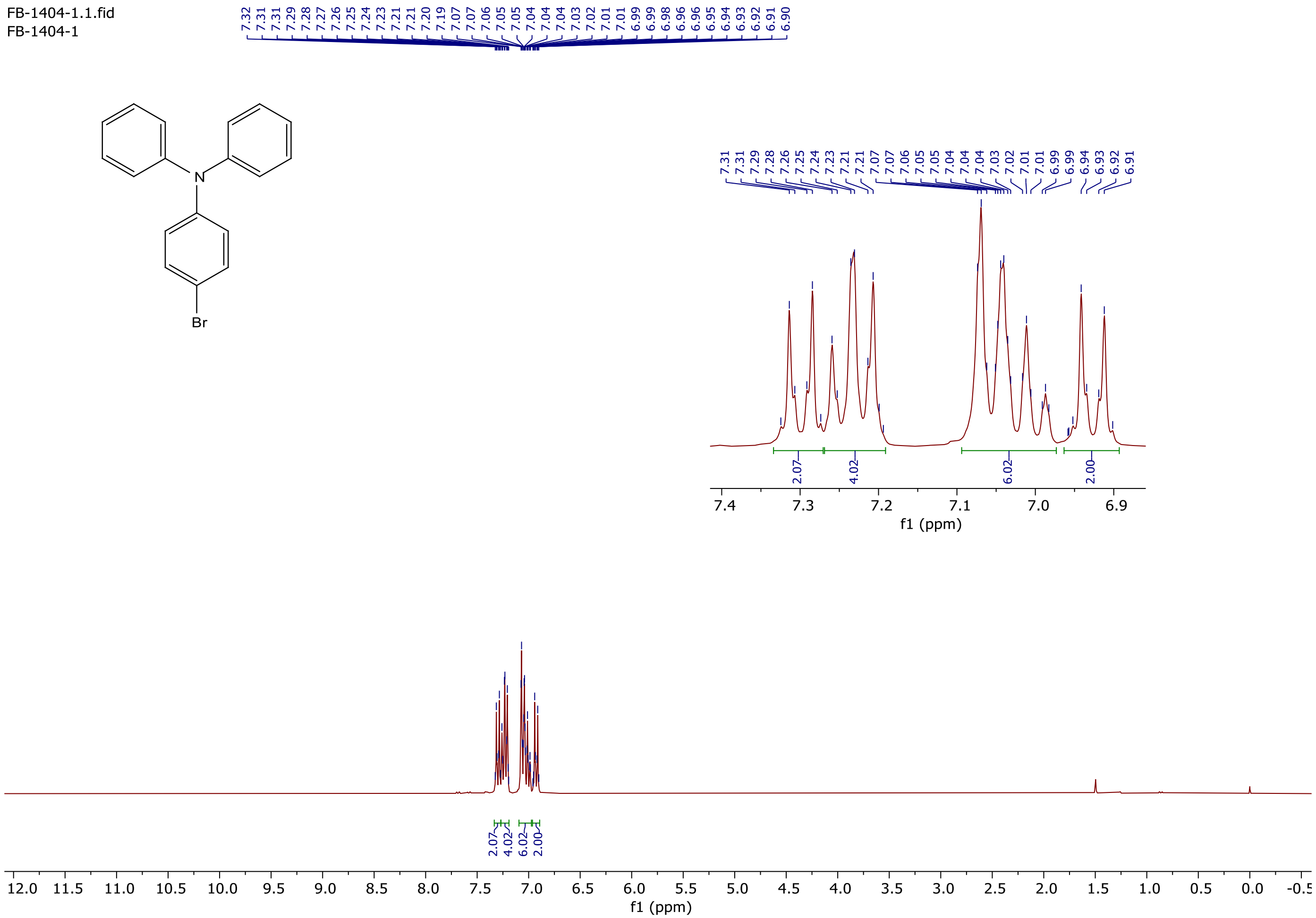

Figure S4. ^{1}H NMR (300 MHz, $CDCl_3$) of 4-Bromo-*N,N*-diphenylaniline **(1)**.

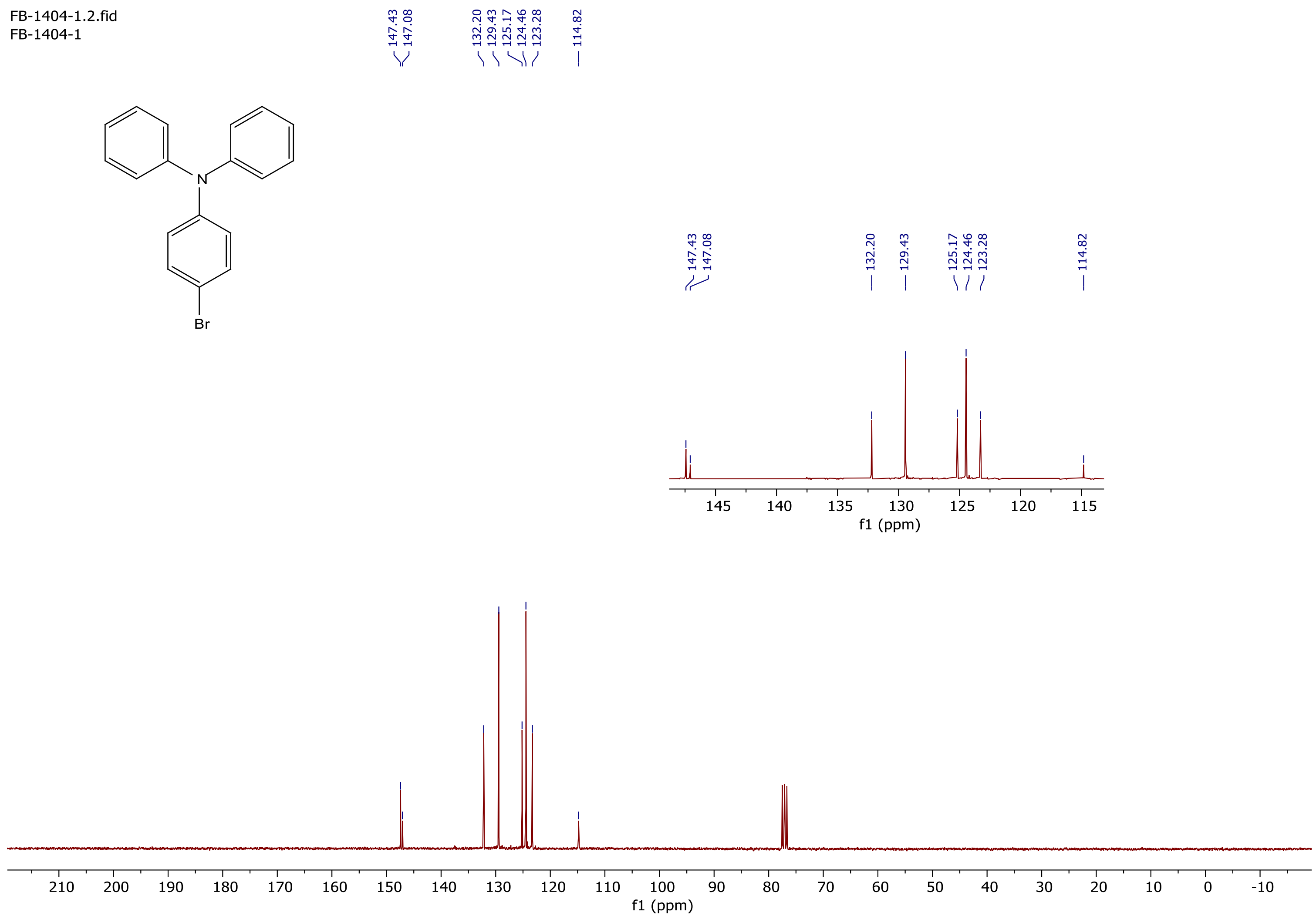

Figure S5. ^{13}C NMR (75 MHz, $CDCl_3$) of 4-Bromo-*N,N*-diphenylaniline **(1)**.

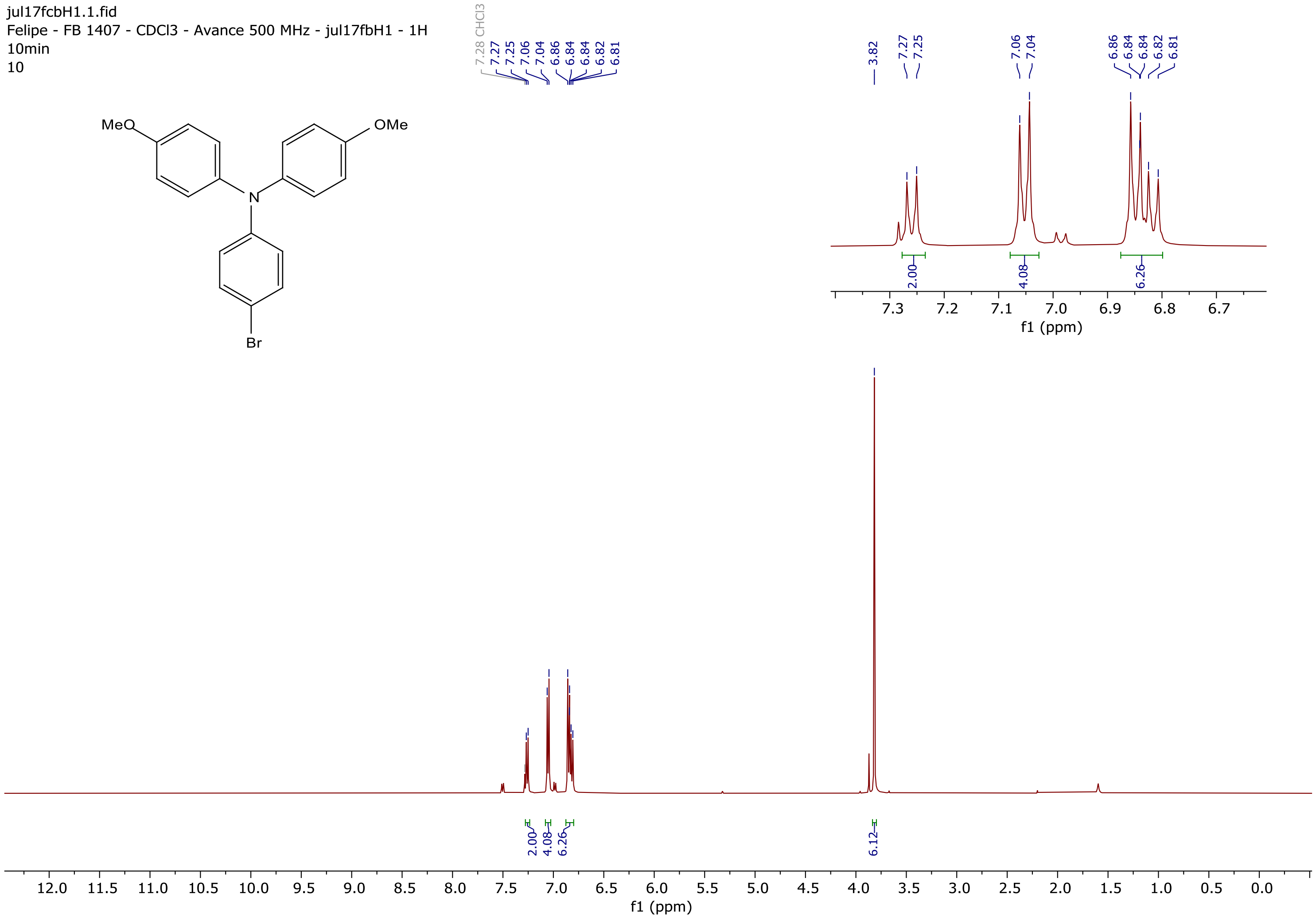

Figure S6. ^{1}H NMR (500 MHz, $CDCl_3$) of 4-bromo-*N,N*-bis(4-methoxyphenyl)aniline **(2)**.

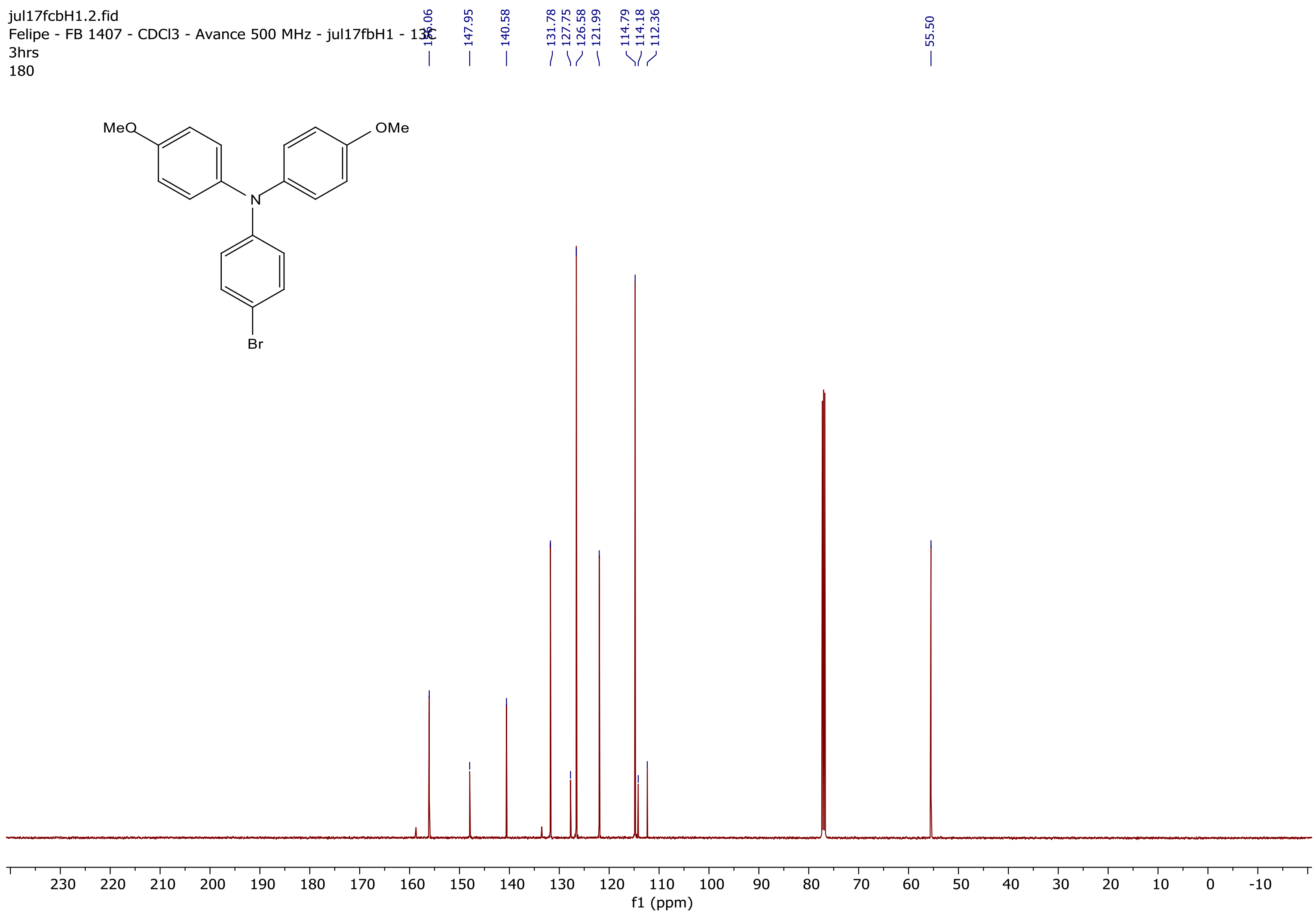

Figure S7. ^{13}C NMR (126 MHz, CDCl3) of 4-bromo-*N,N*-bis(4-methoxyphenyl)aniline **(2)**.

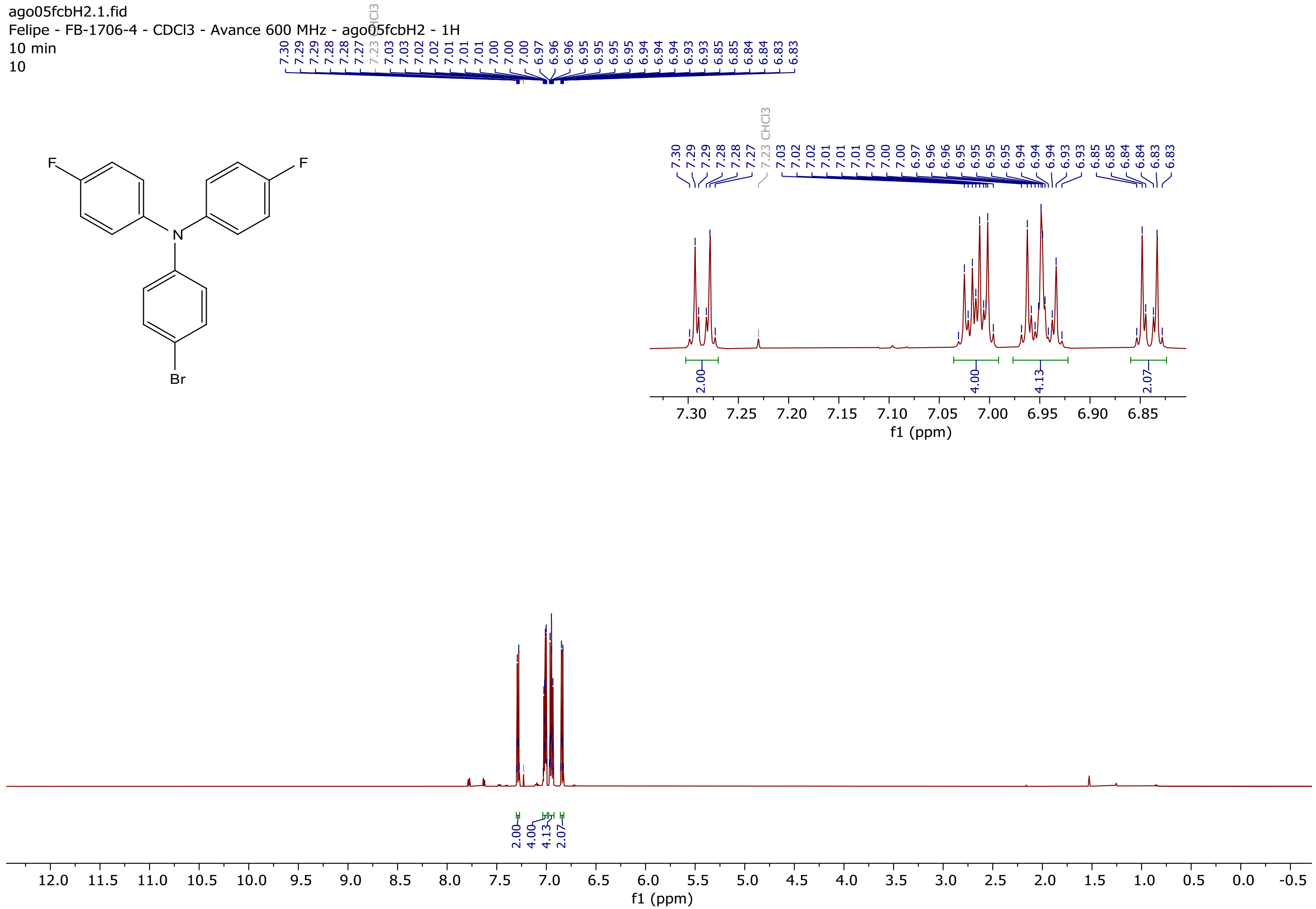

Figure S8. ^{1}H NMR (300 MHz, $CDCl_3$) of 4-bromo-*N,N*-bis(4-fluorophenyl)aniline **(3)**.

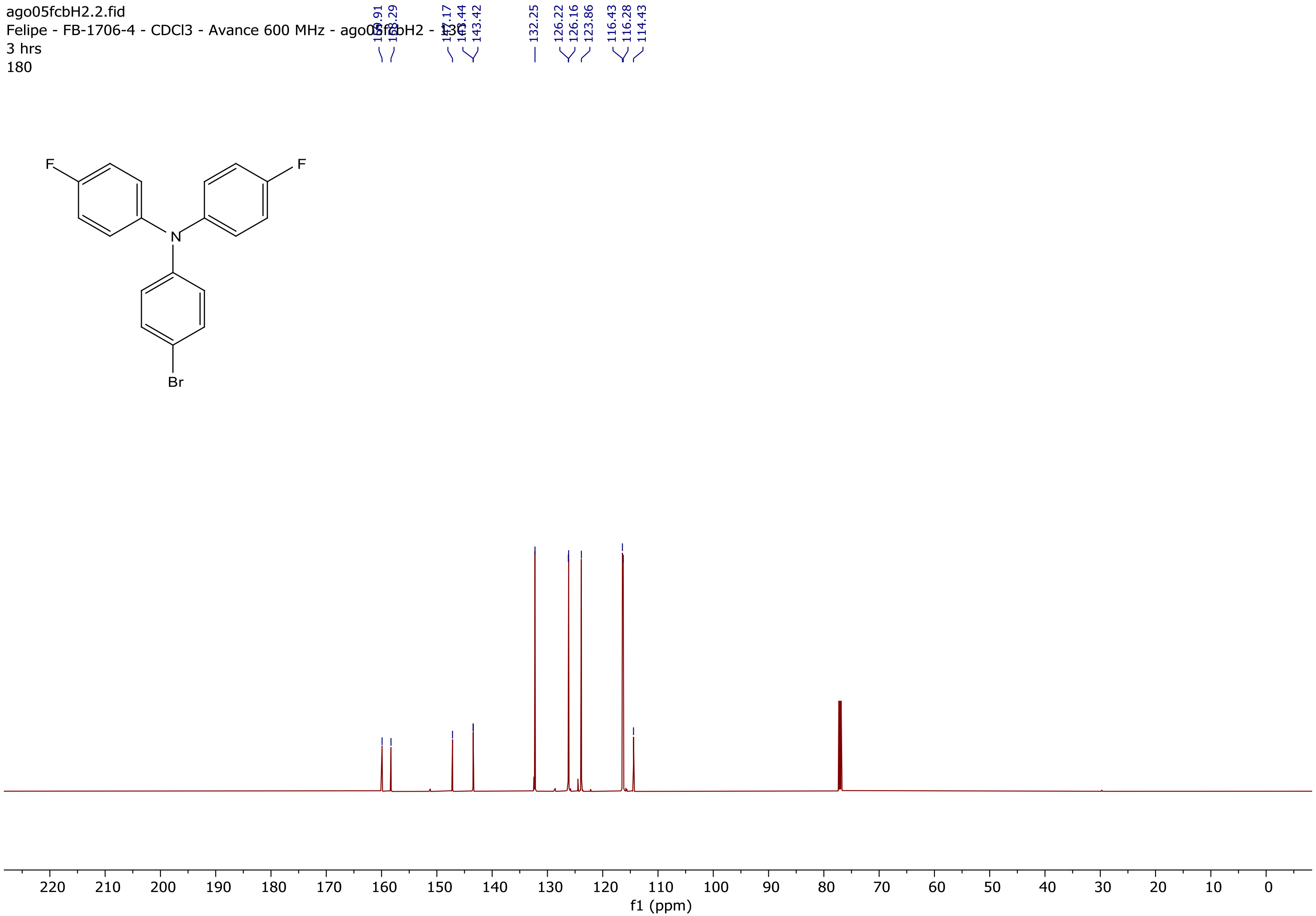

Figure S9. ^{13}C NMR (126 MHz, CDCl3) of 4-bromo-*N,N*-bis(4-fluorophenyl)aniline **(3)**.

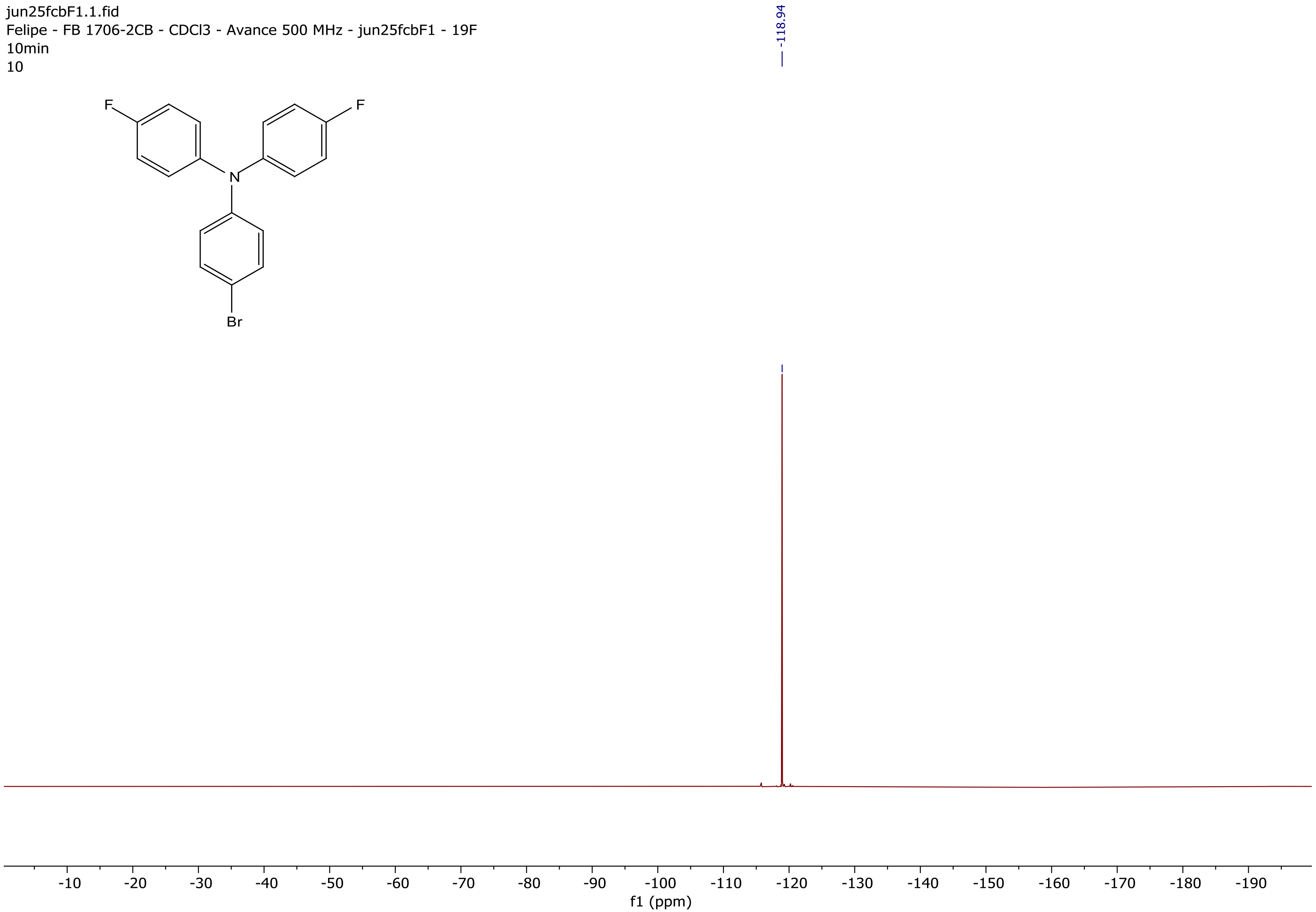

Figure S10. ^{19}F NMR (470 MHz, $CDCl_3$) of 4-bromo-*N,N*-bis(4-fluorophenyl)aniline **(3)**.

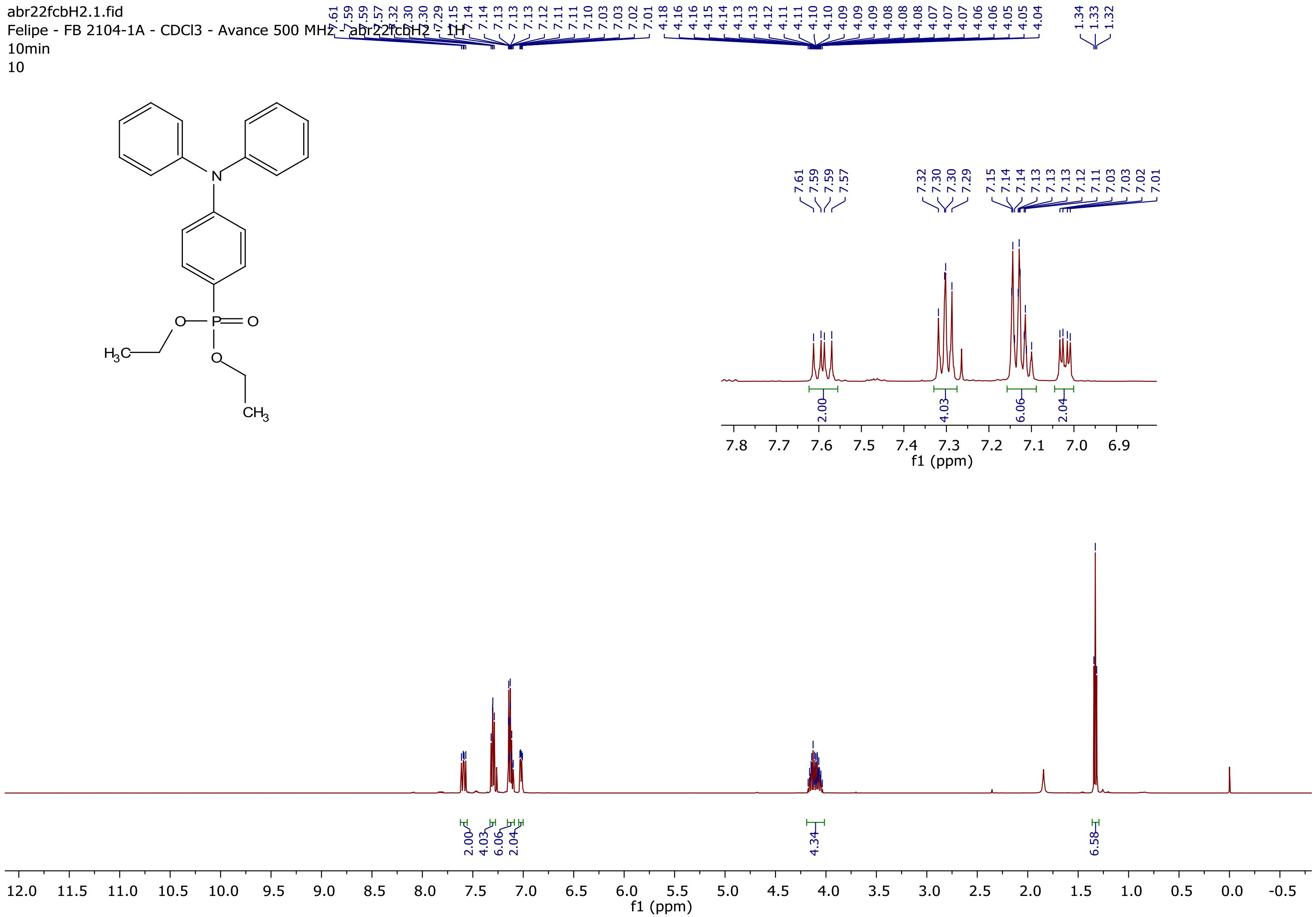

Figure S11. ^{1}H NMR (500 MHz, $CDCl_3$) of diethyl (4-(diphenylamino)phenyl)phosphonate **(4)**.

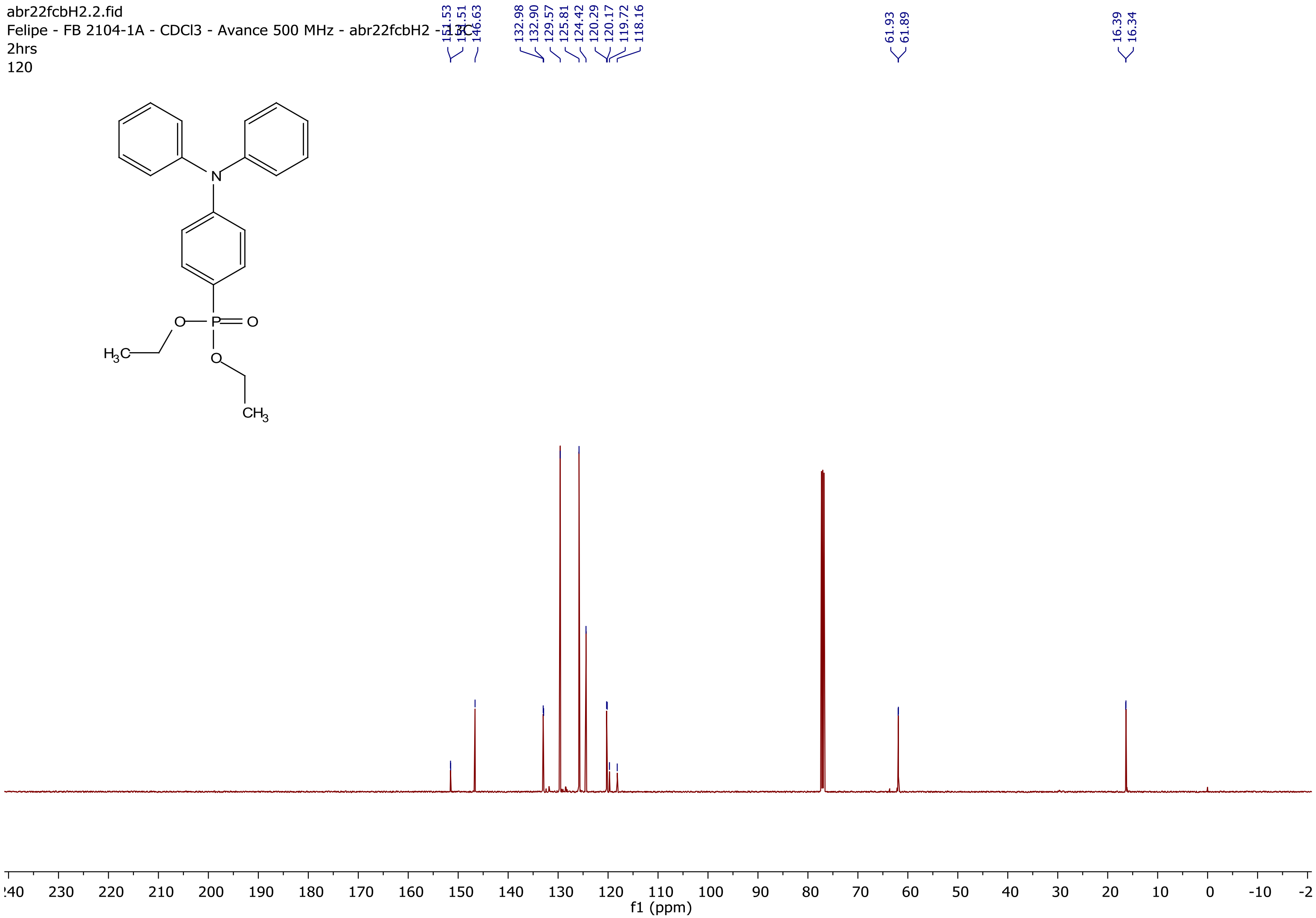

Figure S12. ^{13}C NMR (126 MHz, $CDCl_3$) of diethyl (4-(diphenylamino)phenyl)phosphonate **(4)**.

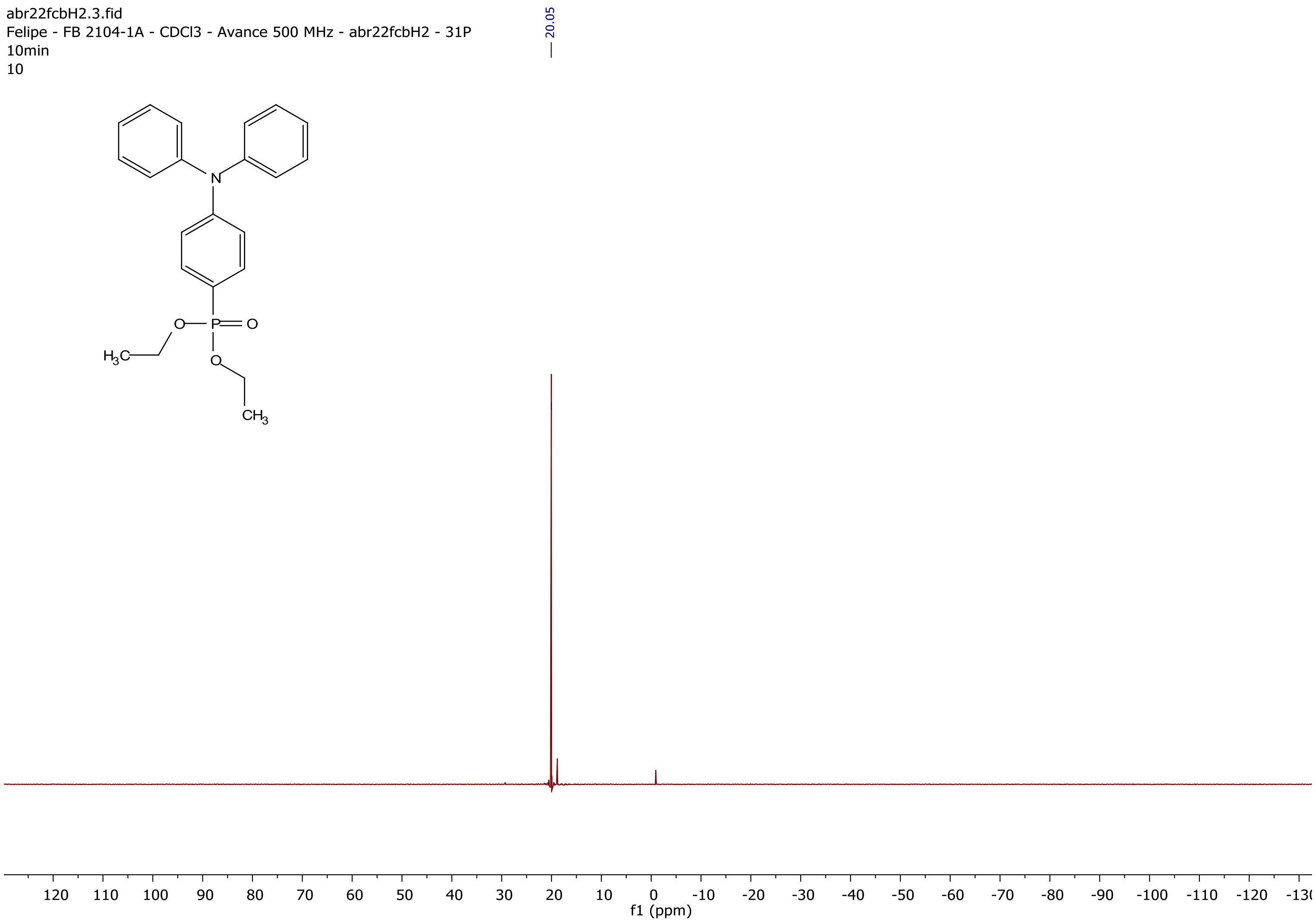

Figure S13. ^{31}P NMR (202 MHz, $CDCl_3$) of diethyl (4-(diphenylamino)phenyl)phosphonate **(4)**.

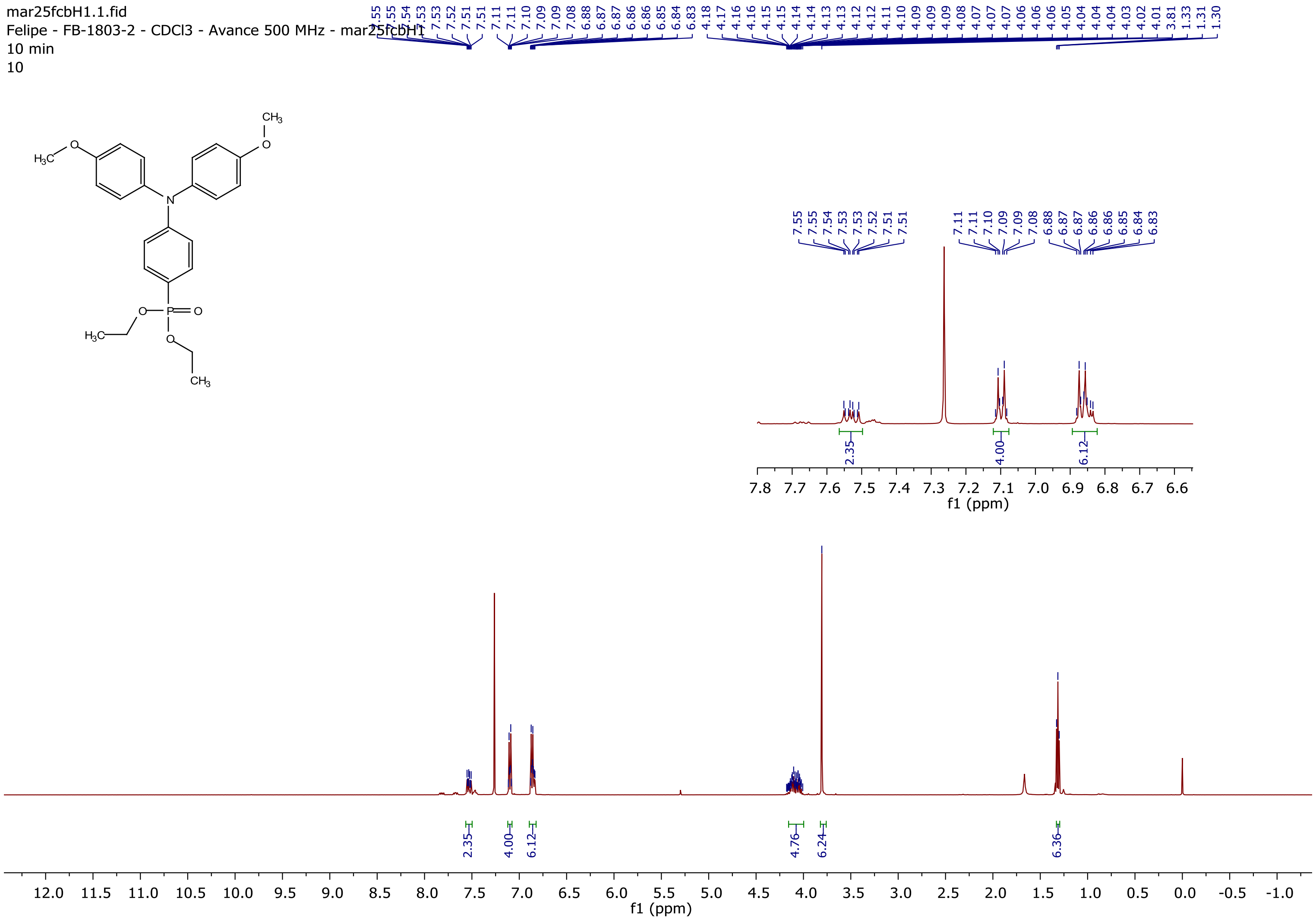

Figure S14. ^{1}H NMR (500 MHz, $CDCl_3$) of diethyl (4-(bis(4-methoxyphenyl)amino)phenyl) phosphonate **(5)**.

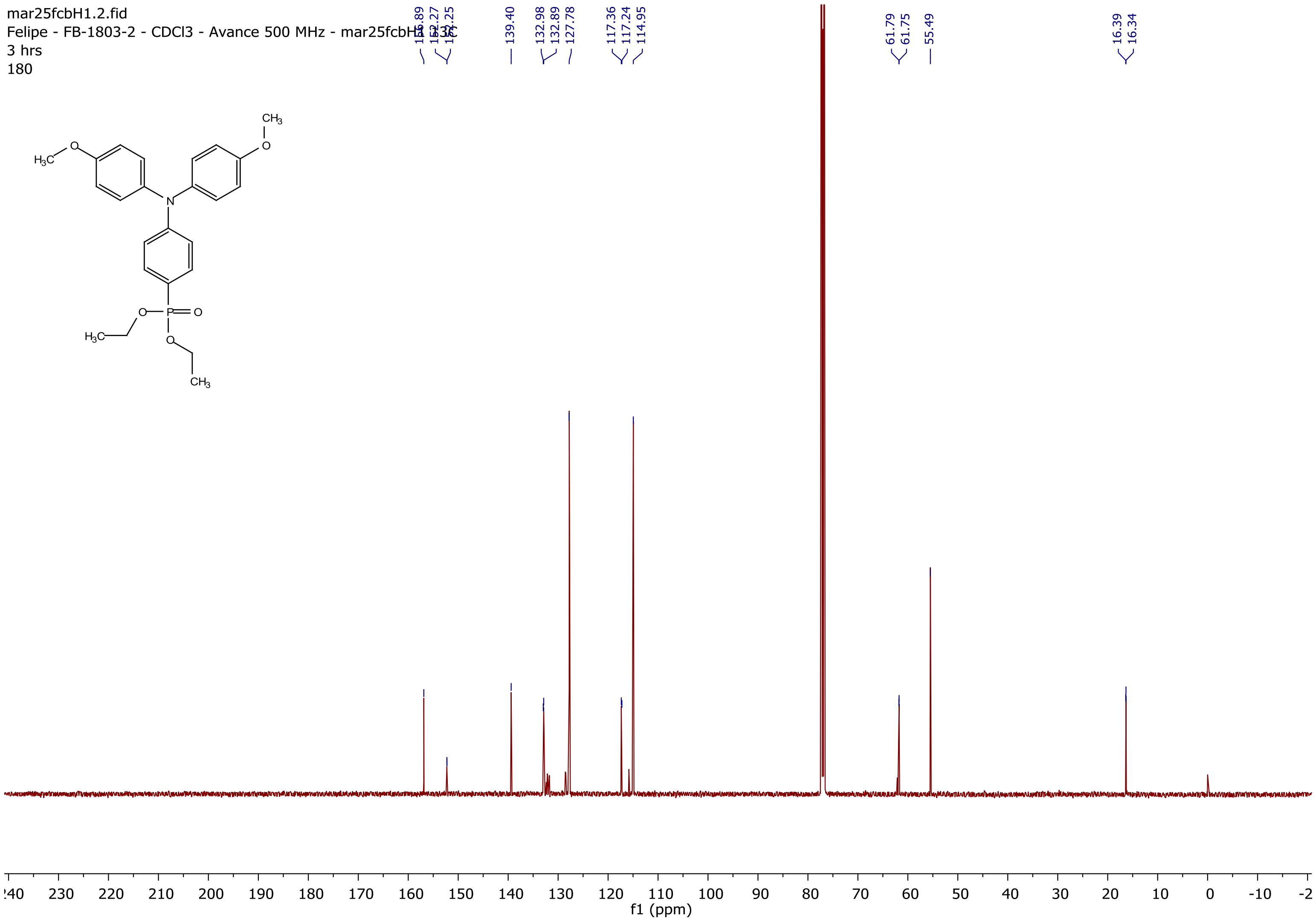

Figure S15. ^{13}C NMR (126 MHz, $CDCl_3$) of diethyl (4-(bis(4-methoxyphenyl)amino)phenyl) phosphonate **(5)**.

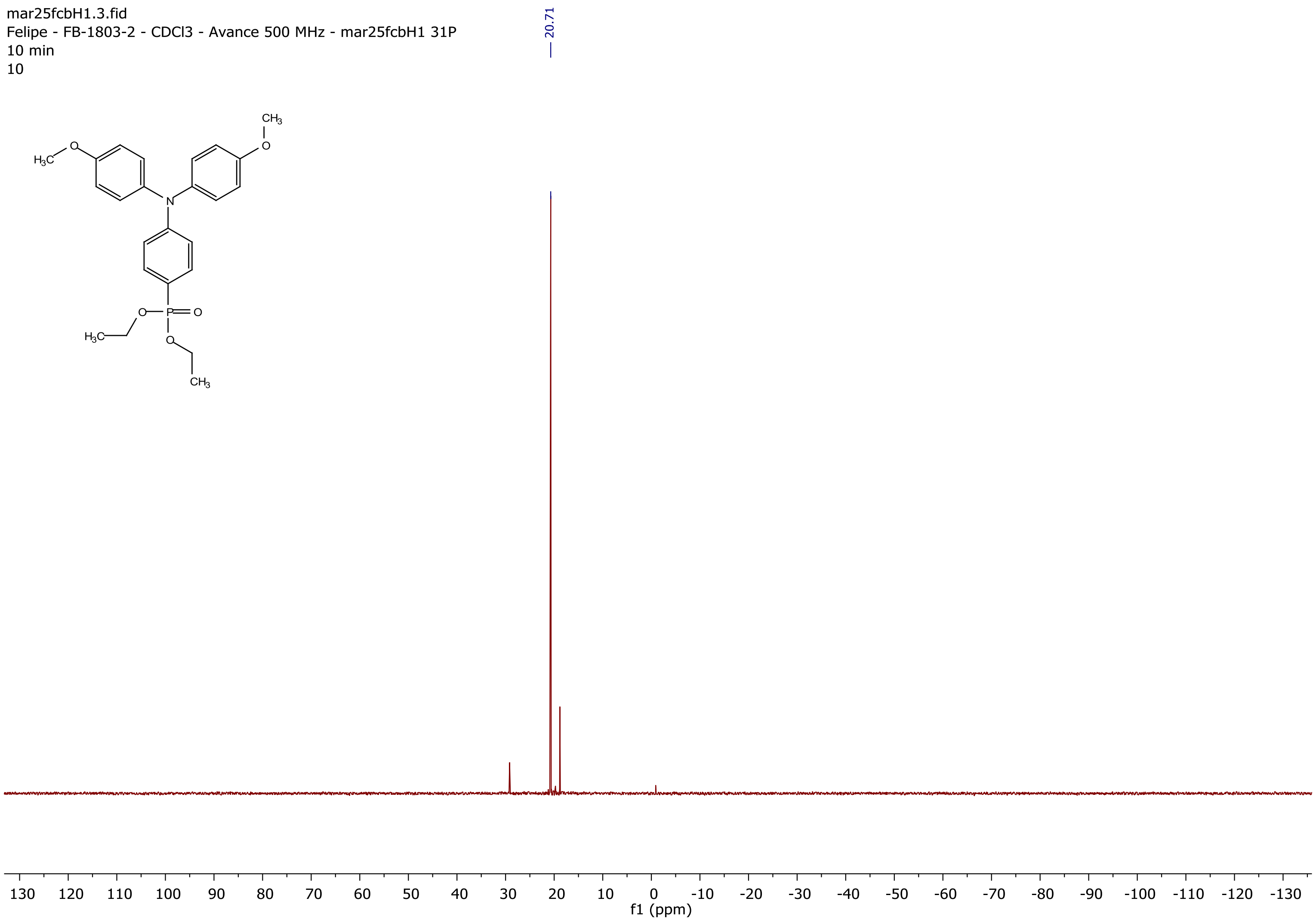

Figure S 16. ^{31}P NMR (202 MHz, $CDCl_3$) of diethyl (4-(bis(4-methoxyphenyl)amino)phenyl) phosphonate **(5)**.

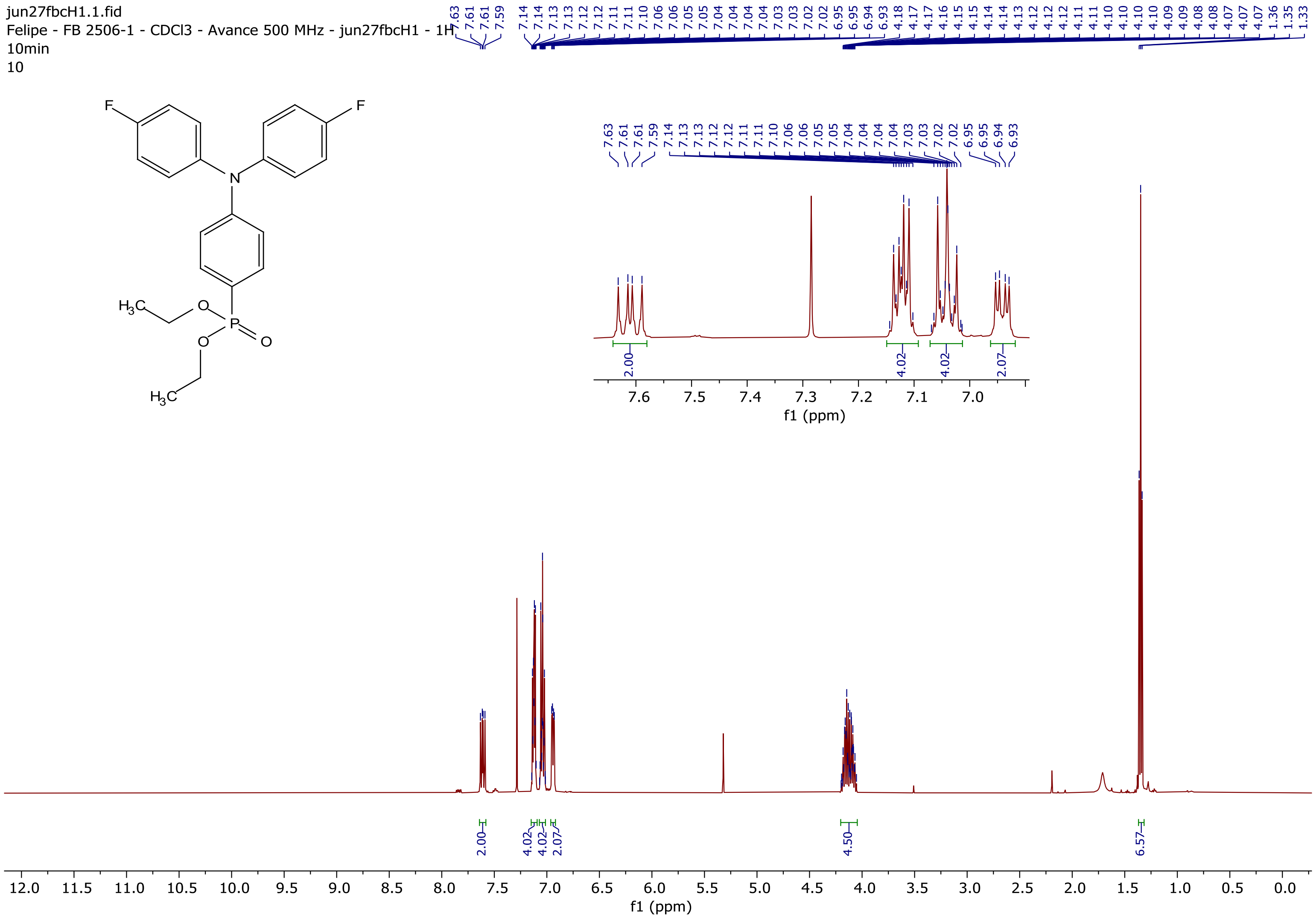

Figure S17. ^{1}H NMR (500 MHz, $CDCl_3$) of diethyl (4-(bis(4-fluorophenyl)amino)phenyl)phosphonate **(6)**.

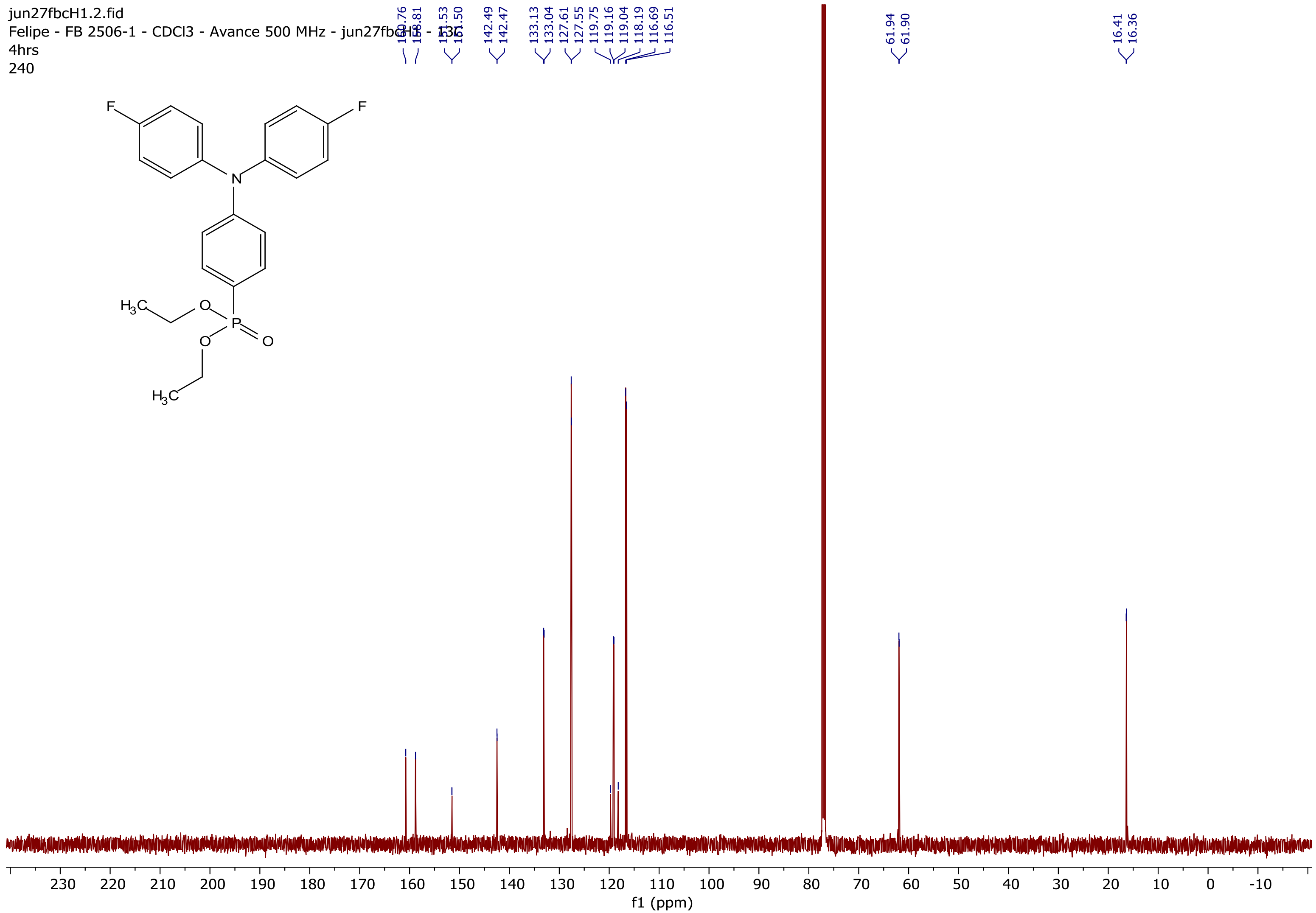

Figure S18. ^{13}C NMR (126 MHz, $CDCl_3$) of diethyl (4-(bis(4-fluorophenyl)amino)phenyl)phosphonate **(6)**.

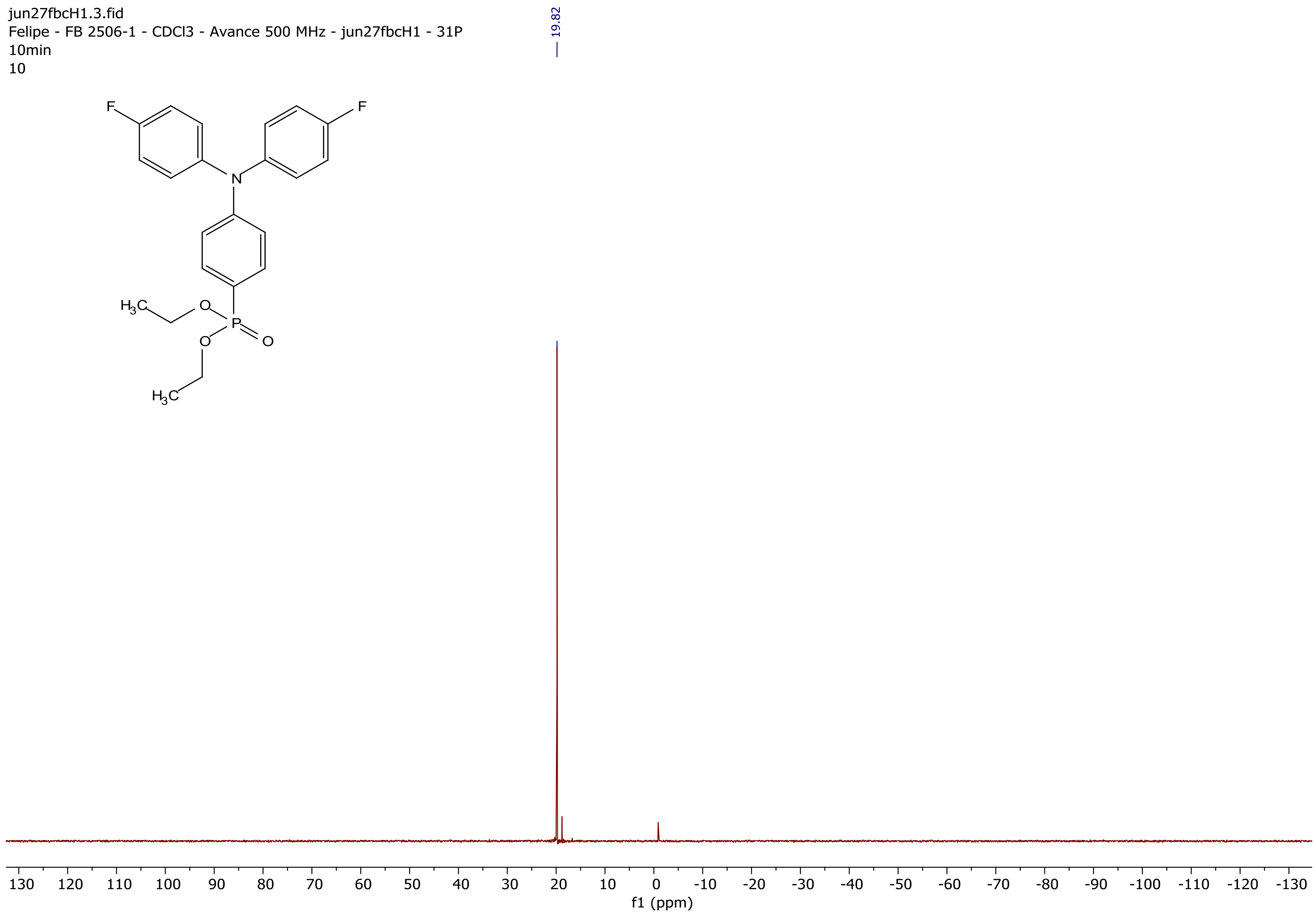

Figure S19. ^{31}P NMR (202 MHz, $CDCl_3$) of diethyl (4-(bis(4-fluorophenyl)amino)phenyl)phosphonate **(6)**.

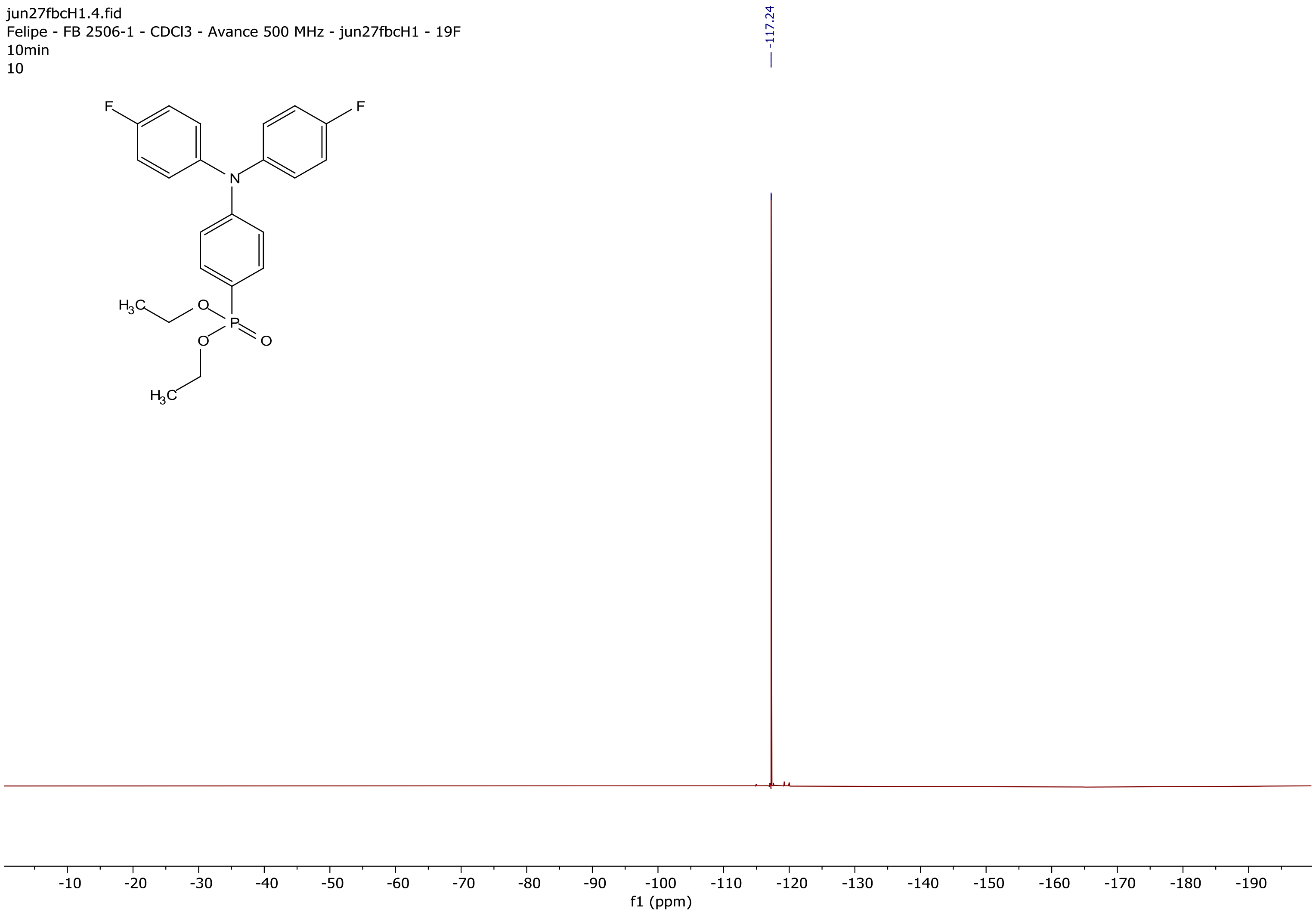

Figure S20. ^{19}F NMR (470 MHz, $CDCl_3$) of diethyl (4-(bis(4-fluorophenyl)amino)phenyl)phosphonate **(6)**.

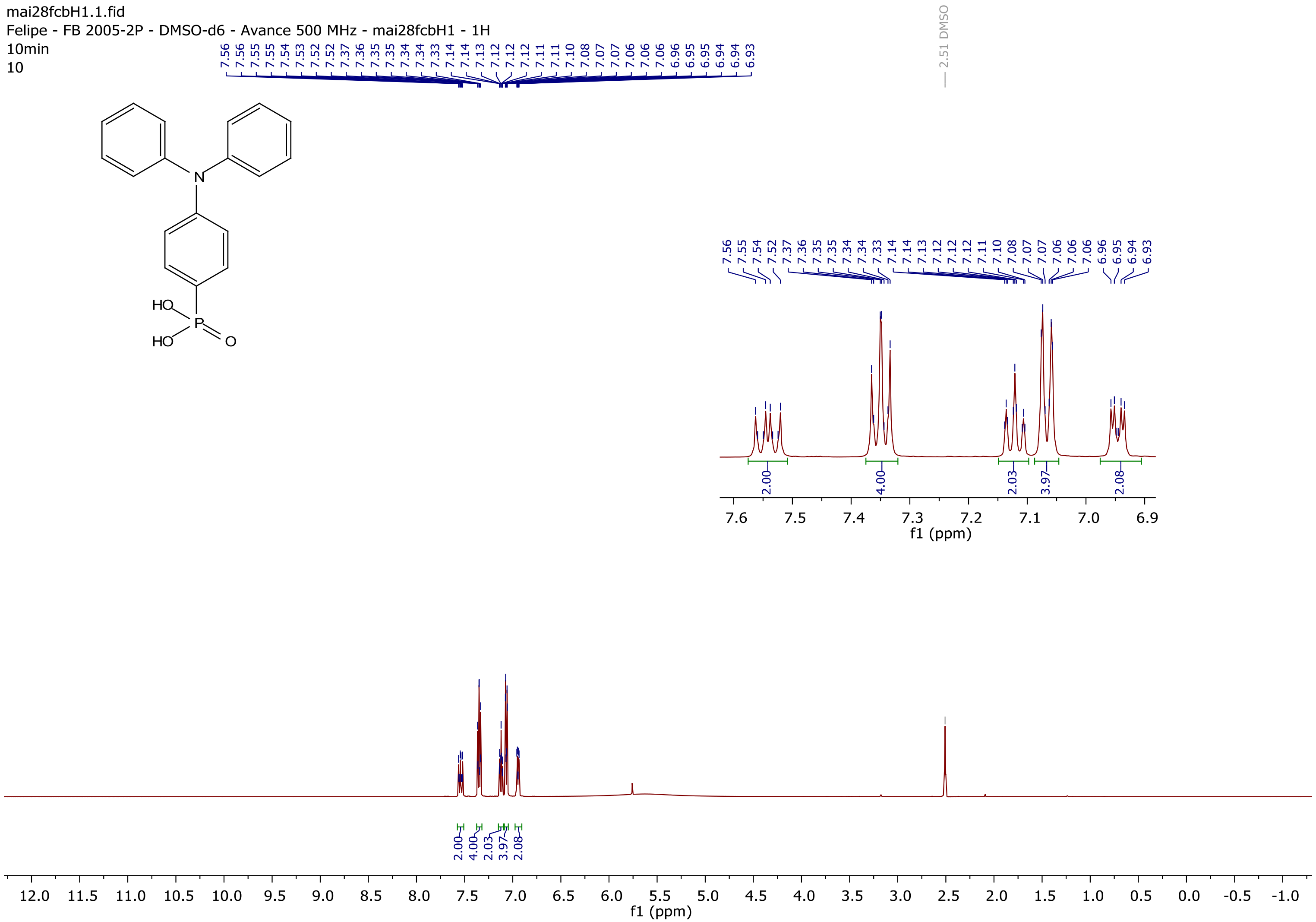

Figure S21. ^{1}H NMR (500 MHz, DMSO-d_6) of (4-(diphenylamino)phenyl)phosphonic acid **(7)**.

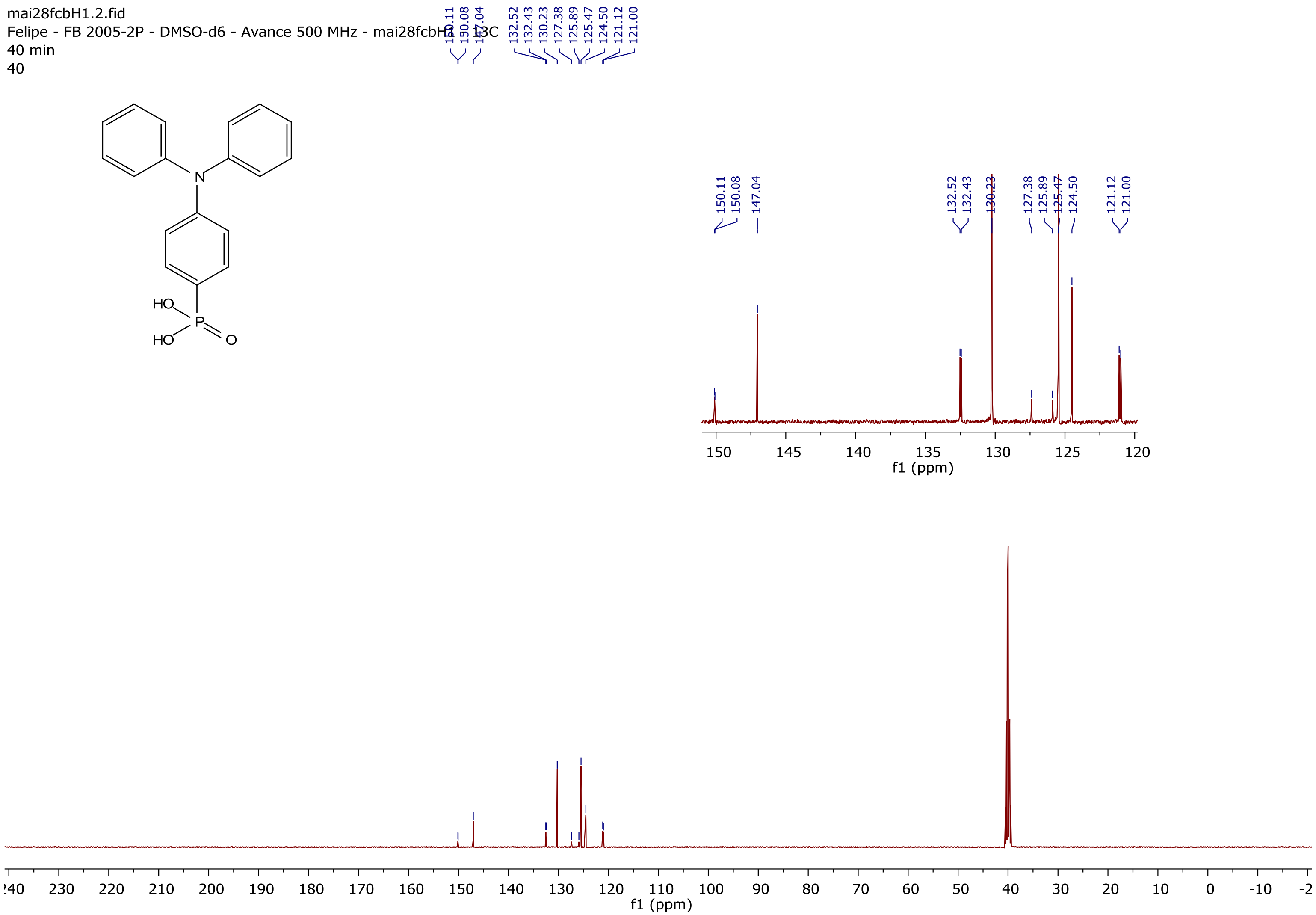

Figure S22. ^{13}C NMR (126 MHz, DMSO-d_6) of (4-(diphenylamino)phenyl)phosphonic acid **(7)**.

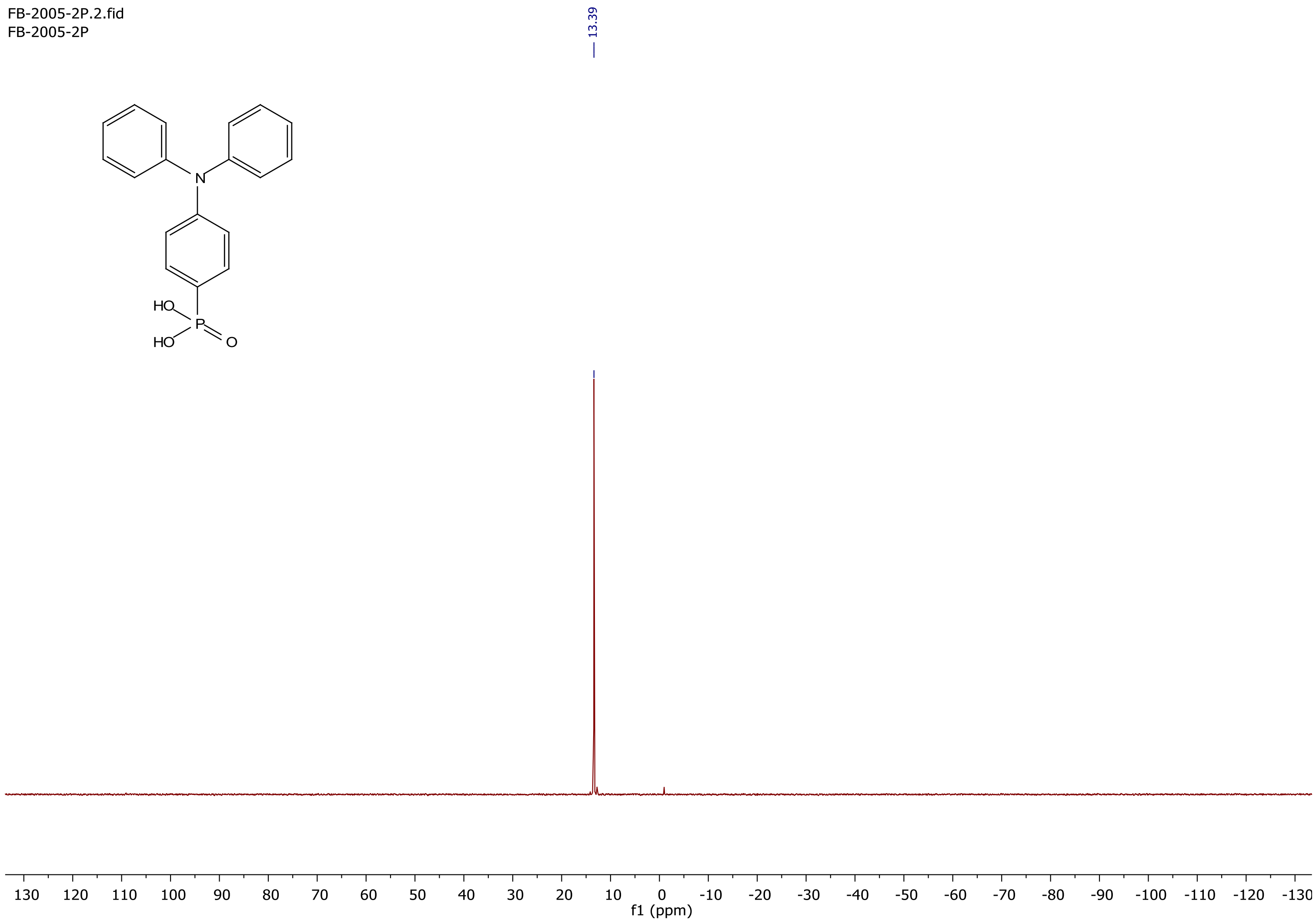

Figure S23. ^{31}P NMR (121 MHz, DMSO-d_6) of (4-(diphenylamino)phenyl)phosphonic acid **(7)**.

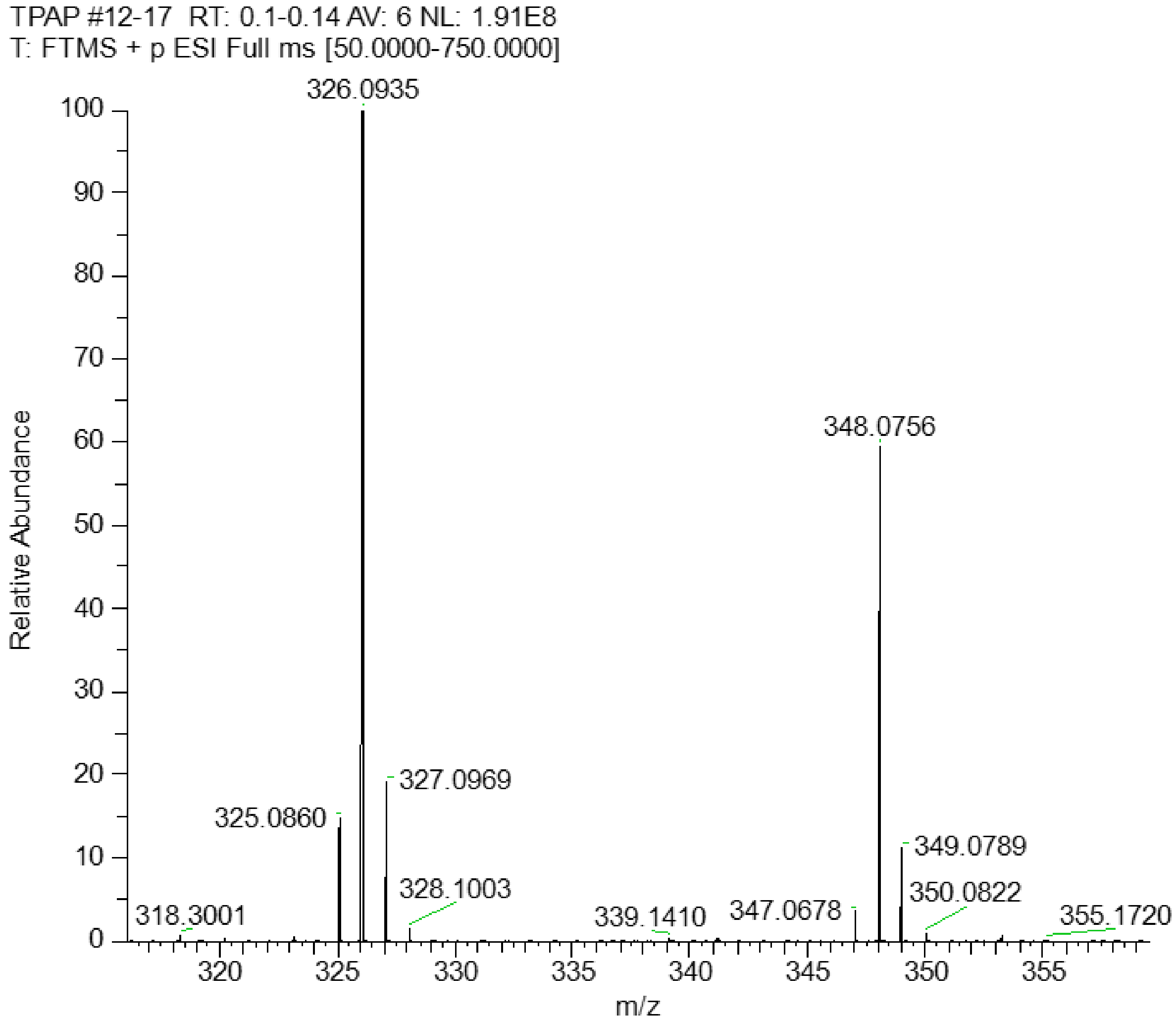

Figure S24. HRMS Spectra of (4-(diphenylamino)phenyl)phosphonic acid **(7)**.

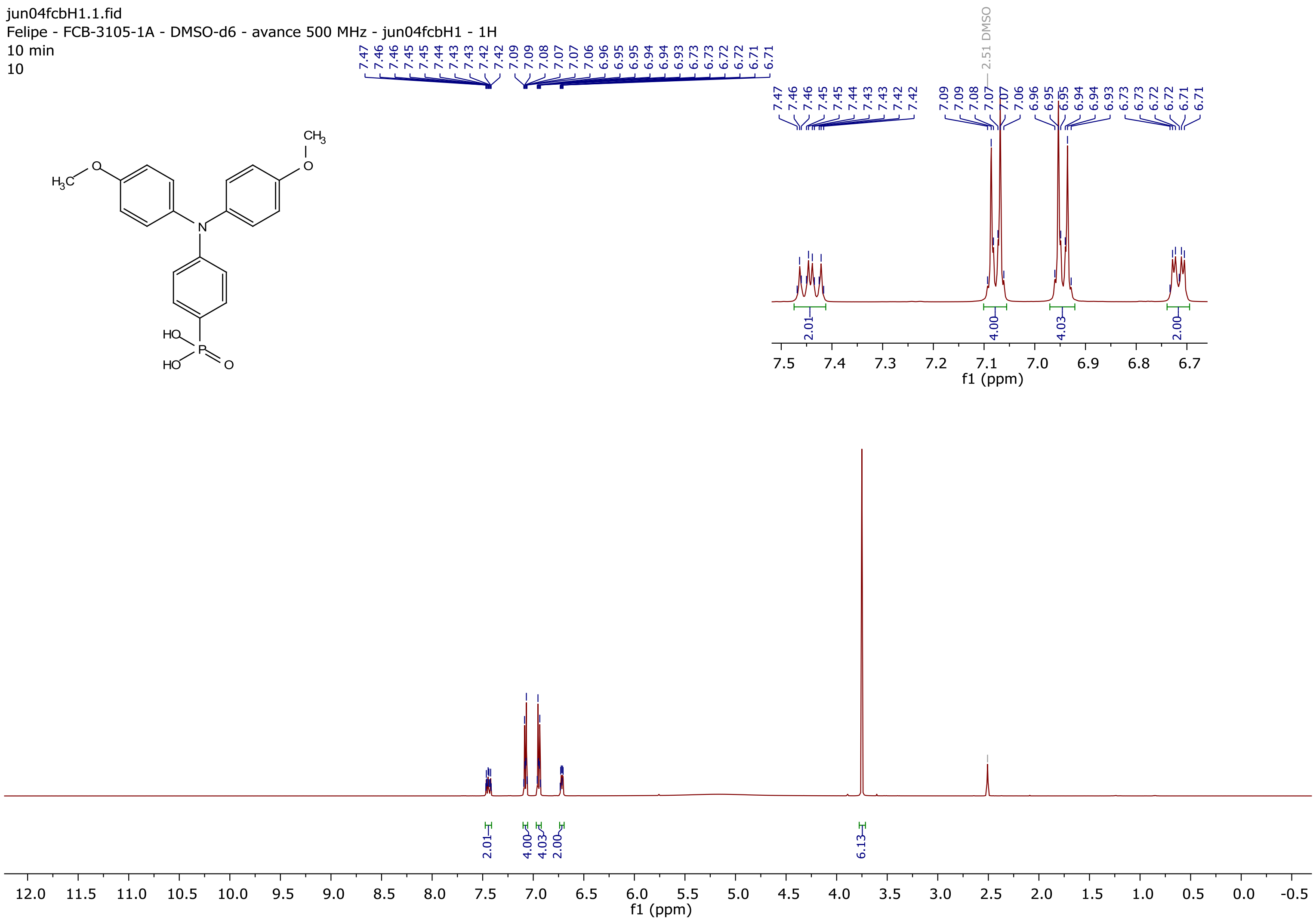

Figure S25. ^{1}H NMR (500 MHz, DMSO-d$_6$) of (4-(bis(4-methoxyphenyl)amino)phenyl)phosphonic acid **(8)**.

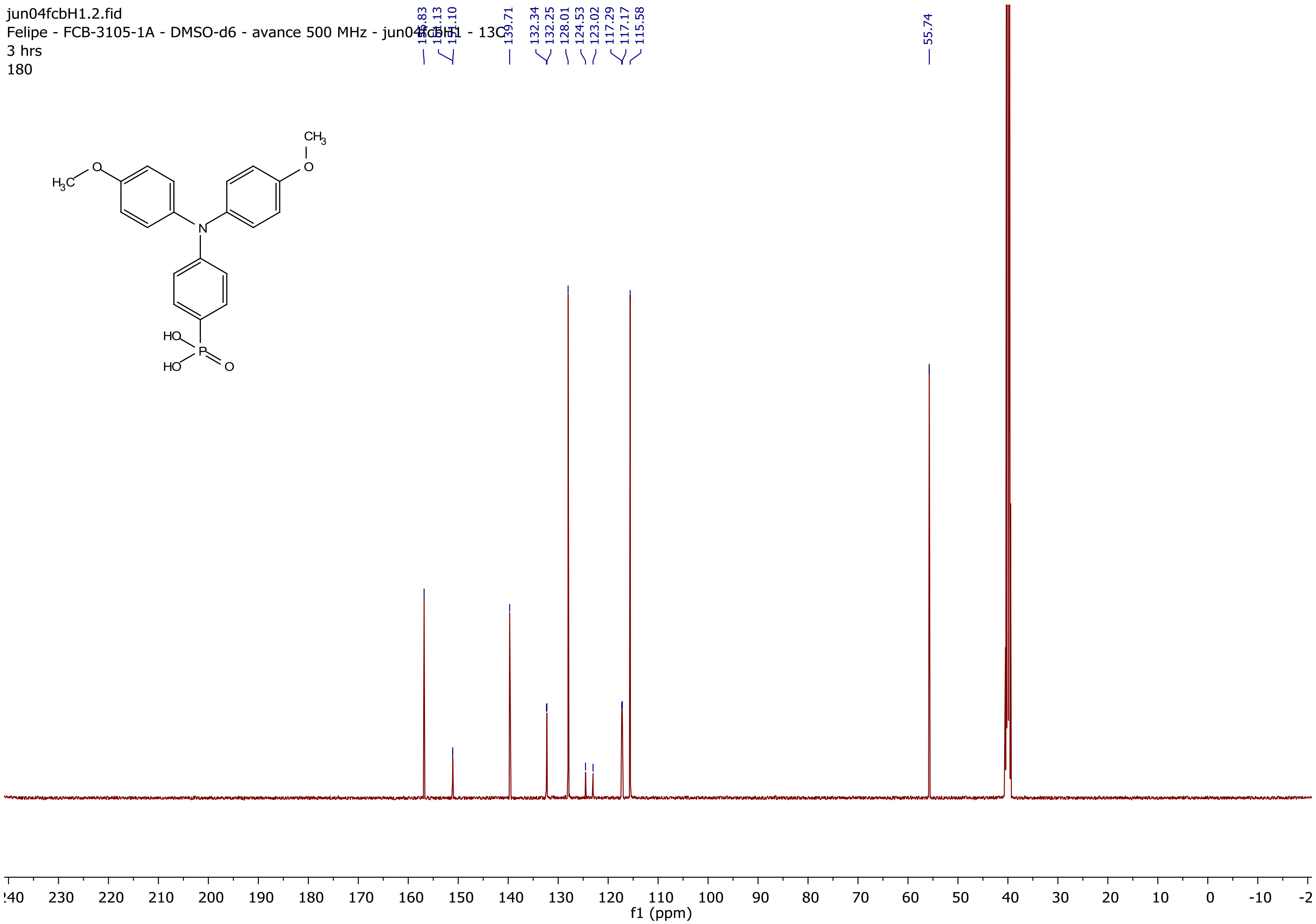

Figure S26. ^{13}C NMR (126 MHz, DMSO-d_6) of (4-(bis(4-methoxyphenyl)amino)phenyl)phosphonic acid **(8)**.

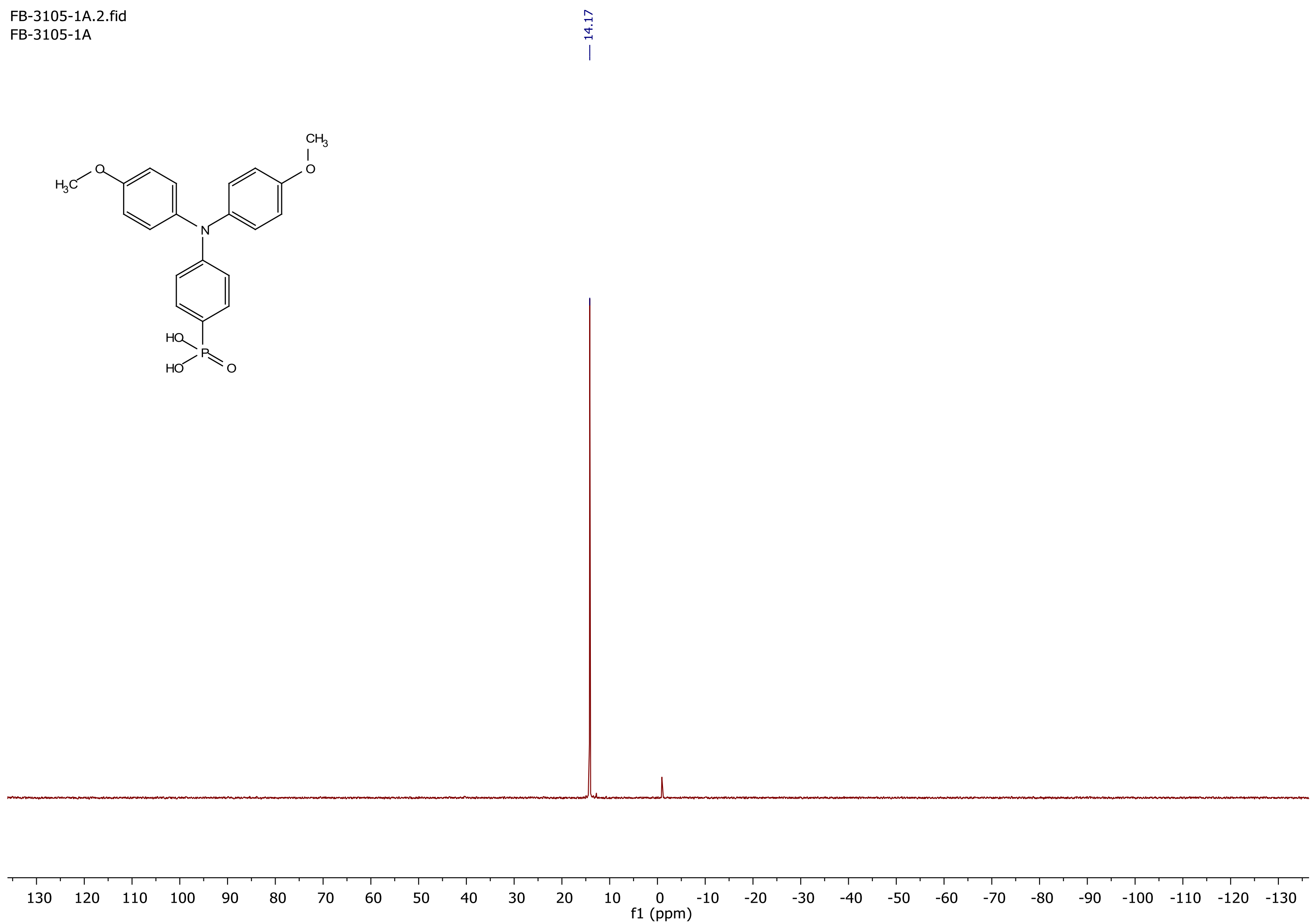

Figure S27. ^{31}P NMR (121 MHz, DMSO-d_6) of (4-(bis(4-methoxyphenyl)amino)phenyl)phosphonic acid **(8)**.

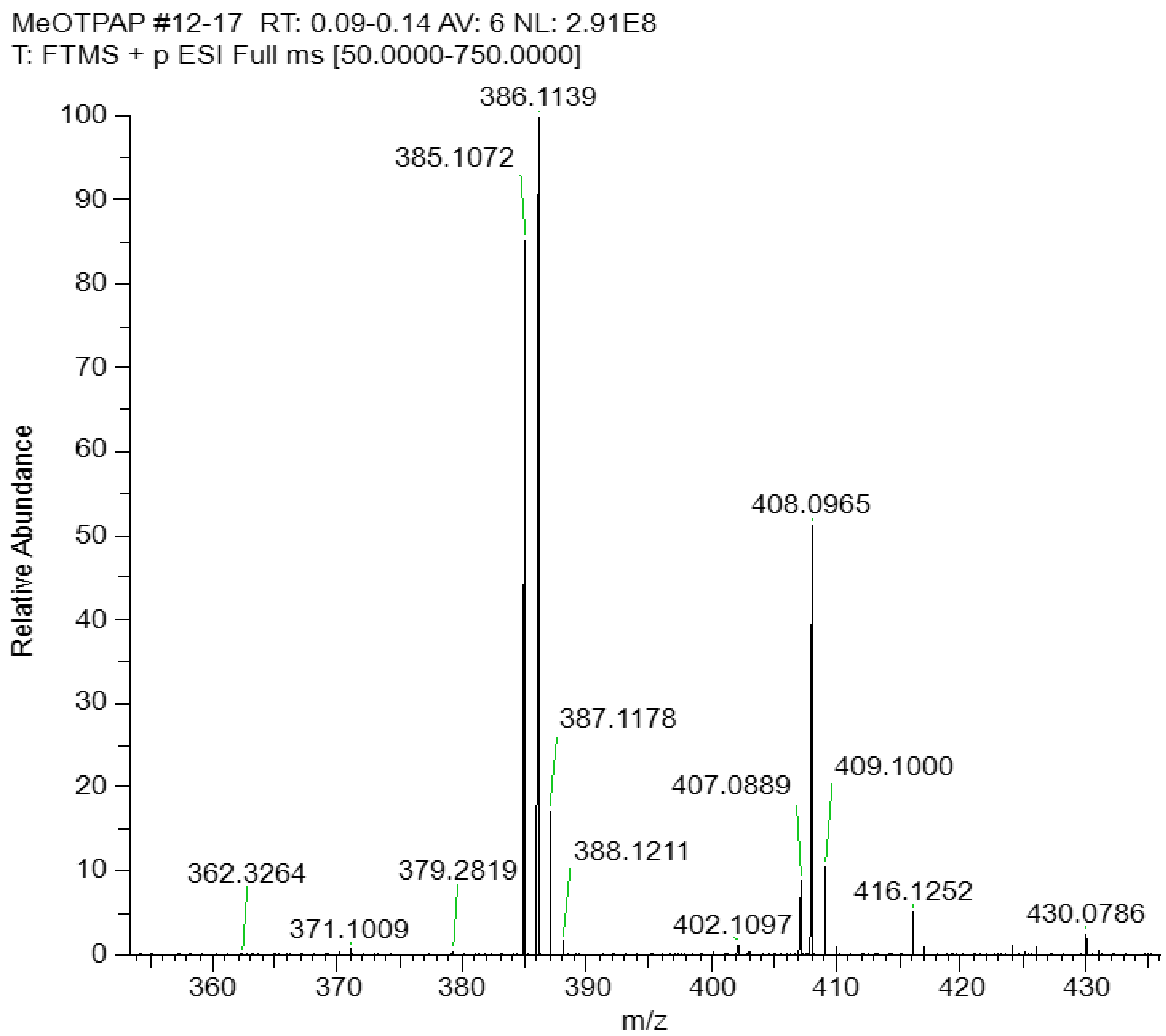

Figure S28. HRMS of (4-(bis(4-methoxyphenyl)amino)phenyl)phosphonic acid **(8)**.

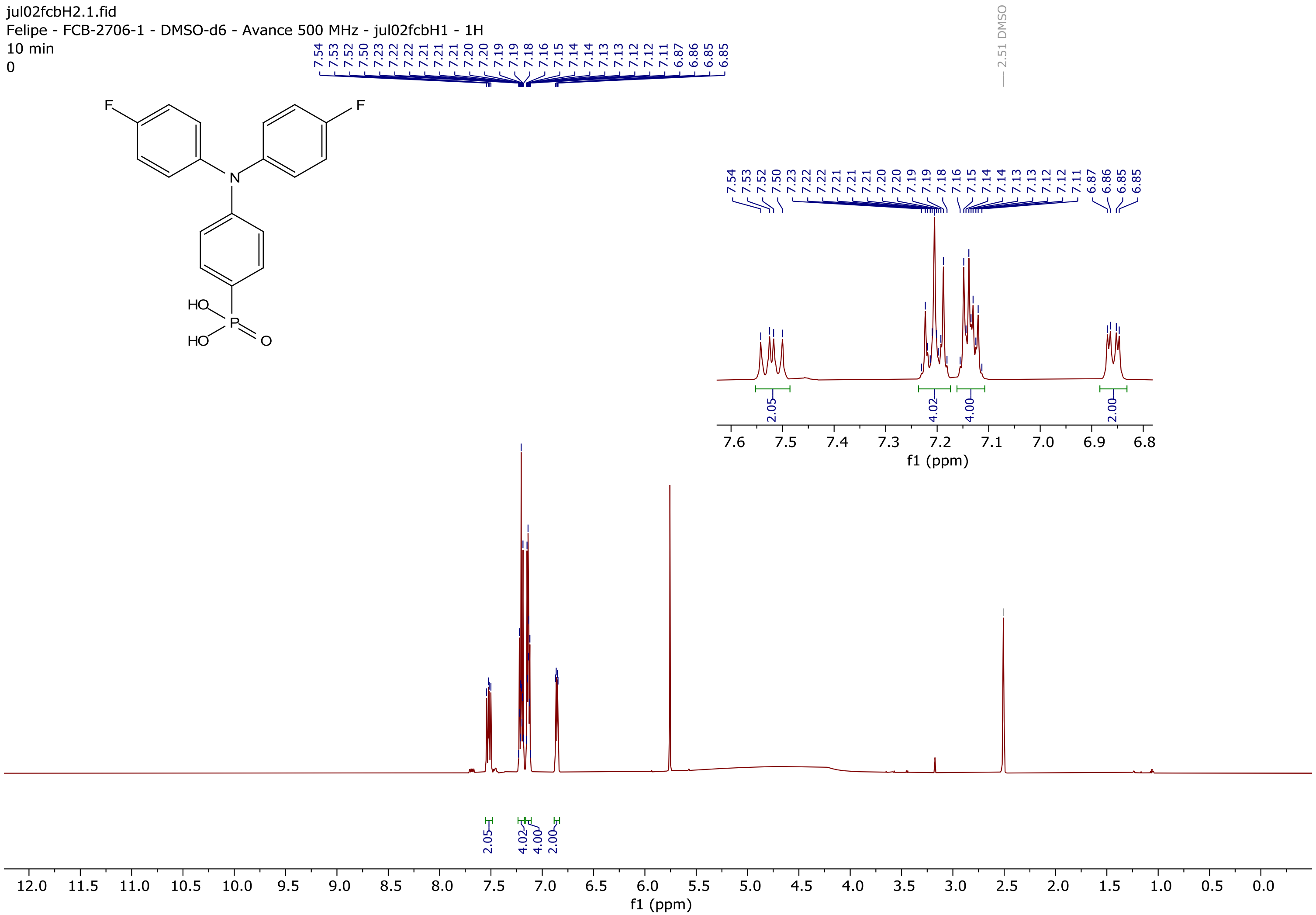

Figure S29. ^{1}H NMR (500 MHz, DMSO-d_6) of (4-(bis(4-fluorophenyl)amino)phenyl)phosphonic acid **(9)**.

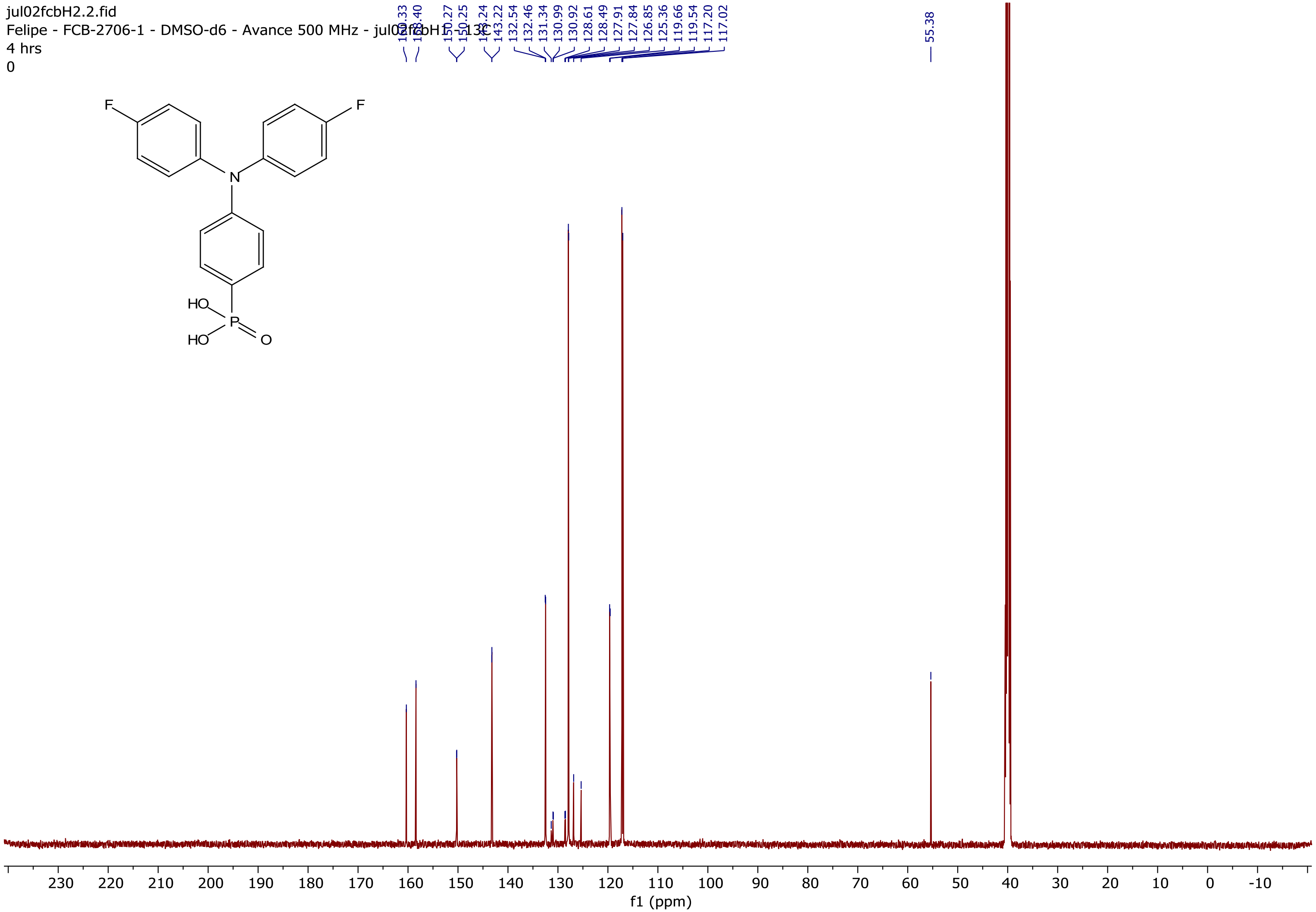

Figure S30. ^{13}C NMR (126 MHz, DMSO-d_6) of (4-(bis(4-fluorophenyl)amino)phenyl)phosphonic acid **(9)**.

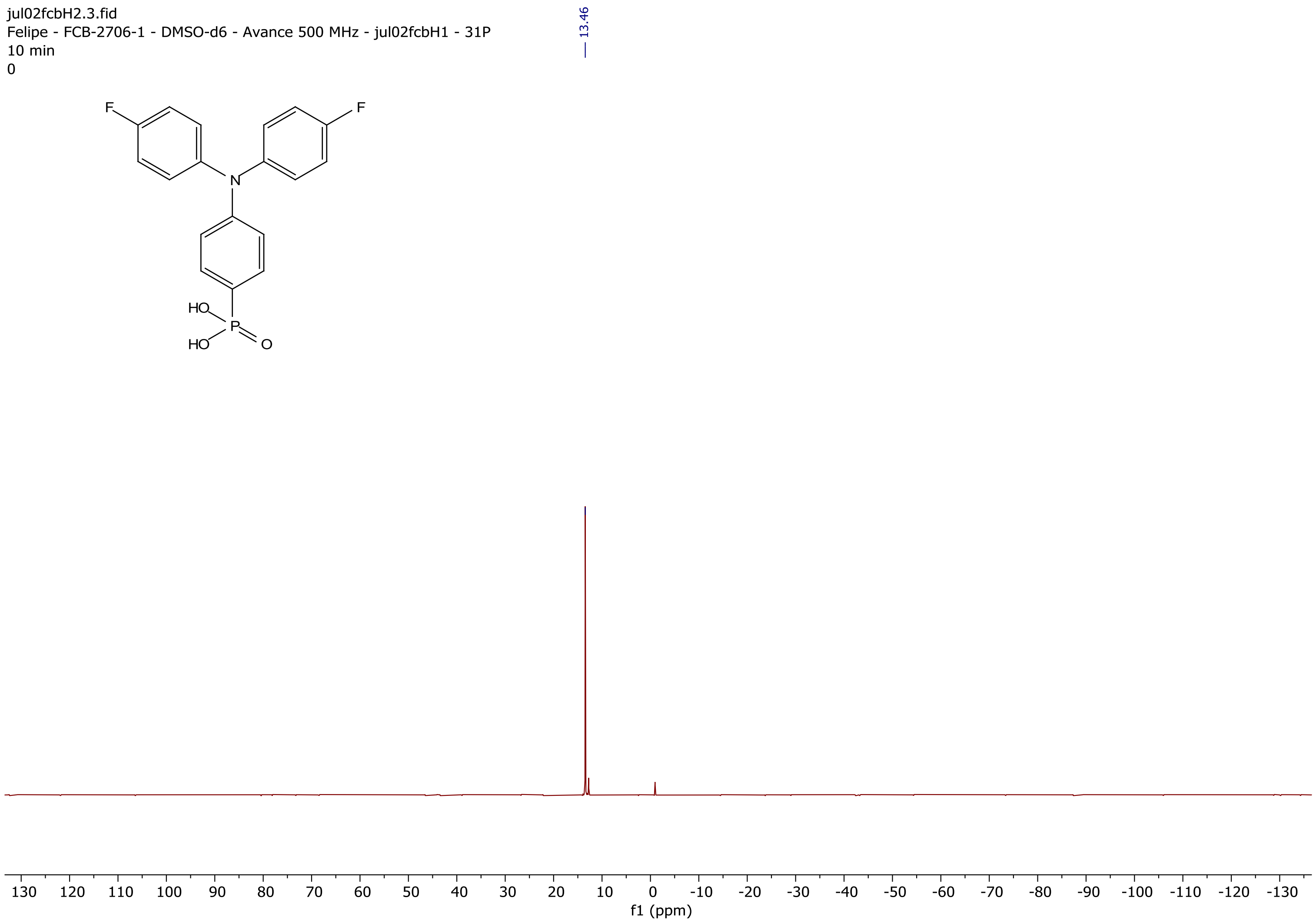

Figure S31. ^{31}P NMR (202 MHz, DMSO-d_6) of (4-(bis(4-fluorophenyl)amino)phenyl)phosphonic acid **(9)**.

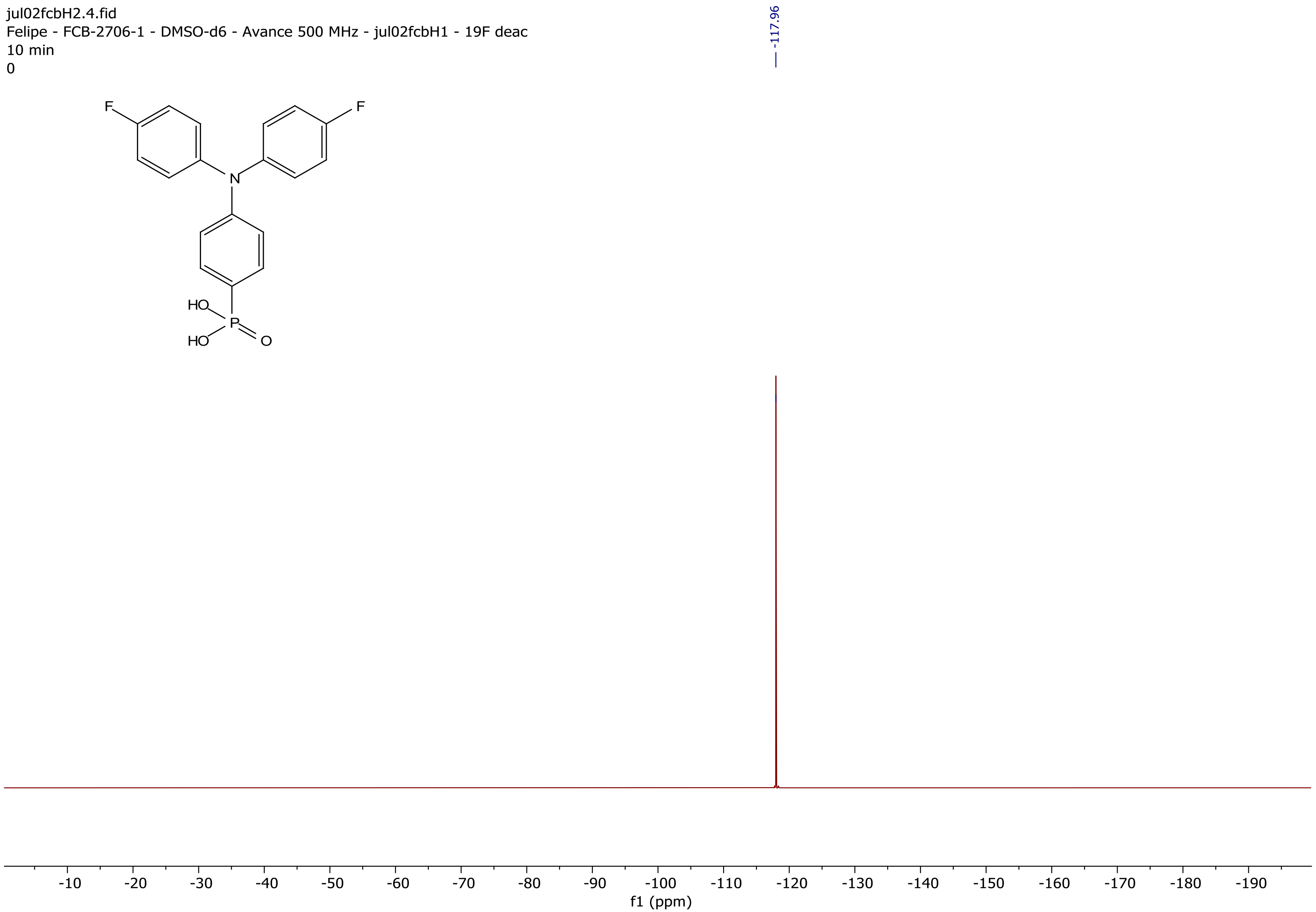

Figure S32. ^{19}F NMR (470 MHz, $CDCl_3$) of (4-(bis(4-fluorophenyl)amino)phenyl)phosphonic acid **(9)**.

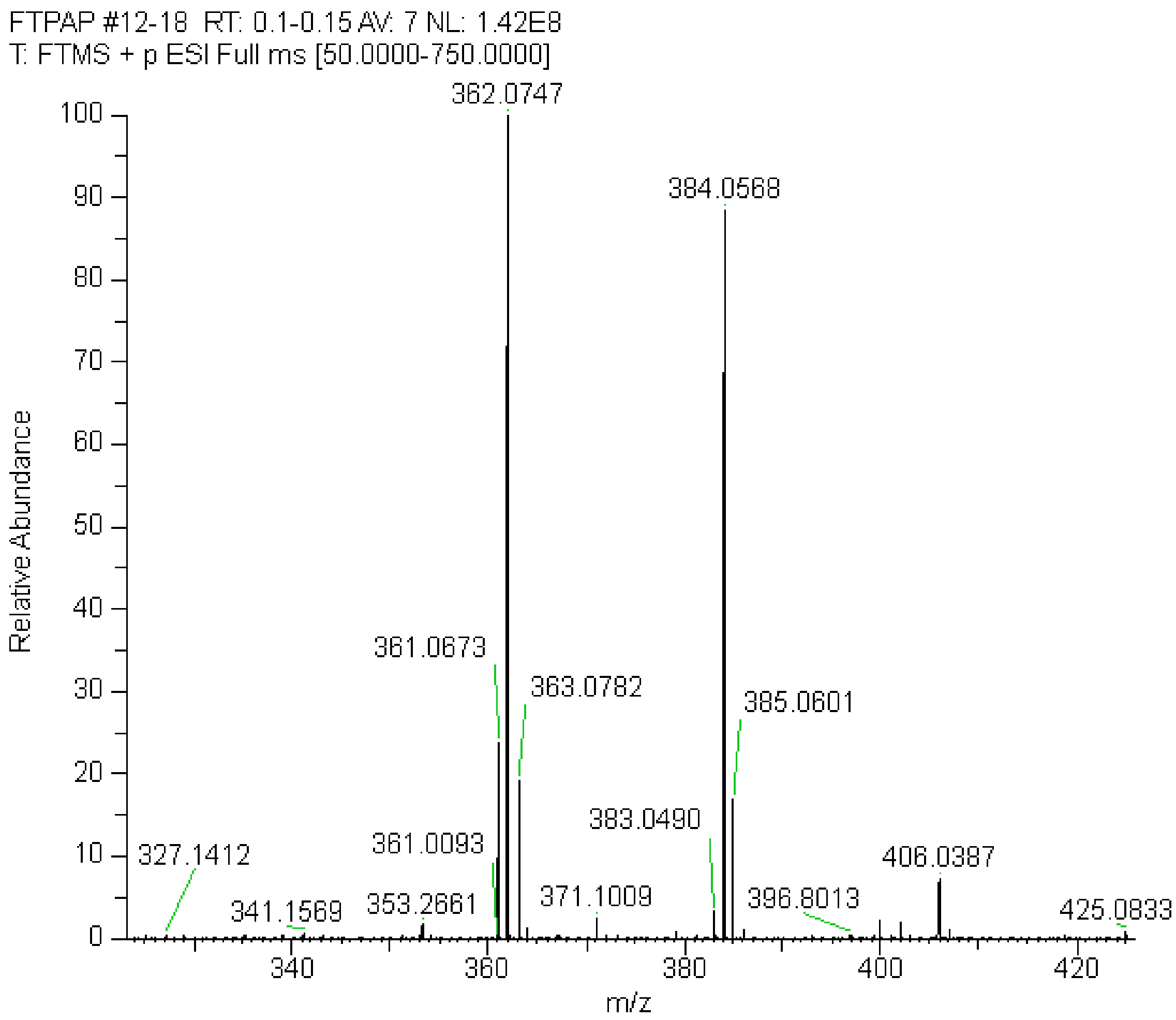

Figure S33. HRMS of (4-(bis(4-fluorophenyl)amino)phenyl)phosphonic acid **(9)**.

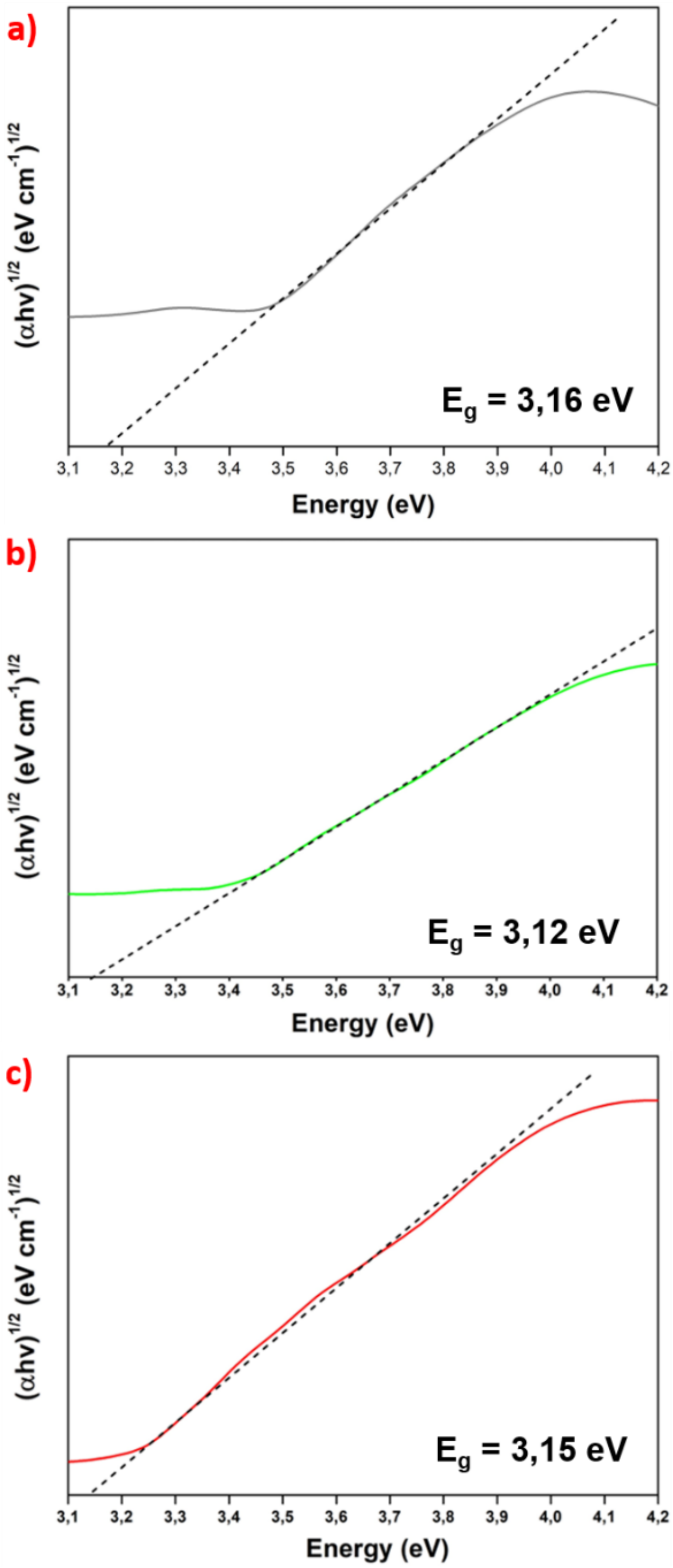

Figure S34. Tauc plots of UV-vis absorption spectra for SAM films. **(a)** TPAP **(b)** F-TPAP and **(c)** MeO-TPAP.

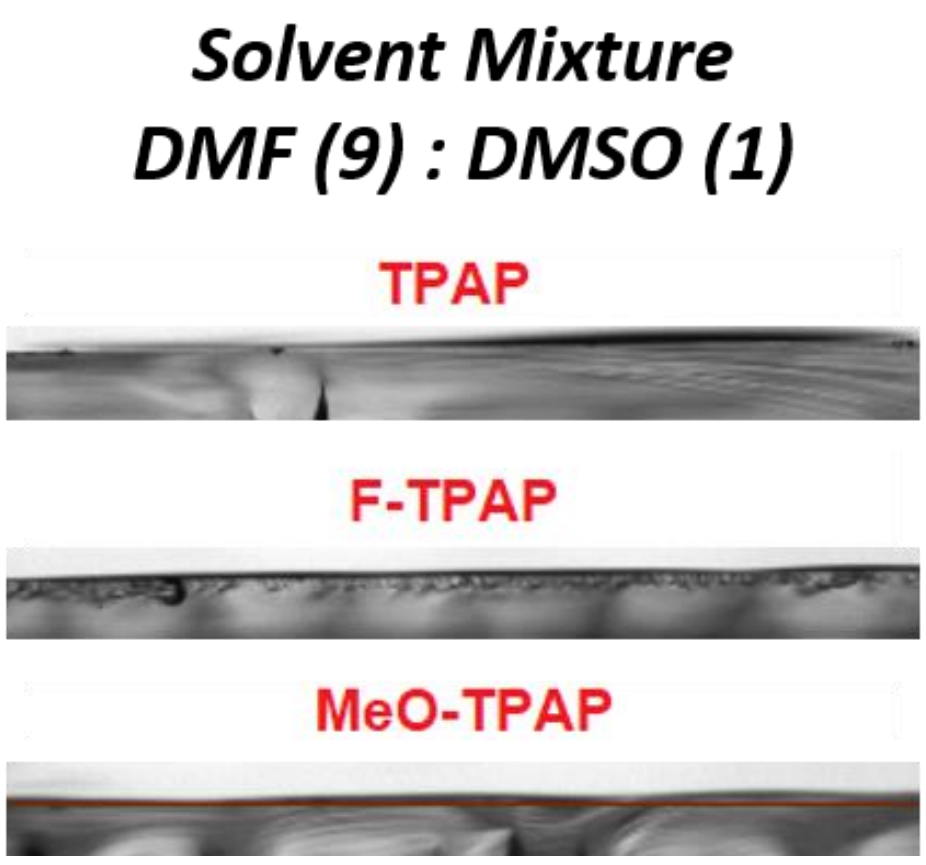

Figure S35. Contact angle measurements of the corresponding SAM-modified substrates with DMF (9):DMSO (1) perovskite solvent mixture.

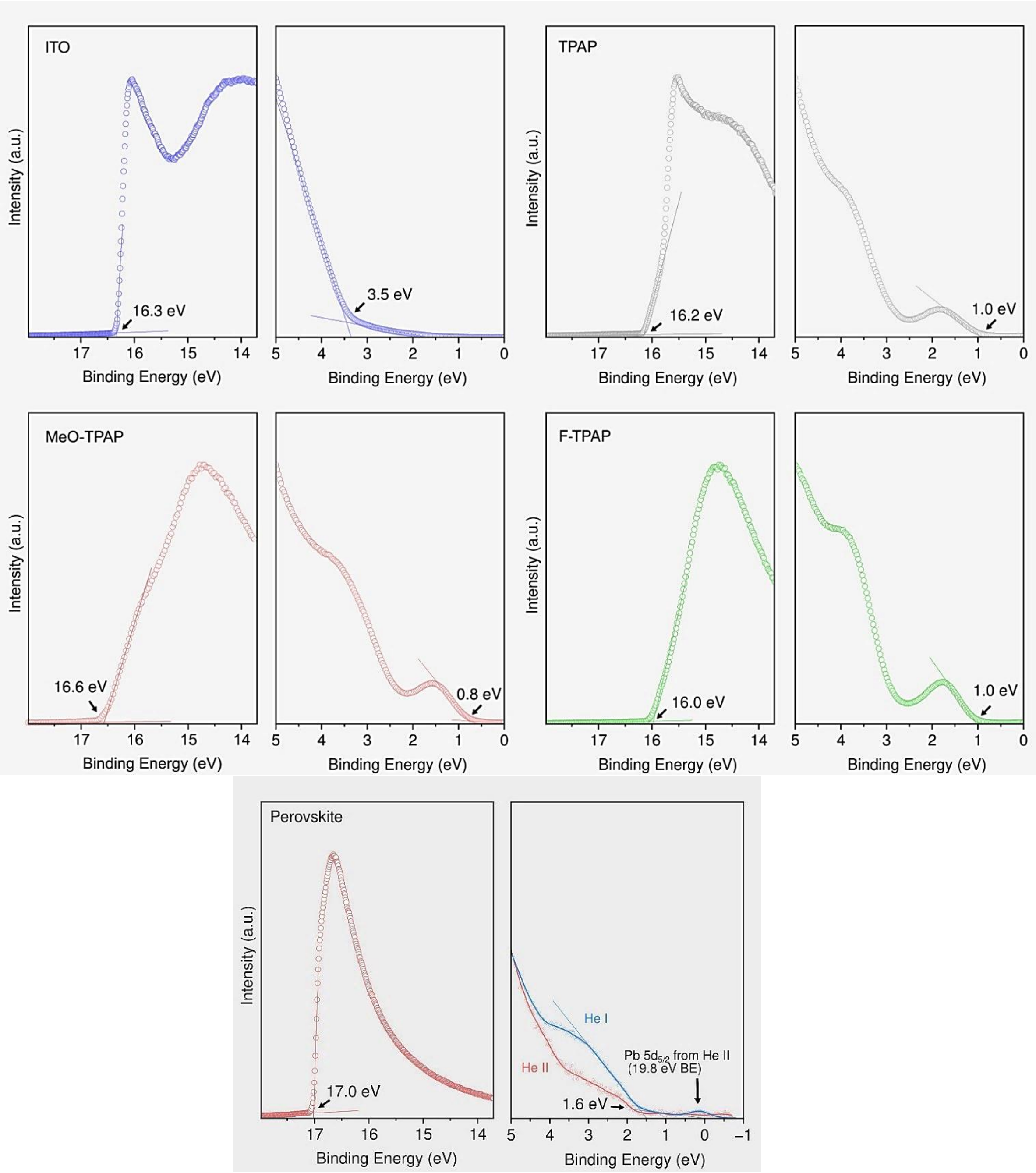

Figure S36. Ultraviolet photoelectron spectra of bare ITO, SAM-modified ITO and perovskite thin films.

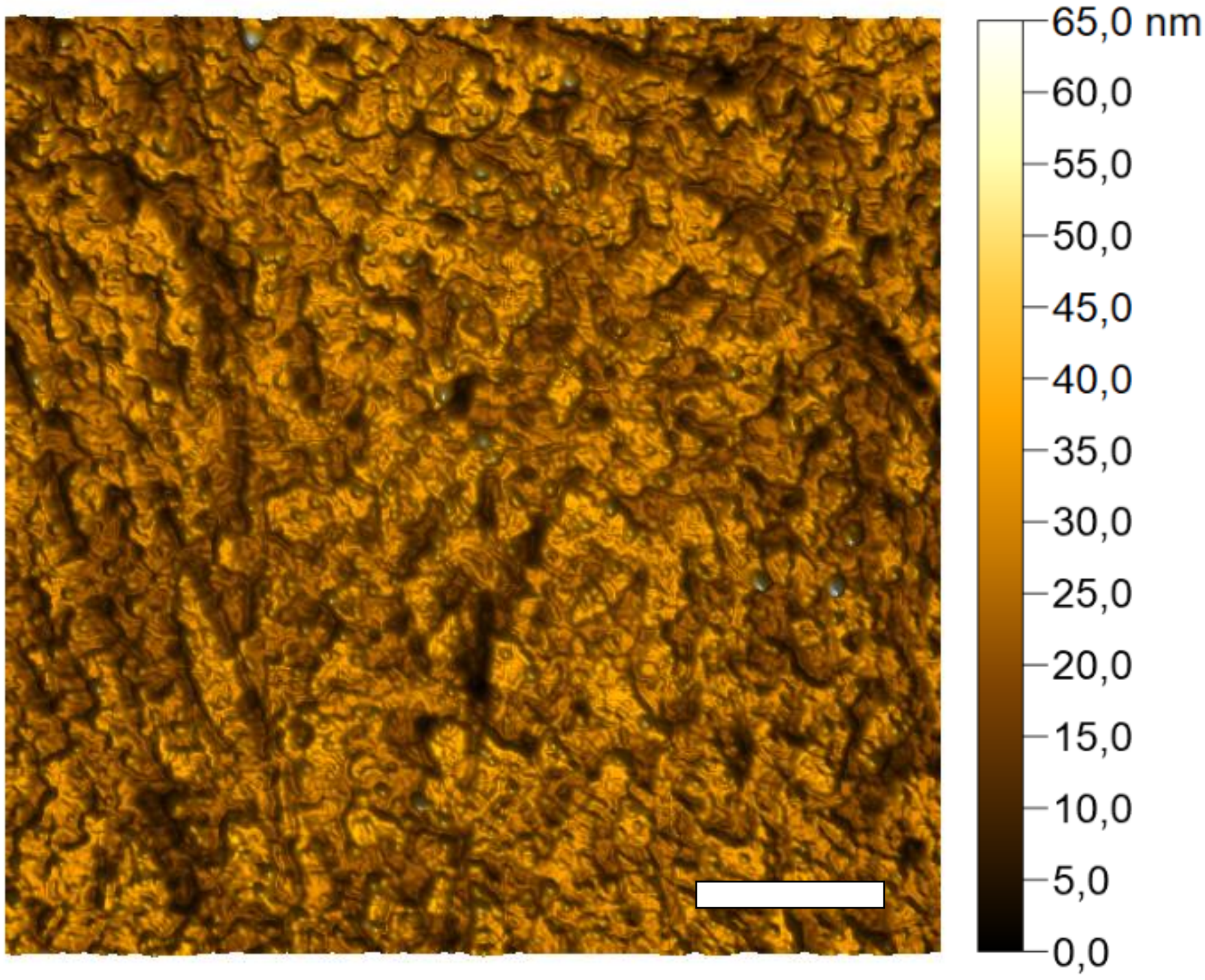

Figure S37. AFM topography of bare ITO substrate.

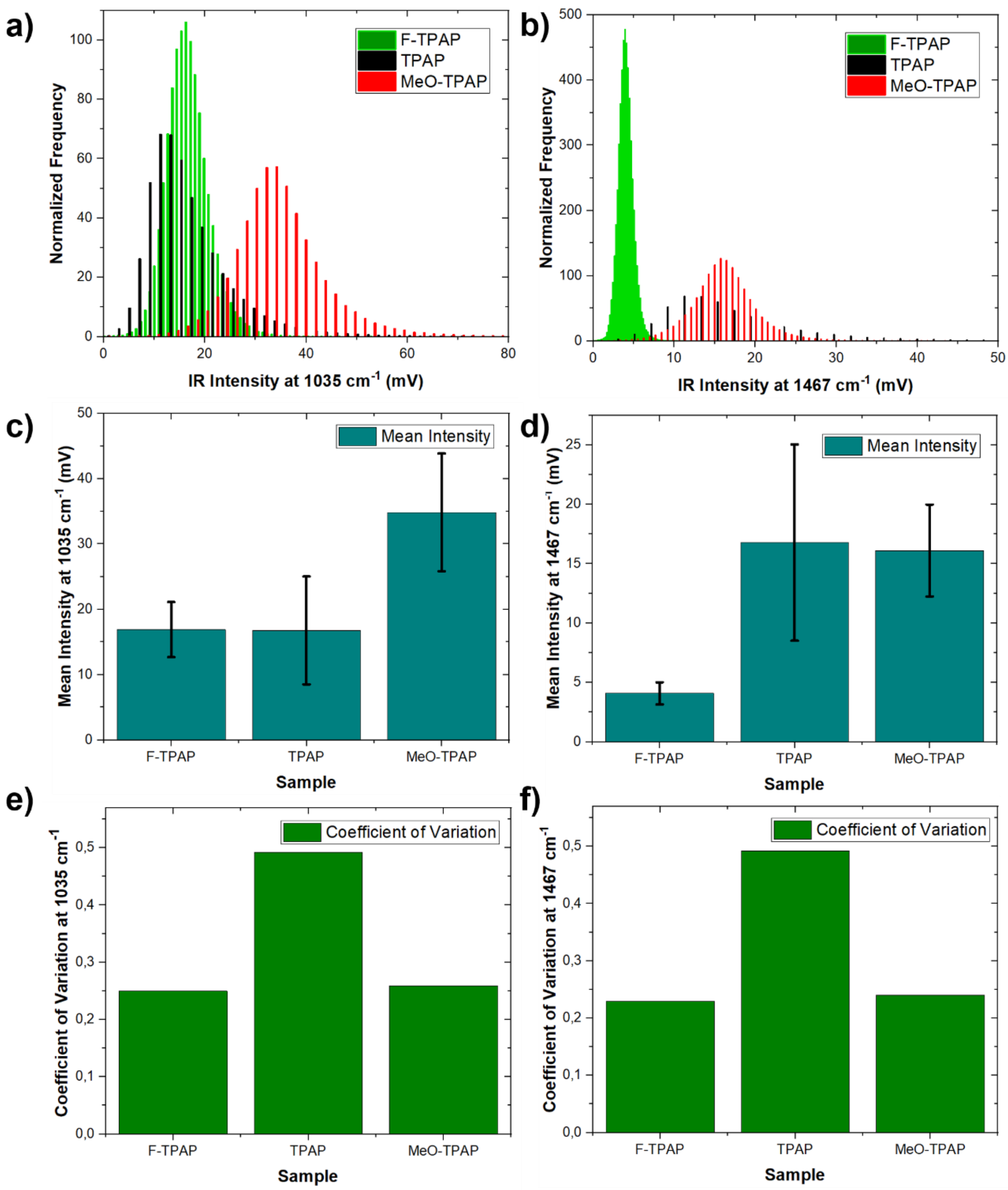

Figure S38. Quantitative AFM–IR analysis of F-TPAP, TPAP, and MeO-TPAP SAMs deposited on ITO. **(a,b)** Normalized pixel-intensity distributions extracted from AFM–IR maps acquired at 1035 cm⁻¹ and 1467 cm⁻¹, respectively. **(c,d)** Mean AFM–IR intensities obtained from the corresponding pixel distributions. Error bars represent one standard deviation. **(e,f)** Coefficients of variation (CV = standard deviation/mean) used as a measure of the spatial heterogeneity of the AFM–IR signal.

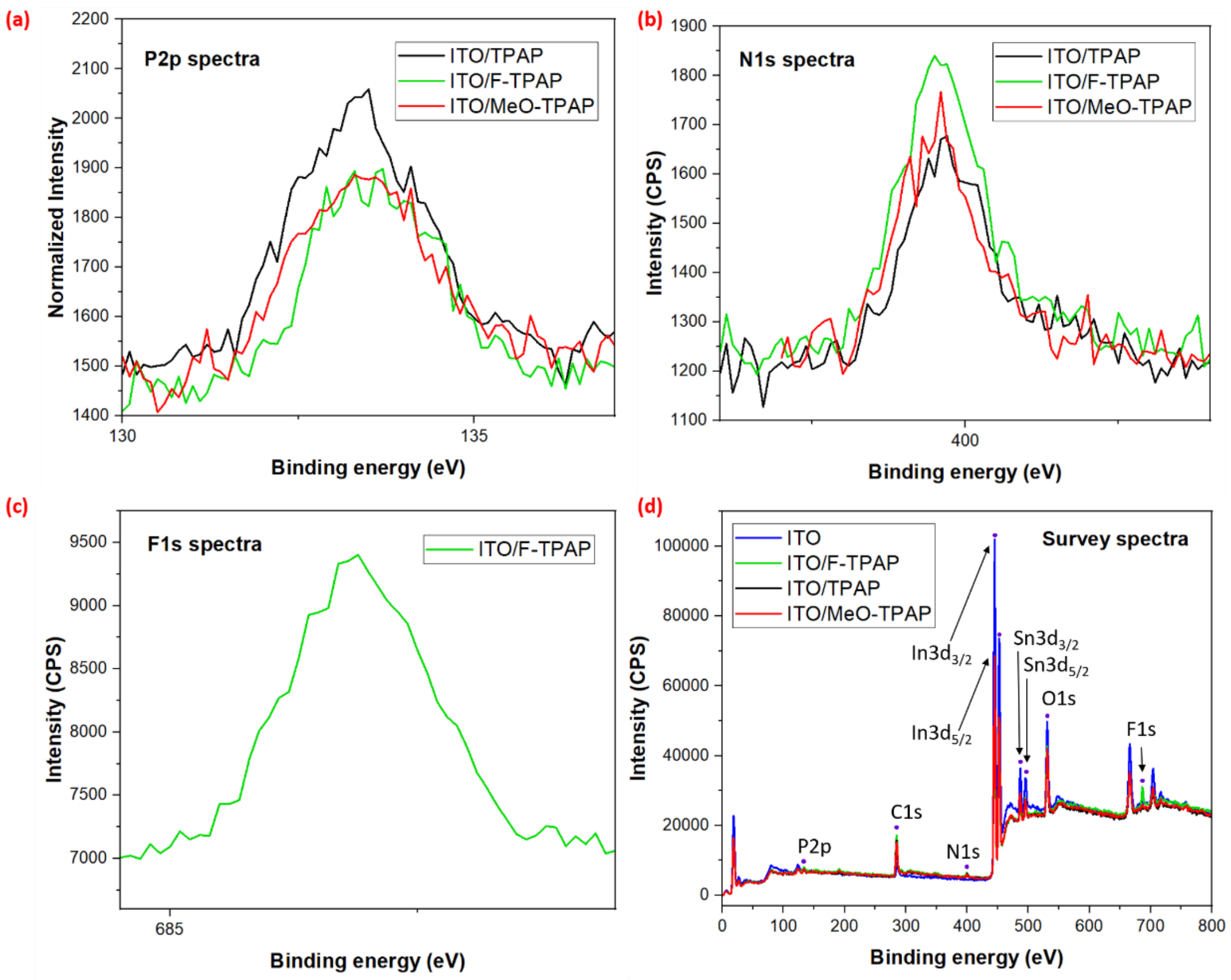

Figure S39. XPS characterization of SAM-modified ITO substrates showing high-resolution spectra of the **(a)** P 2p, **(b)** N 1s, and **(c)** F 1s regions and **(d)** survey spectra.

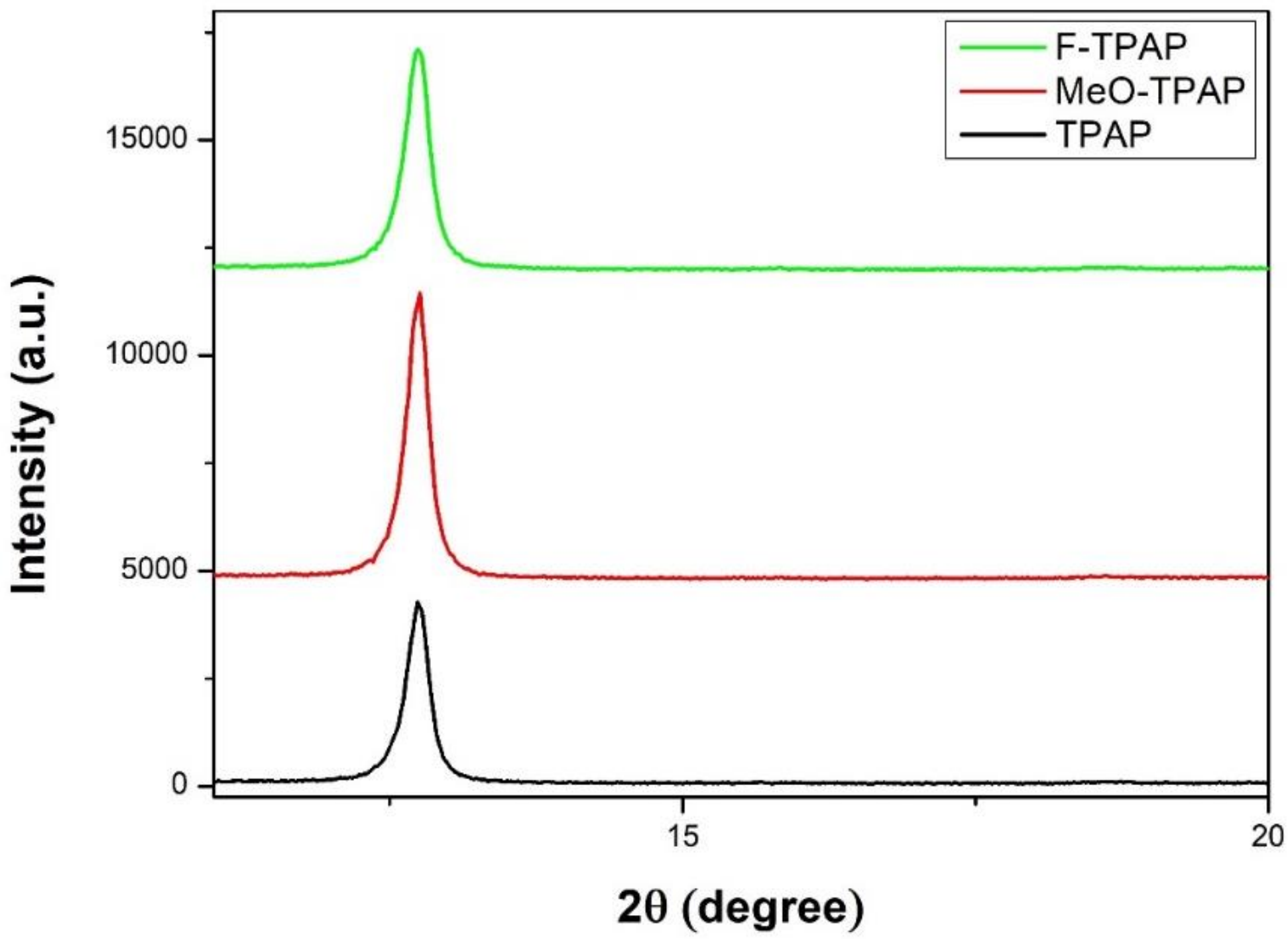

Figure S40. X-ray diffraction (XRD) patterns of PbI_2 films deposited on SAM-modified ITO.

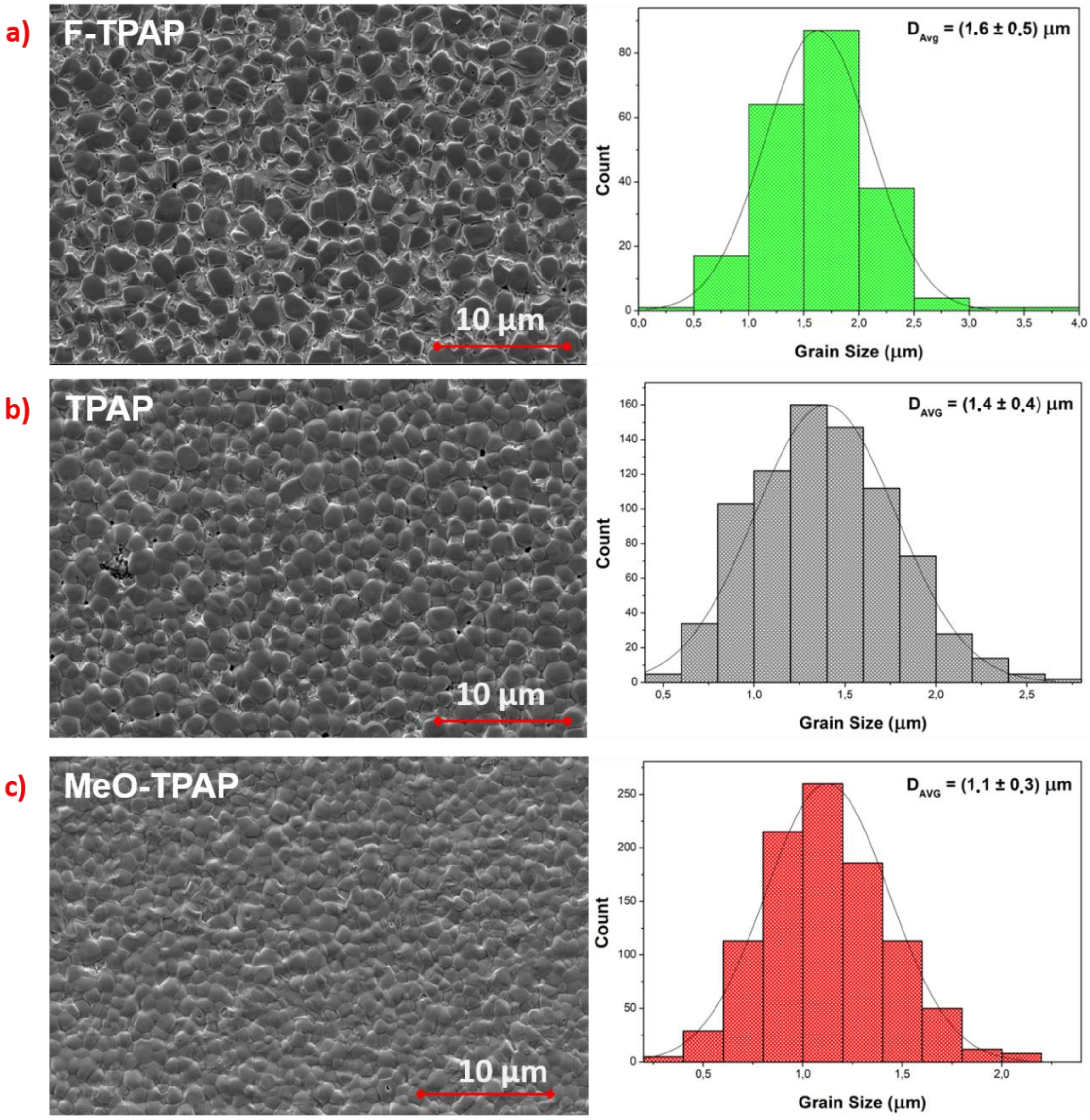

Figure S41. Top-view SEM images of $FAPbI_3$ films deposited on **(a)** TPAP, **(b)** TPAP, and **(c)** MeO-TPAP, with their respective grain size distribution statistics.

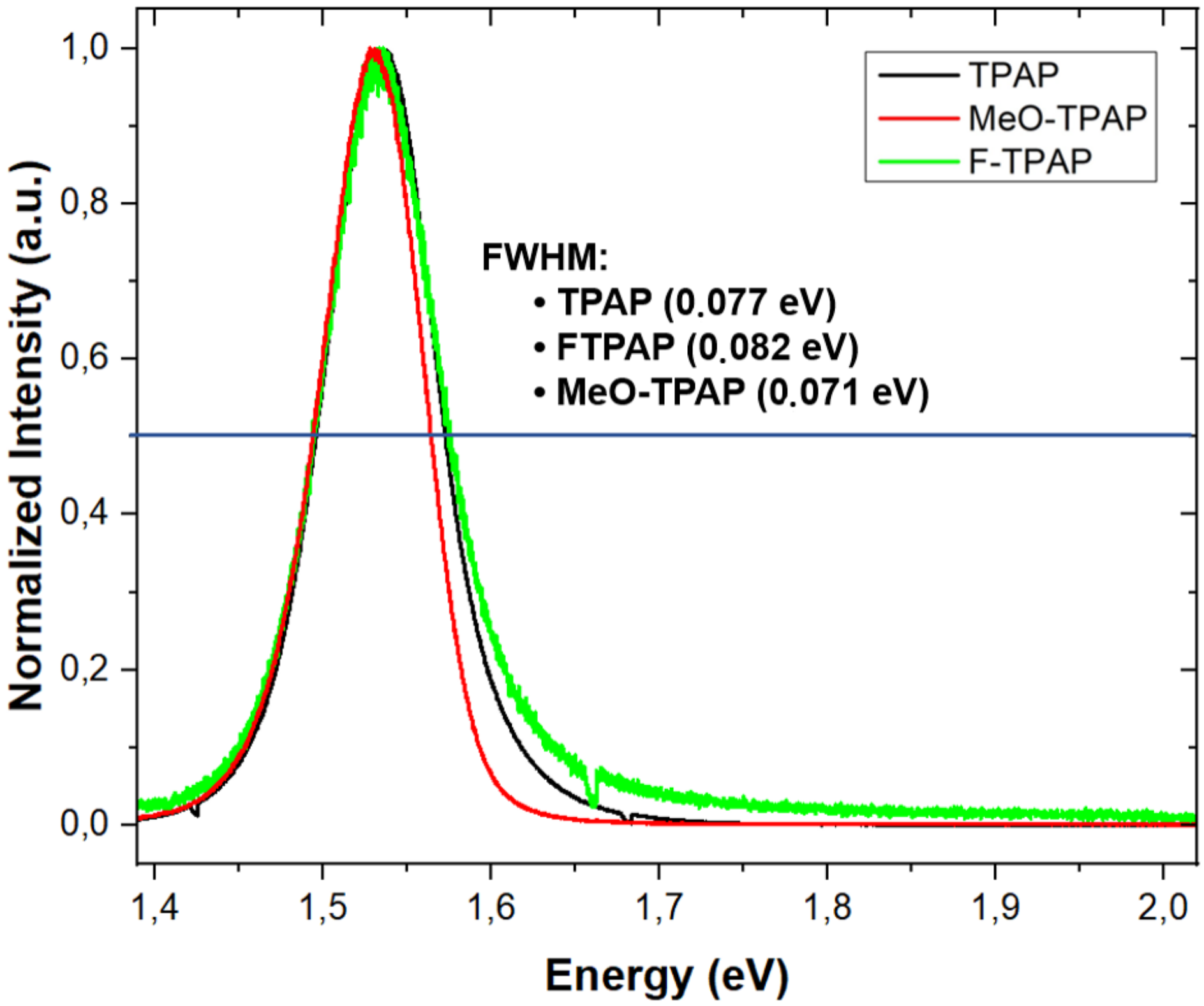

Figure S42. Normalized steady-state PL spectra of $FAPbI_3$ deposited on TPAP, F-TPAP, and MeO-TPAP.

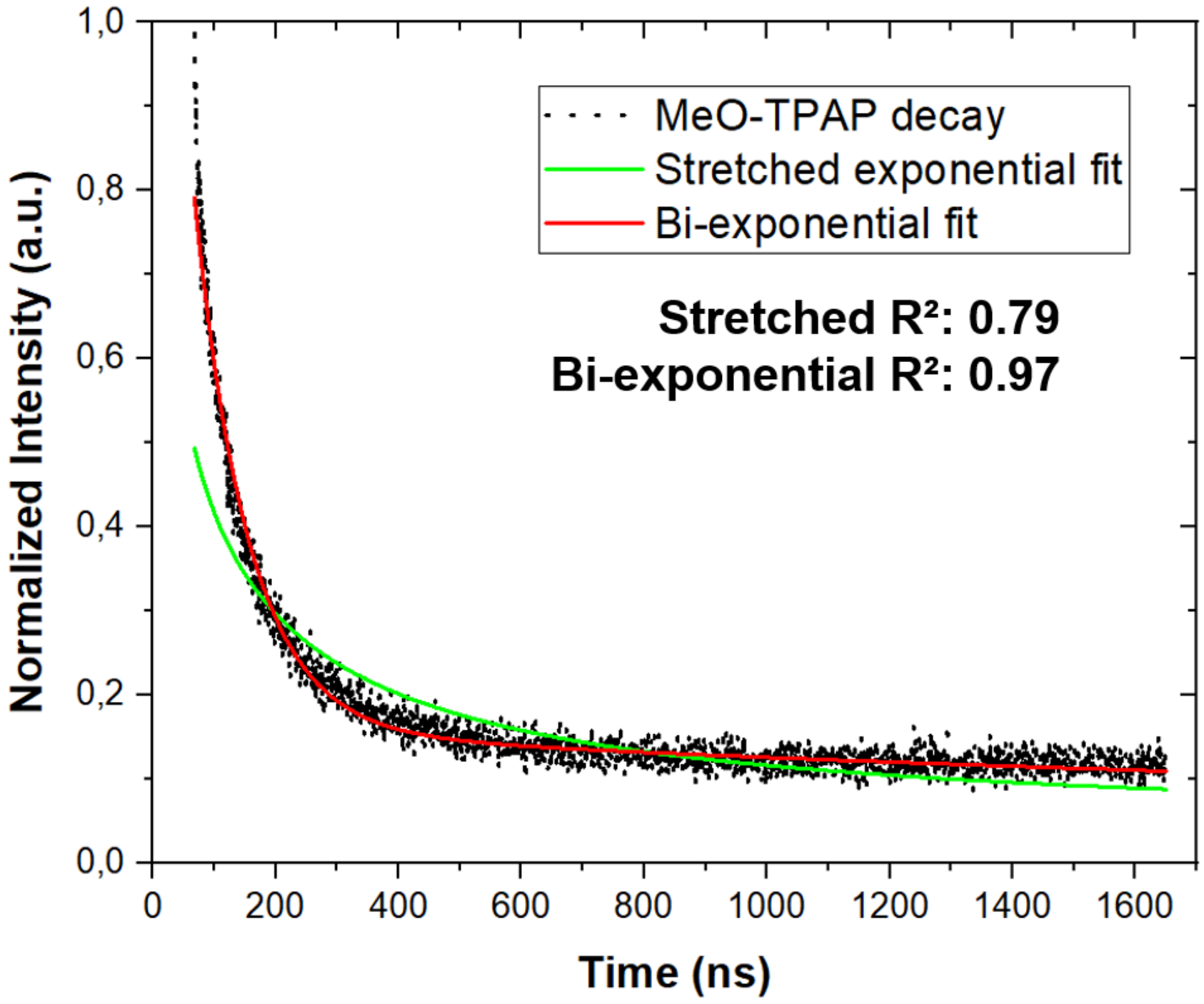

Figure S43. Comparison of stretched-exponential and biexponential fits for the TRPL decay of the ITO/MeO-TPAP/$FAPbI_3$ sample.

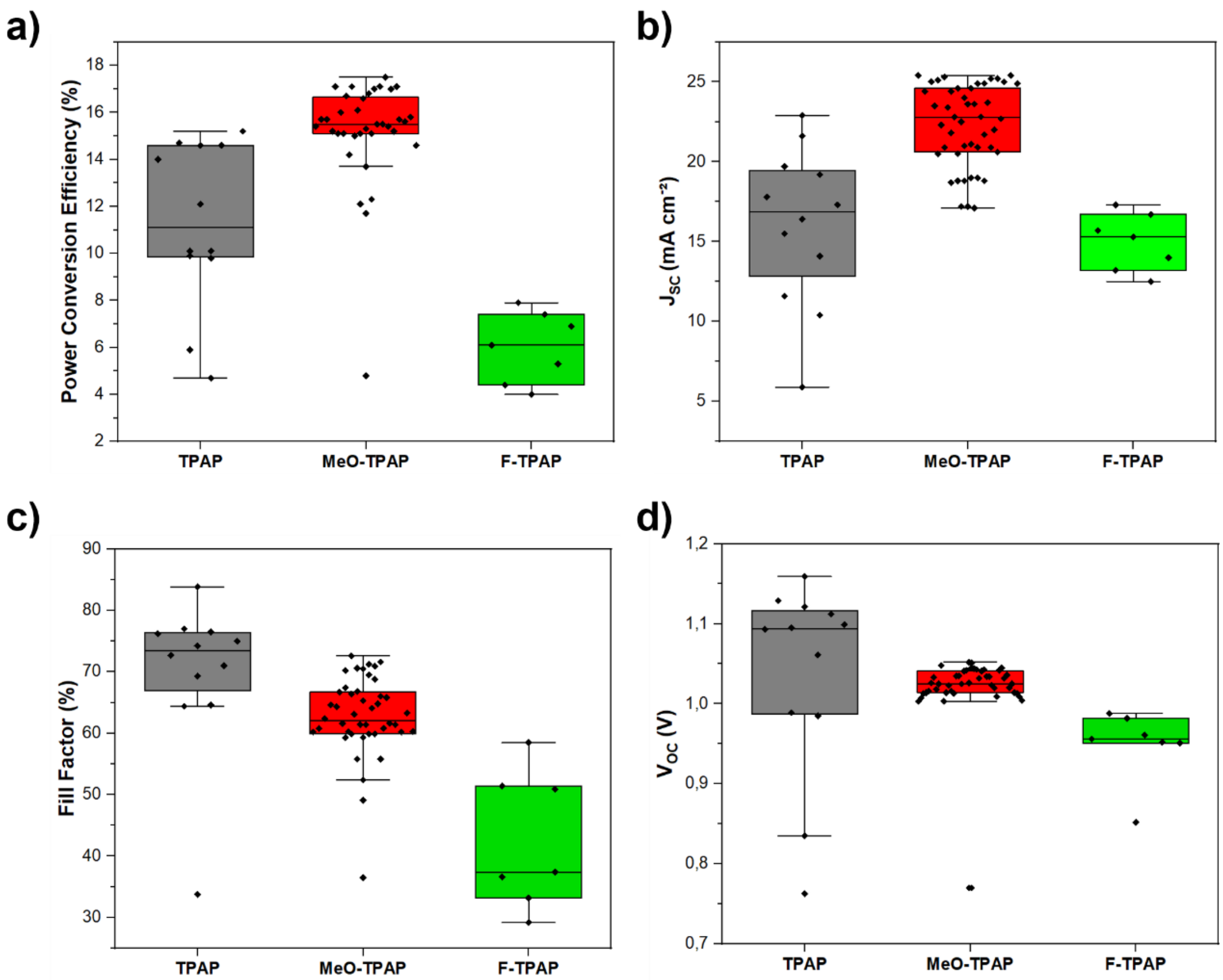

Figure S44. Box plots of the photovoltaic parameters obtained from forward J–V scans of inverted perovskite solar cells based on the synthesized SAMs: **(a)** PCE, **(b)** J_{sc}, **(c)** FF, and **(d)** V_{oc}.

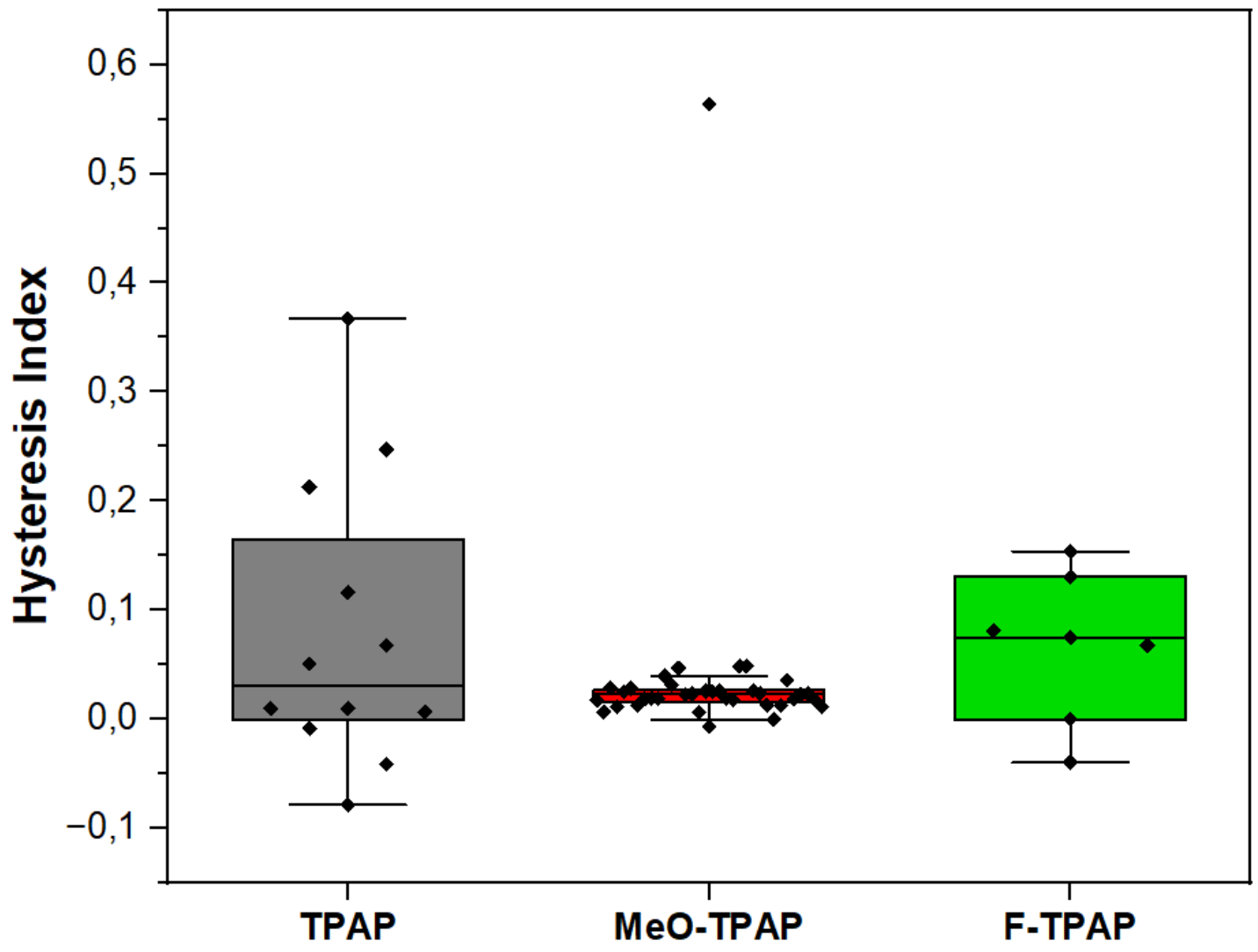

Figure S45. Distribution of hysteresis index (HI) values obtained from functional perovskite solar cells employing F-TPAP, TPAP, and MeO-TPAP as hole-selective layers.

SUPPLEMENTARY TABLES:

Table S1. Energy level parameters obtained from UPS measures of SAM-Modified ITO.

Sample	E_{sec} (eV)	E_{onset} (eV)	WF (eV)	HOMO (ev)	Gap (eV)	LUMO (eV)
**ITO/F-TPAP**	16.0	1.0	5.2	6.2	3.1	3.1
**ITO/TPAP**	16.2	1.0	5.0	6.0	3.2	2.8
**ITO/MeO-TPAP**	16.6	0.8	4.6	5.4	3.2	2.3

E_{sec}: Secondary electron cut-off edge.

E_{onset}: Onset edge.

WF: Work function.

HOMO: Highest occupied molecular orbital.

Gap: Optical band gaps obtained from tauc plot of UV-Vis spectra.

LUMO: Lowest unoccupied molecular orbit.

Table S2. Quantitative analysis of AFM–IR chemical maps obtained from pixel intensity distributions extracted from maps acquired at 1035 and 1467 cm^{-1} for F-TPAP, TPAP, and MeO-TPAP SAMs deposited on ITO.

Signal	Sample	Mean Intensity (mV)	Std. Dev. (mV)	Median (mV)	CV
**1035 cm^{-1}**	**F-TPAP**	16.9	4.2	16.6	0.250
**1035 cm^{-1}**	**TPAP**	16.8	8.3	14.9	0.492
**1035 cm^{-1}**	**MeO-TPAP**	34.8	9.0	33.9	0.258
**1467 cm^{-1}**	**F-TPAP**	4.1	0.9	4.0	0.230
**1467 cm^{-1}**	**TPAP**	16.8	8.3	14.9	0.492
**1467 cm^{-1}**	**MeO-TPAP**	16.1	3.9	16.0	0.240

CV: Coefficients of variation (CV = standard deviation/mean)

Table S3. Apparent thicknesses of SAM-modified ITO substrates estimated by XPS.

Sample	In 3d (eV)	N 1s (eV)	Estimated Thickness (nm)
**ITO/F-TPAP**	12524	2327	2.2 ± 0.1
**ITO/TPAP**	13536	1890	2.1 ± 0.1
**ITO/MeO-TPAP**	13769	1332	1.7 ± 0.1

Effective attenuation lengths (EALs) were calculated according to the NIST formalism,[S2] and a normal photoelectron takeoff angle was considered.

Table S4. Grain size distributions of $FAPbI_3$ films deposited over different SAMs.

Sample	D_{Avg} (µm)	N (un.)
**ITO/F-TPAP/FAPbI₃**	1.4 ± 0.4	805
**ITO/TPAP/FAPbI₃**	1.6 ± 0.5	213
**ITO/MeO-TPAP/FAPbI₃**	1.1 ± 0.3	991

D_{Avg} : Average grain size

N: Number of grains analyzed

Table S5. Parameters used to fit the TRPL measurement data of SAM-modified ITO.

Sample	A_1 (%)	τ_1 (ns)	A_2 (%)	τ_2 (ns)	β	R^2 (%)
**ITO/F-TPAP/FAPbI₃ (streched)**	-	1179.0	-	-	0.856	98.42
**ITO/TPAP/FAPbI₃ (streched)**	-	1514.6	-	-	0.782	97.69
**ITO/MeO-TPAP/FAPbI₃ (bi-exponential)**	1.37	87.3	0.11	2658.4	-	97.54

τ: Carrier lifetime

***A*:** Relative amplitude

β: Stretching parameter

Table S6. Average and champion photovoltaic parameters from reverse measurements of perovskite solar cells based on different SAMs.

Sample	V_{OC} (V)	J_{SC} (mA cm^{-2})	FF (%)	PCE (%)
**F-TPAP**	0.95 ± 0.07 (0.99)	14.69 ± 1.39 (15.5)	45.94 ± 8.85 (55.8)	6.43 ± 1.57 (8.6)
**TPAP**	1.03 ± 0.13 (1.00)	16.11 ± 4.36 (22.7)	63.20 ± 13.65 (67.0)	10.59 ± 4.06 (15.1)
**MeO-TPAP**	1.04 ± 0.01 (1.02)	22.89 ± 1.97 (24.8)	66.16 ± 4.12 (69.9)	15.70 ± 1.58 (17.7)

Table S7. Average and champion photovoltaic parameters from forward measurements of perovskite solar cells based on different SAMs.

Sample	V_{OC} (V)	J_{SC} (mA cm^{-2})	FF (%)	PCE (%)
**F-TPAP**	0.95 ± 0.05 (0.99)	14.95 ± 1.79 (15.7)	42.46 ± 11.02 (50.9)	6.00 ± 1.49 (7.9)
**TPAP**	1.04 ± 0.12 (0.99)	16.03 ± 4.90 (21.6)	69.87 ± 12.59 (71.0)	11.31 ± 3.52 (15.2)
**MeO-TPAP**	1.03 ± 0.05 (1.02)	23.14 ± 2.08 (25.0)	63.78 ± 6.78 (68.8)	15.19 ± 2.25 (17.5)

V_{OC}: Open circuit voltage

J_{SC}: Short circuit current

FF: Fill factor

PCE: Power conversion efficiency

Table S.8 Median hysteresis index (HI) and corresponding interquartile range (IQR) for functional perovskite solar cells employing synthetized SAMs as hole-selective layers.

Sample	HI (median)	IQR
**F-TPAP**	0.08	0.07
**TPAP**	0.06	0.13
**MeO-TPAP**	0.02	0.01

SUPPLEMENTARY REFERENCES

[S1] Bhunia, Mrinal; Sahoo, Sumeet Ranjan; Das, Arpan; Ahmed, Jasimuddin; P., Sreejyothi; Mandal, Swadhin K. Transition metal-free catalytic reduction of primary amides using an abnormal NHC based potassium complex: integrating nucleophilicity with Lewis acidic activation. *Chemical Science* **2020**, 11, 1848-1854. DOI: https://doi.org/10.1039/C9SC05953A

[S2] C. J. Powell; A. Jablonski. NIST Electron Effective-Attenuation-Length Database Version 1.3. Journal of Physical and Chemical Reference Data 2020, *49* (3), 033104. DOI: 10.1063/5.0008576.
